# Walking the tightrope of justifiable decision‑making: An exploratory qualitative study identifying barriers and solutions to efficient safety reporting

**DOI:** 10.1371/journal.pone.0354806

**Published:** 2026-07-30

**Authors:** Jemima Thompson, Macey L. Murray, Matthew R. Sydes, Annabelle South, Sharon B. Love

**Affiliations:** 1 UCL Innovative Clinical Trials Unit, Institute of Clinical Trials and Methodology, UCL, London, United Kingdom; 2 Health Data Research UK, London, United Kingdom; 3 Data for R&D, Transformation Directorate, NHS England, London, United Kingdom; Durham University, UNITED KINGDOM OF GREAT BRITAIN AND NORTHERN IRELAND

## Abstract

**Background:**

Safety reporting is integral to clinical trial conduct, aiming to protect the rights and safety of trial participants and future patients. Over time, reporting safety events has become increasingly complex, leading to inefficiencies which place extra burden on trial staff and potentially impact patient safety. Attempts have been made to streamline these processes. However, effecting change in practice is challenging. Changes to UK trial regulations introduced in 2025 may reduce inefficiencies. To incorporate these changes effectively, we need to understand what barriers exist for their implementation. This study aimed to identify barriers and solutions to efficient safety reporting processes from the perspective of staff working at registered academic trials units in the UK, with a view to developing recommendations to enable the delivery of more efficient safety reporting practices.

**Method:**

This was an exploratory, observational, qualitative focus group study of trials unit staff across the UK. Verbatim transcripts of the focus group discussions were analysed using Reflexive Thematic Analysis.

**Results:**

Twenty-three CTU staff participated in four focus groups. One over-arching theme was generated from the analysis, “*Walking on a tightrope: Making justifiable decisions”.* Participants felt that they were performing a balancing act between efficient, risk-proportionate safety reporting and risk-aversion. Possible solutions included clarification of expectations and improved transparency, and improved training and knowledge building.

**Discussion:**

Despite efforts to streamline safety reporting processes, problems persist with the implementation of risk-proportionate approaches. Concerns that decisions will be deemed inadequate and impact patient safety and well-being make decision-making a difficult balancing-act. Confident decision-making by trial practitioners can be facilitated through access to resources and training provided by regulators, supported by tools and case studies. Trials unit level actions, such as mentoring schemes or platforms to share knowledge and learning, can support knowledge building and confidence development.

## Introduction

Safety reporting plays a key role in trial conduct. According to ICH Good Clinical Practice (GCP), triallists are required to capture adverse event information, the primary aim of which is to protect the rights and safety of trial participants and future patients, in both the short‑ and long‑term [1]. Furthermore, pharmacovigilance is a requirement of Clinical Trials of Investigational Medicinal Products (CTIMPs); defined by the World Health Organisation (WHO) as *“the science and activities relating to the detection, assessment, understanding and prevention of adverse effects or any other drug related problem”* [2]

Over time, safety reporting requirements for CTIMP trials have become increasingly complex and the number of tasks to be performed and administrative demands to be met has risen [3,4]. This is due, in part, to changes in regulations following previous instances of serious harm, such as that experienced by six volunteers who participated in the TGN1412 Phase I trial in 2006 [5]. Whilst those changes were necessary to ensure patient safety, they have triggered inefficiencies in safety reporting processes across the clinical trials landscape.

Conservative approaches to safety reporting can lead to excess resource burden. Academically-led trials often have limited financial backing and fewer resources at their disposal meaning that the extra safety reporting may cause trials not to finish on time, or at all [6]. Furthermore, and most importantly, it can lead to the generation of excessive reports, obfuscating important safety signals and, ultimately, having a diluting effect on the overall safety profile of the product [7–10]. Conversely, making pragmatic choices about safety reporting may mean that some lower grade events which impact patients’ quality-of-life are not captured. As highlighted by Seruga and colleagues, who discussed the under-reporting of harms in clinical trials, low-grade diarrhoea lasting several weeks may be more bothersome for patients than a higher-grade bout of diarrhoea lasting just a few days [11]. Furthermore, as with over-reporting, under-reporting can also put patients’ safety at risk if events of significance remain uncaptured [8]. These issues make the need for the enactment of effective risk-proportionate approaches to safety reporting necessary and helpful.

Internationally, attempts have been made to streamline safety reporting processes, yet implementing these changes has proven to be challenging for triallists. In the USA, for example, in 2011, the Food and Drug Administration (FDA) introduced the final rule, which included the publication of guidance which clarified reporting requirements for serious and suspected unexpected adverse reactions [12,13]. Despite the well-written guidance and the presence of reasoned rationales for the changes [13], Perez et al’s study consisting of two online surveys and 20 in-depth interviews with trial staff, revealed that there was little to no reduction in the volume of work produced [14]. This was due, in large part, to Sponsor concerns about the consequences should event causality be misjudged. Similarly, following a survey conducted at the 2015 American Society of Clinical Oncology (ASCO) and American Council on Consumer Interest (ACCI) meetings, Vose et al also found that Sponsors continued to over report safety events following the introduction of the final rule, leading to research sites being overwhelmed and struggling to untangle what information is relevant [15].

Risk-proportionality as a philosophy of safety reporting was first introduced as guidance for trial staff in the UK in 2011, and last updated in 2021, through the MRC/DH/MHRA Joint Project [16]. The “*Risk-adapted Approaches to the Management of Clinical Trials of Investigational Medicinal Products (IMPs)”* [16] guidance encourages trialists to use the risk profile of an IMP to determine which events need to be reported. Following the UK’s withdrawal from the European Union in 2020, the MHRA has diverged from European Clinical Trials Regulations (CTR 536/2014), accelerating the shift of risk-proportionality from optional guidance to legislative requirement. This shift initially increased administrative load for triallists; for example, by requiring staff to use a standalone MHRA submissions portal rather than EudraVigilance. The heterogeneity of requirements and reporting systems across jurisdictions makes harmonised, streamlined reporting a challenge [17]. Inconsistencies such as these have been cited as a cause of ongoing frustration and confusion internationally [18,19]. Differences in how UK academic trials units interpret the regulations, and the resulting variation in Standard Operating Procedures (SOPs), also make it difficult to implement consistent and efficient safety reporting processes [20].

Since the UK left the European Union, further changes have been made to guidance and legislation in the UK which aimed to reduce workload burden for trial staff; for example, in 2024, the Health Research Authority (HRA) announced that trials approved by an NHS Research Ethics Committee (REC) would no longer need to submit annual progress reports [21]. Moreover, in April 2025, the biggest reform to UK clinical trials in 20 years was signed into law and came into force on 28^th^ April 2026 [22]. The changes include updates to safety reporting processes which intend to further reduce administrative burden and support the implementation of risk-proportionate approaches. For example, under the new legislation, SUSARs and Annual Safety Reports need only to be reported to the MHRA; duplicative reporting to the Research Ethics Committee (REC) is no longer required [23]. Safety reporting has been scaled back and simplified for trials categorised as low-risk or evaluating well-established treatments [23]. The introduction of these changes is sufficiently recent that any impact on triallists’ workload, and barriers and facilitators to their implementation has yet to be explored.

Adequate training and knowledge of safety reporting requirements are key foundations to accurate and efficient enactment of guidance and regulations. However, knowledge gaps have often been found among trial staff. A study by Riordan and colleagues conducted in Ireland exploring the attitudes, knowledge and practice of health professionals working in clinical trials found that knowledge gaps were a frequently reported barrier to the reporting of Adverse Drug Reactions (ADRs) [24]. This occurred alongside a lack of practical guidance and inconsistency about how to complete ADR reporting, leading to significant under-reporting [24]. In the USA, Miller et al found that a lack of general safety reporting training, and a lack of protocol specific training were barriers to effective safety reporting in paediatric oncology trials [25]. Similarly, a study conducted in Turkey suggested that gaps in knowledge and understanding of safety reporting and the regulations and ethical considerations which underpin them can lead to under-reporting [26]. In the UK, during Pharmacovigilance Metrics Inspections undertaken between 2015–2021, the MHRA found that staff were frequently working on non-commercial trials without having completed adequate trial specific training such as trial protocols or Investigator Brochures (IBs) or Good Clinical Practice (GCP) [27].

To support the implementation of the new regulations, the recent Flourishing And Clinical Trials Staff (FACTS) study devised and is circulating guidance for trial staff [28]. Their study highlighted that whilst knowledge is important, the “personal resources” of staff, such as their time, also play an important role in good safety reporting practices. Training and learning should, therefore, be role‑specific and proportionate to delegated responsibilities for the role.

In summary,there have been several changes to safety reporting in the UK in recent years. In light of April 2026’s regulatory changes, understanding the current barriers to effective, efficient safety reporting is important to determine where these changes are most likely to be of value for trialists. Notably, this study captures these views from the perspective of UK-based trial staff who often manage the administrative burden of safety reporting, but whose voices are often underheard. Therefore, this study aimed to identify barriers and solutions to efficient safety reporting from the perspective of staff working at registered academic trials units in the UK, with a view to developing recommendations to enable the delivery of more efficient safety reporting practices.

## Methods

This was an exploratory, observational, qualitative study of academic trials unit staff in the UK with experience of safety reporting for CTIMP trials. Ethical approval was obtained from the UCL REC (Project ID: 28019/001). This study is reported in accordance with Standards for Reporting Qualitative Research (SRQR) guidelines [29] (S1 Appendix).

### Ontological and epistemological position

This study is positioned within Critical Realist ontology, whereby participants accounts were understood as mediated reflections of [their] reality shaped by their social and political context [30]. Epistemologically, this study adopts a contextualist position, recognising that meanings are situated and perspectival, and that knowledge is co-produced through the relationship between researcher and participants [30].

### Position of the researcher

JT was the primary researcher leading the study design, data collection and analysis. She is a Research Fellow in Trial Conduct Methodology at the UCL Innovative Clinical Trials Unit (formerly the MRC Clinical Trials Unit at UCL). JT has previously worked in clinical trials in various disease areas as a researcher in the NHS and as a Trial Manager at UCL. She has direct experience of safety reporting both in terms of identifying (Serious) Adverse Events, receiving reports from sites, and reporting safety events to regulators. This experience means that she has an understanding of the processes and terminology and therefore held opinions on the efficiency of safety reporting prior to the design and conduct of this study. JT has also previously worked directly with some of the participants. This means that she was aware of some of the views of these participants before the focus groups began, and this may have impacted her interpretation of some of their contributions.

Information about the positionality of the other members of the research team can be found in the “Authors’ Information” (S2 Appendix).

### Participants, recruitment and informed consent

Sample size was determined based on Malterud et al’s concept of information power, rather than focusing on recruiting a specific number of participants or reaching the commonly cited concept of thematic saturation [31]. Following the principle of information power, sample size was determined relative to key dimensions of the study. The aim of the study was narrow, focusing on safety reporting from a UK academic trial staff perspective, with a view to developing recommendations. The sample was highly specific, drawn from trial staff working in registered trials units across the UK; a group who share regulatory requirements, organisational structure and safety-reporting responsibilities. Participants were recruited using convenience sampling methods. This approach allowed for flexibility and faster recruitment, leading to rapid and cost-effective data collection, which is important as staff availability for participation is limited. It also helped to capture the views of those with expert operational perspectives, which was more valuable than achieving representativeness across all trial staff. In alignment with critical realist ontology and contextualism, this study aimed for transferability of findings, rather than generalisability. Whilst the specific events experienced and described by the participants may be unique to them, the underlying mechanisms are likely to operate similarly in comparable contexts. This is because, despite differences in procedures between trials and institutions, the underpinning regulatory requirements are the same across the UK. Thus, the value of this analysis lies in its power to capture nuanced explanations of the phenomena identified that can inform broader understandings of how to address the issue of inefficiency in safety reporting in the UK.

The initial sample of 9 participants across two focus groups included staff from a range of roles, both clinical and operational, from a single institute in England. The coding process was initiated after these discussions, during which time it became apparent that the data were not rich enough to meet the aims of the study. Several of the issues that arose as issues with safety reporting appeared to be specific to the institution where participants were based, which raised questions about information power and transferability of findings. This concern was discussed with members of the research team, and it was agreed that further focus groups should be conducted with trial staff from other institutes across the UK. A further two focus groups were conducted and the data generated from the increased sample size provided the richness of data required to meet the study aims, improving the robustness and transferability of findings.

The study design and topic guide were informed by existing literature on pharmacovigilance practices, operational guidance and regulatory requirements, providing a strong theoretical foundation that enhanced information power. JT is an experienced qualitative researcher, with a working knowledge of safety reporting processes and legislation in the UK. The quality of the dialogue in the focus groups was good, as was the rapport between participants, who often took the opportunity to elaborate on points made by one another. Participants also asked each other questions to clarify points and gain better understanding of one another’s positions. Where views were divergent, the conversation still flowed in a respectful and understanding manner. Little prompting was needed to address the points highlighted as part of the semi-structured topic guide. Finally, data were analysed using a cross-case analysis approach, capturing a snapshot of specific phenomena experienced by the study participants. The three focus groups included in this study is sufficient to support this approach. Taking these factors into account and following discussion with the research team it was felt that sufficient information power was reached, and the sample size was appropriate to meet the study aim and generate robust, meaningful findings.

Trial staff were approached via email, newsletters and recruitment posters. Those interested in participating self-identified via email or approached JT in person or via email. The Participant Information Sheet was emailed to those who expressed interest. Potential participants were given at least 48 hours to read the information and ask questions before providing their written informed consent.

### Data collection

Participants were all asked to complete a short demographics questionnaire on Microsoft Forms [32] prior to the focus group discussions.

A semi-structured topic guide, with prompt questions, was initially developed *a priori* by JT and reviewed by SL. The topic guide was designed to ensure that the questions were not leading participants to provide answers that aligned with JTs pre-conceived views on safety reporting processes by grounding questions in the aims of the study. The first version of the topic guide was used for the first two focus groups and was subsequently amended for the remaining two, based on reflections from the outcomes of the initial two discussions (S3 Appendix).

Four online focus groups were conducted between 01-July-2024 and 04-March-2025, via Microsoft Teams [33]. All focus group discussions were video recorded and auto-transcribed in Microsoft Teams. The automatically generated transcripts were cleaned and pseudonymised using intelligent verbatim transcription principles.

JT entered the discussions with her own thoughts and ideas about where problems with safety reporting may lay. These thoughts were mitigated by following the topic guide and asking open questions that did not lead participants to answer in a certain way. During the discussion, there were topics of discussion and statements that JT could relate to and agreed with. This was used as an opportunity to build rapport with participants and open up further discussion on the topics. Expressions of alternative viewpoints were also encouraged by asking the participants, in an open way, whether they had any other thoughts or views or whether they agreed or disagreed with previous statements or opinions.

Each focus group lasted around 90 minutes, with the first group co-facilitated by KR and the remaining three by ST. KR and ST were both completing PhDs in areas of trial conduct at the Innovative Clinical Trials Unit. ST had previous experience as a Trial Manager and KR had previously held a Trial Co-ordinator role, so both had experience working directly in trials. During all focus group discussions, flexibility was employed to allow the conversation to flow naturally and participants to discuss what they felt was important. Following the focus group discussions, JT de-briefed with KR and ST to reflect on the conversations and any interpretations of what was said by participants. Neither co-facilitator felt that there were any leading questions or overt biases present in the discussions.

Data were pseudonymised, and participants were assigned an ID number. Pseudonymisation included the redaction of trial, drug and place names and any other potentially identifiable information.

### Analysis

**Fig 1:**
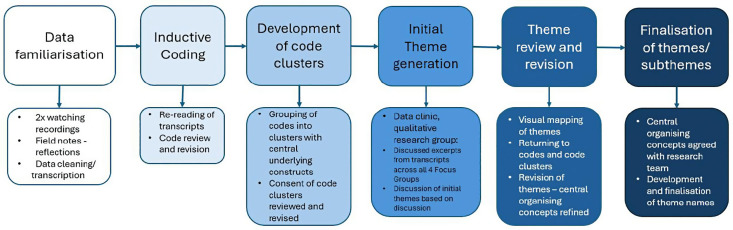
The stages of reflexive thematic analysis.

Data were analysed following the stages of Reflexive Thematic Analysis (RTA) [30] (Fig 1).

JT undertook data cleaning, intelligent verbatim transcription, data familiarisation and pseudonymisation simultaneously during two watch-throughs of the recordings for each focus group. Field notes were made as part of the familiarisation process.

Member checking and triangulation were not undertaken for this study as these principles assume that there is a fixed, objective ‘truth’ to be uncovered or validated, which is not consistent with the critical realist contextualist framework employed in this study [30,34]. However, to aid JT in the interpretation of the data the cleaned transcripts were circulated to participants for their comments and corrections, providing them with an opportunity to provide any additional thoughts or clarifications on comments that they made during the discussions. None of the participants returned any comments. One participant highlighted a typographical error, which was corrected.

Analytic rigour was ensured through functional transparency and reflexivity [35]. This involved maintaining an audit trail of the coding and theme generation process and engaging in reflexive dialogue to acknowledge how positionality shaped the analysis. This study achieves trustworthiness through theoretical coherence rather than consensus.

Coding was undertaken in NVivo v11.7 [36]. Coding was conducted inductively, focusing on meaning at the semantic level [30]. The coding process was iterative. Transcripts were read in full multiple times during the coding process. Codes were created, reviewed and revised several times. During coding JT returned to the data several times. Looking at the quotes grouped together under each code name, JT asked herself whether these groupings made sense, what was common or different about them and could this data be interpreted in another way or be placed within another code or multiple codes. JT also referred back to the topic guides and discussion that occurred around the chosen quotes to understand their context and determine whether the interpretation was reasonable and logical. When codes contained a lot of quotes in comparison with some other codes, JT considered whether the code was too broad and whether the data within it could be separated into more than one code or be better placed under another code. If a code contained less than three quotes, JT considered whether this data was significant enough to be considered its own code, whether this data would help with meeting the aim of the study and whether it could be merged with another code.

As previously described, the sample was expanded after the first two focus group discussions. The data generated from the second two focus groups was initially examined to identify data that fit within the previously generated code. During this process, JT noted that additional information and perspectives on safety reporting arose during this additional data collection that impacted her interpretation and understanding of the data from the previous two focus groups. Therefore, all transcripts were then re-read to identify any additional information that could be included in the existing codes and used to generate new codes.

A data clinic was held with members of the Qualitative Research Group at the Innovative CTU, whereby members were provided excerpts of transcripts from the Focus Group discussions to code and discuss. The discussion supported much of the interpretation that had already been undertaken by JT, though it also generated new insights and perspectives which were taken into account and considered during the next phase of the analysis. Following these discussions and coding of the data and the addition of the two new focus groups, it was felt that the data gathered generated sufficient information to meet the aim of the study.

The generation of code clusters followed a similar analytic process to the generation of initial codes. JT reviewed each cluster and sub-clusters to identify a central organising concept (shared meaning) that would capture the “essence” of a proposed theme [30]. During this process, JT started to write descriptions of the themes and what they contained, returning to the codes and data to understand how these all fit together. Where difficulties occurred in pulling ideas together, this indicated that there was perhaps a lack of central underlying concept and that the themes would need to be revised.

Generated themes and sub-themes were also visually mapped to show how they interlinked and establish whether there was any hierarchy (S4 Appendix). Initial themes were taken to the Qualitative Researcher Group and members of the research team and discussed. JT was asked questions about what the themes meant, what was contained within them and what they did to meet the aim of the study. In addition, for each iteration of the themes, JT followed the process of attempting to write what the theme was about and identify any parts that did not fit or made it feel like it was not “hanging together” with a single underlying concept. The data supporting the contents of the themes were examined and JT asked herself whether this fit and why/why not. Each new iteration of the themes was mapped visually to show the iterative process.

JT deduced that thematic sufficiency was reached when the theme generated could be considered as having an easily summarised central organising concept that felt coherent with what participants had said, which was supported by a diverse range of examples.

## Results

In total, 24 trials unit staff provided their written informed consent. One person was subsequently unable to attend focus group discussions for administrative reasons. Therefore, 23 people participated across four focus groups. Demographic information was obtained for 20/23 participants. Three participants did not provide demographic information despite multiple reminders. To protect the identities of participants, some demographic information, such as the list of trials units, is not reported in full.

Participants worked across 10 of 52 UK-based registered trials units, including the lead author’s host CTU [20]. Participants held a variety of roles, most operational, but some clinical. Participants had worked in trials for a mean of 13.3 years (SD 9.5), ranging from 4–40 years. The majority of participants worked in Oncology. The most common trial phases participants were involved in were phase II (N = 10) and phase III (N = 11) (Table 1).

**Table 1:** Participant demographic information (N = 20).

Demographic information		N (%)
***Roles***	(Senior) Trial Management	7 (35%)
	Pharmacovigilance	4 (20%)
	Clinical	4 (20%)
	Clinical Trial Monitors	2 (10%)
	Quality Assurance	2 (10%)
	Operations Manager	1 (5%)
Disease Area*	Oncology	10 (50%)
	Multiple disease areas	6 (30%)
	Infectious Diseases	3 (15%)
	Liver	2 (10%)
	Mental Health	2 (10%)
	Neurodegenerative	2 (10%)
	Acute trauma	1 (5%)
	Brain Injury	1 (5%)
	Cardiovascular	1 (5%)
	Long COVID	1 (5%)
	Gastroenterology	1 (5%)
	Geriatric	1 (5%)
	Gynaecology	1 (5%)
	Renal	1 (5%)
	Respiratory	1 (5%)
	Not specified	2 (10%)
Trial Phase*	Phase I	5 (25%)
	Phase II	10 (50%)
	Phase III	11 (55%)
	Phase IV	5 (25%)

*not mutually exclusive

**Fig 2:**
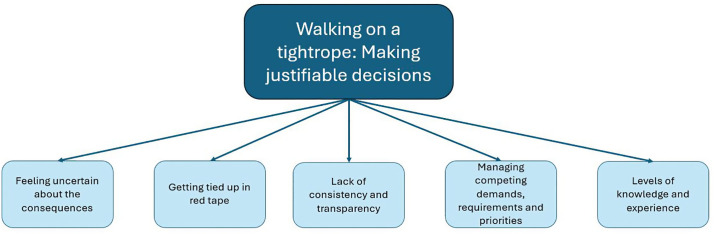
Thematic map.

Analysis led to the generation of a single over-arching theme*,* with five subthemes (Fig 2).

### Theme: “Walking on a tightrope: Making justifiable decisions”

The key theme identified as a barrier to efficient safety reporting was balancing risk. Trialists in the UK are expected to take risk-proportionate approaches to safety reporting [16]. However, decisions about safety reporting need to be justifiable. Participants appeared to be performing an ongoing tightrope walk, balancing risk-aversion, which can lead to over-reporting – taking up valuable time and resources, and putting patients at risk of harm through potential under-reporting.

“*Generally, you’ve got to balance what you need to collect in terms of safety reporting and in terms of what’s needed to monitor the safety of the drug*” (Participant 16, FG3)

Within this central theme, five sub-themes were identified that captured particular elements of this balancing act experienced by participants and some possible solutions to addressing inefficiencies and problems caused by these.

### Subtheme 1: Feeling uncertain about the consequences

Participants felt that despite efforts from regulators to encourage pragmatic, risk-proportionate approaches to safety reporting, uncertainty about the consequences of their decisions on drug profiles and patient safety and well-being created a hesitancy to implement them.

“…*It feels difficult and there is always that element of anxiety that you’ve got it wrong, or you’re not doing it right, or you’re not reporting it enough, or then conversely, you’re over-reporting, wasting everyone’s time, you’ve misunderstood…*” (Participant 20, FG4)

This leads to inefficient practices which may not be beneficial for understanding the safety profile of the drug or keeping patients safe, such as by obscuring important early safety signals.

*“The problem is that when you collect everything, you just get loads and loads of noise and you can miss the really important things”* (Participant 2, FG1)

Conversely, though capturing events considered high-risk based on their severity is efficient in terms of time spent on reporting, some participants felt that taking such an approach may have negative consequences for our understanding of patient quality-of-life when taking these IMPs, making the desire to report more events stronger.

*“…in some cases, only grade three to four or five events reported doesn’t really capture the patient experience of what being on those treatments is like. And we sort of just ignore all the grade one and twos even though that might be making people miserable.”* (Participant 4, FG2)

Overall, it appeared that the uncertainty that participants felt about the consequences of their decisions and what impact this could have on patient safety made it difficult for the participants to balance their decision-making in a way that aligned with risk-proportionality.

### Subtheme 2: Getting tied up in red-tape

Participants in this study felt that the bureaucratic requirements of IMP manufacturers and regulators were not always aligned with the limited time and resources available for academic trials. Moreover, some requirements, such as specific wording, are not really impacting patient safety.

*“I think probably Reference Safety Information management is a big part of it and I think a part that is overly, hugely bureaucratic and resource intensive unnecessarily for us as academic triallists. It’s hugely important for those manufacturing IMPs, but the kind of current process of managing that and the need to submit updates to substantial amendments and things like that is something that we were really hoping that in the update to the regulations we wouldn’t need to do.”* (Participant 17, FG3)

*“It is the regulators because we were told no, you have to have the exact words, the MHRA expect it...”* (Participant 3, FG1).

The needs to meet these requirements makes it challenging for academically-led trials to balance meeting their trials aims efficiently whilst maintaining patient safety.

*“We can be tied with so much red-tape that we’ve forgotten what we’re doing.”* (Participant 20, FG4)

Advocating for the implementation of risk-proportionate approaches by being clear about the aims of the trial and safety reporting, and “pushing-back” against seemingly unnecessary requirements is one way that this barrier to efficient safety reporting could be addressed.

*“I think if we’re coming across these things, …we should try and have a discussion with the regulator and say, can we agree a common ground that might make this more sensible. […] I think you can push back much harder; we have more power than we think.”* (Participant 2, FG1)

Regulatory requirements underpin the principles and processes of safety reporting. Whilst risk-proportionality is now legislatively required, participants universally felt that they were battling their way through this bureaucratic “red tape”, needing to complete seemingly arbitrary, unnecessary tasks that appear to work in direct opposition with this principle. This need to complete these tasks creates an inflexibility for trial staff which prevents balanced decision-making by shifting the focus of safety reporting away from its aims and more towards being a box-ticking exercise.

### *Subtheme 3: Managing competing demands, requirements and prioritie* *s*

Participants differed in their interpretation of risk and how it should be addressed across stakeholders. This created challenges for maintaining a risk-proportionate balance in reporting. Even for similar trials, these inconsistencies among stakeholders can lead to very different approaches to safety reporting and inconsistencies in what is needed from sites.

*“…You could be running an almost identical trial and be doing your safety reporting very differently. Somebody could be doing absolutely everything, and some people could be collecting and reporting very, very little...”* (Participant 18, FG3)

*“We don’t make it easy for the sites because the protocols are all different. What we report is all different, the forms we ask them to complete are different.”* (Participant 23, FG4)

The duplication of work that is produced as a result of these differences is a particular source of frustration and inefficiency.

“*…the EudraVigilance (EU) submissions portal is completely different in terms of what you enter on the portal compared to [Individual Case Safety Forms] (UK). It is double work”* (Participant 11, FG2)

Like battling through the red-tape, completing this additional work and addressing these competing priorities and interests distracts from the main aim of safety reporting. This can tip the balance towards inefficiency and risk-aversion as trial teams must work to the constraints of their most conservative stakeholders. Participants indicated that this balance could be redressed, particularly for more conservative stakeholders, by setting requirement parameters early on in the trial process and agreeing only to collect what is considered necessary.

*“It’s having those early discussions with [pharmaceutical companies] […] just to make sure that we’re collecting only what we really need to collect […] because sometimes they can be asking for far too much.”* (Participant 13, FG3)

### *Subtheme 4: Lack of clarity and transparenc* *y*

The balance of risk was also affected by a lack of clarity about how to make risk-proportionate decisions about safety reporting. Participants from clinical and operational backgrounds each felt that, despite efforts to encourage more pragmatic, risk-proportionate approaches to safety reporting, there was some ambiguity from the MHRA about how to effectively implement these. Valuable time and resources are taken up trying to interpret the requirements and address them appropriately.

*“But you know [the MHRA] give advice and they put out via blogs, and they put out via their website, but there’s no guidance that’s actually useful. I mean, I don’t know if anyone’s been involved in writing [Investigator Brochures], but that’s a particular horror show, in terms of MHRA. The [MHRA] give you strict guidance [on writing Investigator Brochures], you follow it, they knock it back and you end up just scratching your head and wondering what you can do given that you’ve followed everything to the letter.”* (Participant 16, FG3)

Many of the participants felt that improving the clarity of guidance from the MHRA could support the implementation of risk-adapted approaches.

*“…better interactions and feedback or more help from the MHRA if what you’re doing is appropriate might help us be more risk-based approach and more appropriate way ….”* (Participant 4, FG2)

These clarity and transparency issues affected decision making for sites as well as CTUs and Sponsors. Some participants experienced a lack of transparency about decision making that made it difficult to understand the justifications for decisions made at sites and determining whether they are risk-proportionate.

*“…but we don’t really actually get [site investigators] to write down anything about that decision making…. Or maybe they do in the medical notes, but they don’t on the [Case Report Forms]. And I wonder whether, overall, we may be massively under reporting SUSARs just because potentially there’s a tendency to just kind of tick that they’re unrelated…”* (Participant 18, FG3)

Participants felt that well-written trial documents, such as protocol, Reference Safety Information, Standard Operating Procedures and other working practice documentation could also support effective implementation of risk-based approaches by improving clarity.

*“…making sure that all aspects are clear, and everyone knows when things should be sent and what should be sent, when it should be sent and also the sorts of information as well that would need to be collected.”* (Participant 10, FG2)

### *Subtheme 5: Levels of knowledge and experienc* *e*

The ability to understand how to balance risk and implement risk-proportionate approaches to safety reporting appeared to be impacted by levels of experience and gaps in knowledge. Participants felt that this may affect confidence in their decision-making, impacting how well and efficiently decisions are made. This may lead to more conservative, less efficient approaches to safety reporting.

“*But I guess my experience and knowledge that I’ve got from it is that unless you’re regularly working with [Serious Adverse Events] (SAEs) on a trial, you’re not familiar with them and therefore you can be very uncomfortable, very unconfident in handling them”* (Participant 21, FG4)

Participants agreed that providing active, practical training and engaging in learning by sharing knowledge and lessons learnt from more experienced trial staff is advantageous. Developing this knowledge can increase confidence and address the issues with balanced decision making that can accompany this.

*“If you’ve got a really snappy training module that’s really easy to understand. You have a cute way of like presenting it or I think people like monkey-see-monkey-do, they like to see what form it is, they like to see how examples might be filled in. Things like that are really, really good.”* (Participant 20, FG4)

*“I think the shared learning across clinical sites with some of the trials that I’ve been on it I think is really valuable, right that’s a benefit that can be really, really valuable.”* (Participant 12, FG2)

## Discussion

The purpose of this study was to identify barriers and solutions to efficient safety reporting in clinical trials in the UK with a view to identifying recommendations to improve safety reporting practices. Findings contribute to the literature by providing a previously underexplored perspective of the issue of efficiency in safety reporting focusing on trial staff experiences from the UK, in the context of the changing regulatory landscape.

The central theme generated from the analysis was “Walking on a tightrope: Making justifiable decisions”. This theme captured a tension threaded throughout the focus group discussions, regarding finding the right balance of risk-proportionality to ensure that patient safety is maintained but staff time and resources are used effectively and efficiently. The reasons behind this experience were complex and multifaceted, reflected in the five subthemes: Feeling uncertain about the consequences; Getting tied up in red-tape; Managing competing demands, requirements and priorities; Lack of clarity and transparency; and Levels of knowledge and experience.

The findings demonstrate that, whilst reasonable steps have been taken to streamline safety reporting by regulatory bodies over the last decade [16] challenges persist in implementing them. However, participants felt some matters could be resolved through: further clear guidance from the regulators; increased transparency from regulators and site investigators about how decisions are made; and additional training and support to improve knowledge and confidence [12,25,26]

Furthermore, participants expressed that their decision-making impacted both the safety profiles of drugs under investigation and patient safety and well-being. This was an important factor in risk proportionality and was perceived as a difficult line to tread. The uncertainty that participants felt about the details of what should be collected appeared to be driven by a combination of bureaucracy, a lack of clarity, inconsistent requirements, demands and competing priorities.

In contrast to findings from Riordan and colleagues, which suggested that trial staff are frustrated by a lack of responsibility and under-playing of the importance of safety reporting by Principal Investigators [24], findings from this study mirror ongoing discussions which suggest that decision-making has been a continuing problem, because of concerns about the impact of over or under-reporting on drug safety profiles and patient well-being [7–9]. The new UK regulatory framework, which came into force in April 2026, complements changes introduced in ICH E6 (R3) in 2025, which focus on monitoring “critical-to-quality” factors only [37]. The regulatory update will address concerns regarding consequences of lack of clarity and transparency by eliminating previous ambiguities. For example, the new legislation now codifies the risk profile of a trial as being either: notification-only, full assessment or higher-risk. This clarification will enable triallists to determine the correct level of safety event information that needs to be collected and recorded in their trials [23].

However, participants in this present study felt that omitting the collection of information regarding low-grade toxicities could mean information about patients and their quality-of-life is being missed [11]. Such concerns are addressed in the new UK regulations and ICH E6 (R3) (section 7.1), which make clear that Patient Reported Outcome Measures (PROMs) should now be embedded within trial design to capture low-grade toxicities that may impact patient quality-of-life [37].

Additional similarities were found with previous findings on the pressures created by tasks viewed as superfluous and the duplication of reporting and information provision to regulators and stakeholders [14,38,39]. Like findings seen in Perez et al (2017), participants saw duplication as time-consuming and of limited value [14]. Under the new UK clinical trial regulations, duplication is reduced, as trial teams now only report SUSARs and Developmental Safety Update Reports (DSUR) to the MHRA. Additional reporting the REC is no longer required [23]. This change releases time and resources for staff to complete other trial activities.

Findings from this study reflected previous findings from the MHRA that there are staff with limited or no training working on non-commercial trials [27]. To address this lack of training and knowledge, participants suggested implementation of a range of training methods, including, practical training packages, creating a platform of collaboration through which knowledge and the lessons learnt from previous experiences can be shared. Mentorship and observational learning were also suggested by participants to have the potential to be effective methods for improving knowledge. As suggested in previous work, training aligned with the specific needs of the role/trial can be an effective way to support development and learning [25,26].

With changes to clinical trials regulations now in force for trials running in the UK, it remains to be seen to what extent the barriers to efficient and effective safety reporting identified in this study will be addressed. Based on suggestions made by participants this study, we make the following recommendations to support efficient, effective safety reporting, outlined in Box 1 below:

**Box 1. Recommendations**

- Trials staff involved in safety reporting should make use of information, resources and training available from the MHRA, HRA and ICH (S6 Appendix). [40–43]
- CTU staff involved in safety reporting to share knowledge, enabling the development of a network and/or platform through which experiences and knowledge can be readily shared.
- Through this knowledge sharing and collaboration, identify people with expertise across the community to guide and support the development of UK-wide mentoring schemes or easily-accessible, sharable web resources, such as case studies or document templates. Resources should be tailored to meet the needs of staff across roles across the trials community.

One strength of this study was that many participants had years of experience working across roles, trials and/or disease areas, meaning they could compare processes and highlight inconsistencies, list those identified between sponsors and across regulatory authorities. It was this breadth of experience that appeared to underpin this understanding and feeling of inconsistency experienced by the participants, which may not have been felt by people with less or narrower experience. This also enabled the participants to come up with practical and useful solutions to the barriers they identified.

This study also had some limitations. Interviews were conducted prior to the new regulations coming into effect. Some of the barriers to efficient safety reporting identified in this study may be resolved by the implementation of the new regulations and guidance, though this remains to be seen. The use of convenience sampling led to the majority of recruited participants primarily having experience in Oncology trials. The weighting towards oncology trials in this study means that some important contextual information about safety reporting burden in other disease areas may have been lost. To address this imbalance, during the discussions, JT tried to ensure that all voices were heard and that discussion focused not only on oncology specific issues, but issues that could be considered between different disease areas. However, it can be noted that this dominance of oncology trials is reflective of the wider clinical trials landscape, with cancer trials making up nearly a third of all studies, so this skew towards oncology trials would not be unexpected [43]. A further potential limitation of the convenience sampling method was the self-selection component. This may have impacted the types of barriers that were expressed and the degree to which the barrier or solution was deemed to be important or significant. This was mitigated through the use of the semi-structured topic guide to ensure that the conversation remained relevant and focused on answering the research question. JT managed the conversation by ensuring that all participants had the opportunity to respond to points raised by others in the group so that no single voice or idea dominated the conversation.

Despite the weighting towards experiences in oncology trials and focus on academically-led trials, there is a degree of transferability of findings from this study, as there are many aspects of safety reporting that are universal among CTIMP trials, such as the definitions of a Serious Adverse Event (SAE) and the reporting of SUSARs.

## Conclusions

Despite efforts to streamline safety reporting processes, problems persist with implementation of risk-proportionate approaches, due to concerns that triallists’ decisions will be deemed inadequate, with potential for negative impacts on patient safety and well-being. This study contributes to our understanding of how safety reporting is perceived by the trialists who complete this work, prior to the introduction of regulatory changes to clinical trials in the UK. These legislative changes will help to address some key barriers to efficient, effective safety reporting identified in this study. Emerging recommendations were: 1: For staff to make use of resources and training made available by MHRA, HRA and ICH. 2: CTU staff to share knowledge, enabling the development of a safety reporting network. 3: Identify safety reporting experts to guide and support the development of UK-wide mentoring schemes or easily-accessible, sharable web resources. Future work is needed to determine to what extent the barriers are addressed by changes to the UK regulatory framework.

## Supporting information

S1 Appendix SRQR checklist. (DOCX)

S2 Appendix Author’s information. (DOCX)

S3 Appendix Theme generation. (DOCX)

S4 Appendix Focus group topic guides. (DOCX)

S5 Appendix Focus group transcripts redacted. (DOCX)

S6 Appendix Resources and training for new CTR. (DOCX)
