# Supplementary material for: Walking the tightrope of justifiable decision‑making: An exploratory qualitative study identifying barriers and solutions to efficient safety reporting
Source: PLoS One. 2026 Jul 30;21(7):e0354806. doi: 10.1371/journal.pone.0354806 (PMC13422843; doi:10.1371/journal.pone.0354806)
Supplement: S2 Appendix — (DOCX) [file pone.0354806.s002.docx]

**Authors’ Information**

Macey Murray is a Senior Research Fellow in trial conduct methodology at the MRC Clinical Trials Unit specialising in the use of Health Systems Data in clinical trials. Macey had previously worked as a Trial Manager at the Comprehensive Clinical Trials Unit and Imperial College London; she therefore has prior experience of safety reporting processes from an operational perspective. She has also conducted research in post-marketing authorisation drug safety as a pharmacoepidemiologist and has studied methods of signal detection from European and global pharmacovigilance reports.

Annabelle South is a Principal Research Fellow who specialises in clinical trial communication and research impact. Annabelle has considerable experience in qualitative methodology and has worked in a clinical trials unit for more than 14 years but does not have experience of safety reporting processes.

Matthew Sydes has three decades of experience designing, running, analysing, interpreting and disseminating clinical trials. He is now Head of Data-Driven Clinical Trials at NHS England and holds an honorary chair at UCL where he was previously Professor of Clinical Trials and Methodology. He had a keen interest in methodological work to streamline the delivery of clinical trials.

Sharon Love is an experienced statistician currently working as an Associate Professor in trial conduct methodology at the MRC Clinical Trials Unit at UCL. Sharon specialises in clinical trial monitoring, a large component of which is the oversight of patient safety in clinical trials. Sharon also has previous experience as a statistician at Oxford Clinical Trials Research Unit and has therefore also had direct experience of safety reporting from an operational perspective.
