# Supplementary material for: Walking the tightrope of justifiable decision‑making: An exploratory qualitative study identifying barriers and solutions to efficient safety reporting
Source: PLoS One. 2026 Jul 30;21(7):e0354806. doi: 10.1371/journal.pone.0354806 (PMC13422843; doi:10.1371/journal.pone.0354806)

**Theme Development**

**Initial theme generation (August 2024)** Following the collection of data from all four focus group discussions. These were the initial thoughts from the clustering of the codes. These felt more like topics than themes, particularly given the amount of sub-themes that appeared within them.

**
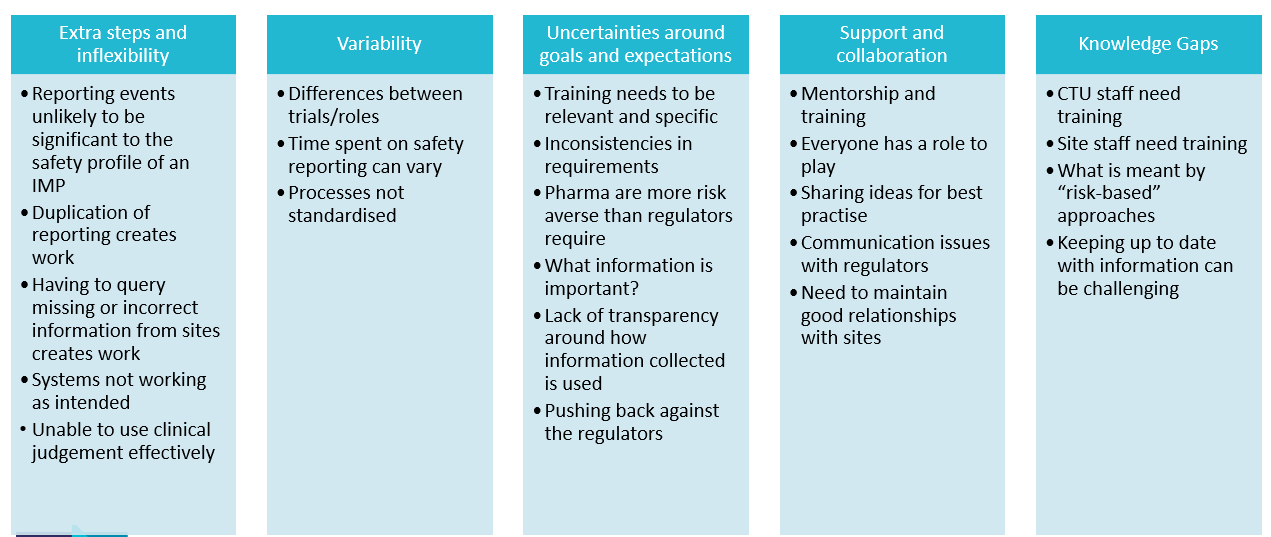
**

**First theme revision and transition to visualisation.** The number of sub-themes was reduced substantially to absorb some of the information and try to find the central concept within the themes.

**
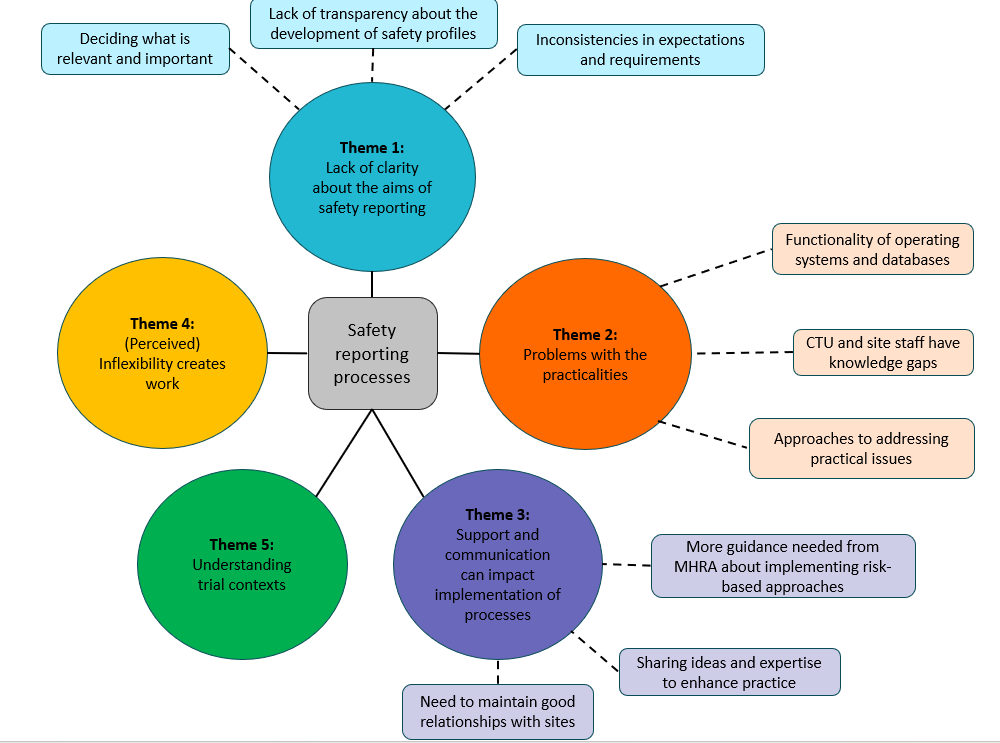
**

**Second revision of themes and visualisation**

**
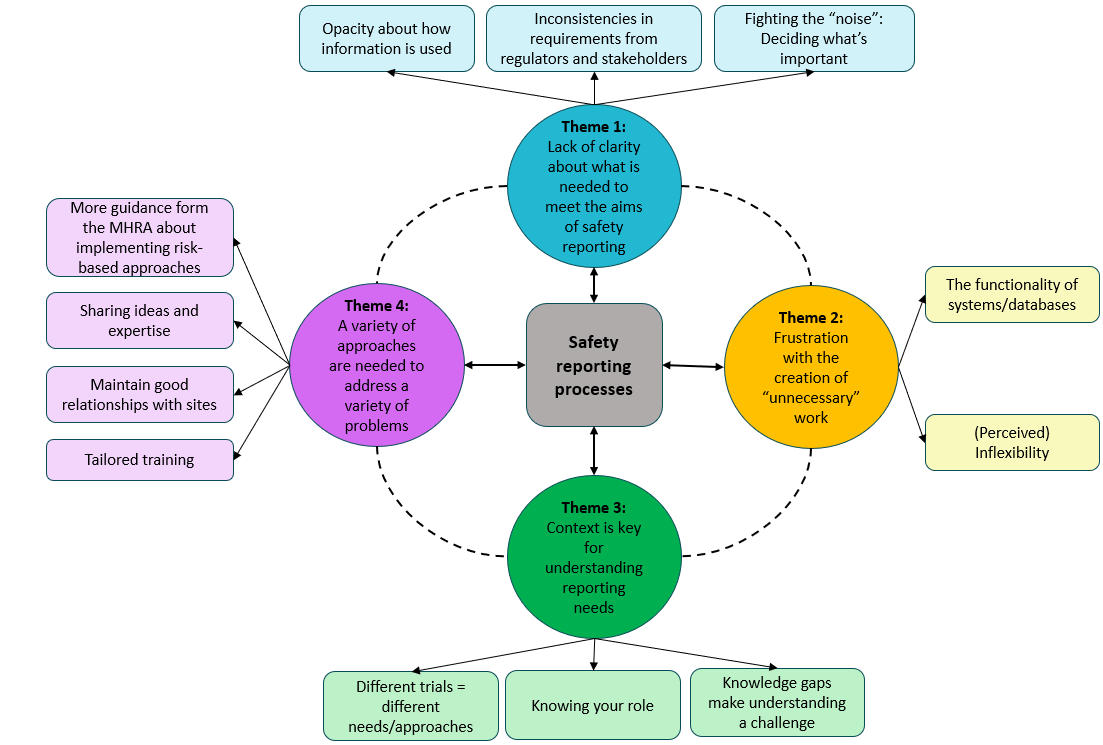
**

**Third revision of themes following discussion with Qualitative Research Group (September 2024).** Number of themes was reduced and revised from the above as central organising concepts were felt to be somewhat disparate after having had the discussions with the group.

**
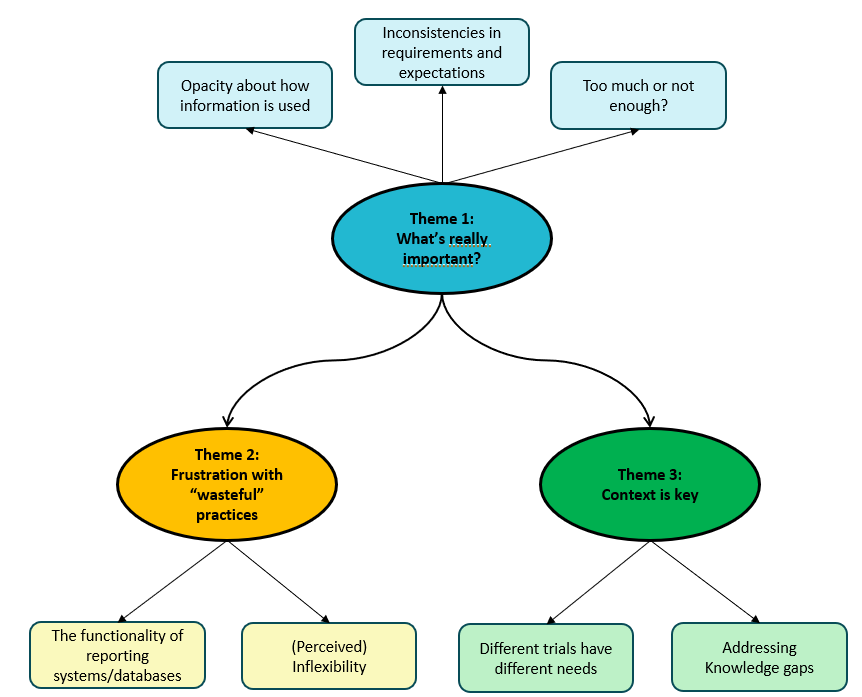
**

**Revision of themes (April 2025).** Data that had previously been part of sub-themes were considered as themes themselves. This idea was ultimately discarded as this seemed to be a backwards step, increasing the fragmentation of the data into topics, rather than themes. The first theme of decision making: Finding the right balance was kept and taken forward. The further 4 themes were re-examined and it was found that there were constructs within these which also related to the central organising concept of decision-making.

**
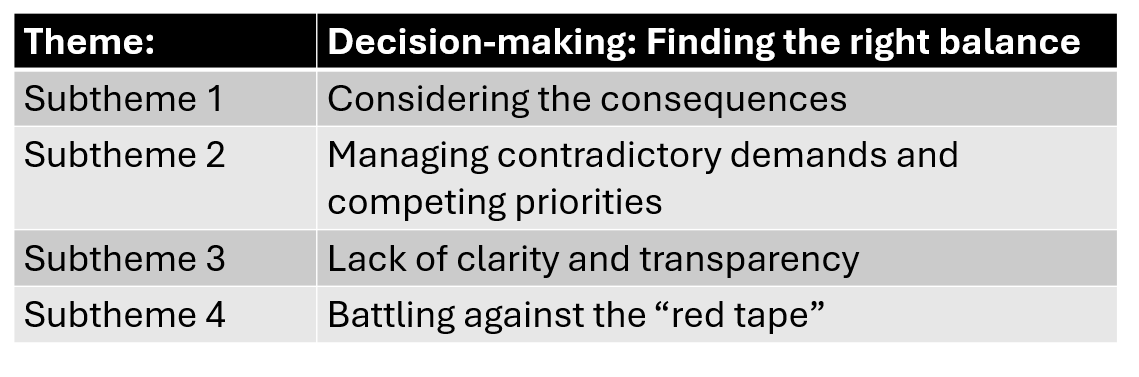
**

**
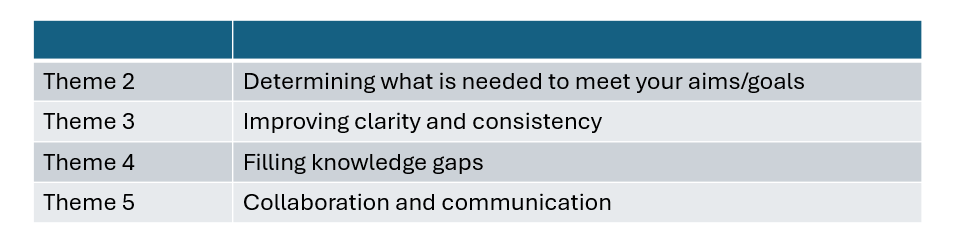
**

**Final revision of themes (June 2025).** The central organising concept felt clear and reflective of the data. JT returned to the codes and code clusters to get a sense that this felt like a reasonable interpretation of the data. Tightrope walking was chosen to represent the conceptualisation of this theme based on a participant saying: “It’s about **walking the fine line** between fully reporting the outcomes having a reportable trial that is responsible that provides you DMC with enough data that it can make correct decisions”. This made me think about balance and tightrope walking and felt like it represented what the participants were expressing during these discussions.


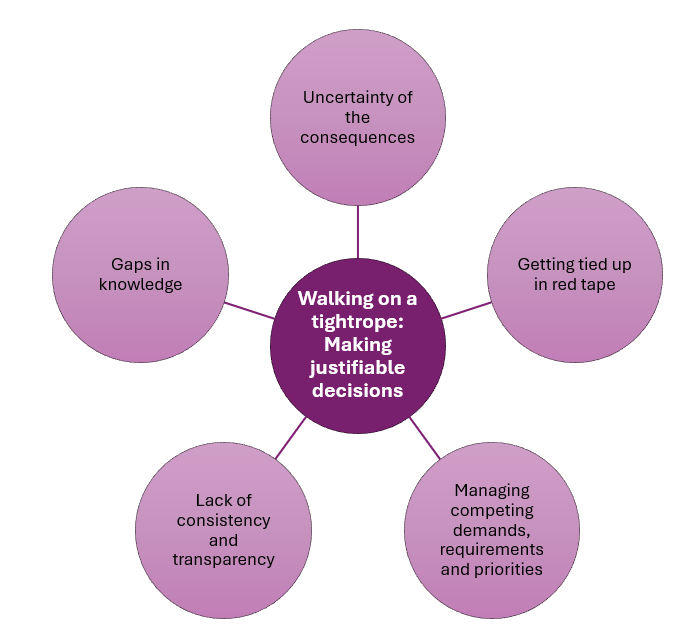

Supplement: S3 Appendix — (DOCX) [file pone.0354806.s003.docx]
