# Supplementary material for: Walking the tightrope of justifiable decision‑making: An exploratory qualitative study identifying barriers and solutions to efficient safety reporting
Source: PLoS One. 2026 Jul 30;21(7):e0354806. doi: 10.1371/journal.pone.0354806 (PMC13422843; doi:10.1371/journal.pone.0354806)
Supplement: S4 Appendix — (DOCX) [file pone.0354806.s004.docx]

**Beginning to identify the burdens of safety reporting for trials unit staff: an exploratory qualitative study – v1.0 Jun 2024**

| **Research Questions:**   1. *What do staff working at the Institute of Clinical Trials and Methodology (ICTM) think about how safety reporting processes in clinical trials currently operate?* 2. *How do trial staff think that safety reporting processes can be improved?* |
| --- |

**Focus Group Topic Guide**

**Defining “safety reporting” (15 mins approx.)**

- What is “pharmacovigilance” and how would you define it?
- What is “safety reporting” in the context of clinical trials, how would you define it?
  - Are pharmacovigilance and safety reporting the same thing?
- What is the role of safety reporting in the conduct of clinical trials?
- What are the main tasks or processes that make up safety reporting?
  - Do these vary throughout a trial
    - How?
    - Why/why not?
- Are there any ancillary tasks/processes related to safety reporting but are not considered part of the central, mandatory processes?
  - what are they?
  - What distinguishes these tasks from the main tasks/processes involved in safety reporting?
- Do you think that all of these tasks are needed?
  - Why/why not?
- *Are there similarities and differences are there in understand of what safety reporting is between:*
  - - *Trial design (and CTIMP, non-CTIMP, ATIMP etc)*
    - *Trial phase*
    - *Patient group/disease area*
    - *Where the trial is within its lifecycle*
  - *If so, what are these differences/similarities?*
    - *Where and when do they occur?*
    - *Why do they exist?*
      - *Should they exist*
        - *Why/why not?*

**Safety reporting as part of the job/role of clinical trials unit staff (15 mins approx.)**

- What role does safety reporting play in a typical working week?
  - How much time on average would you say you spend on safety reporting tasks?
  - Do you think there is a way that time spent on these tasks could be measured meaningfully?
    - What would that be?
    - How would it work?
    - Would this be beneficial for workload planning?
      - How?
      - Why/why not?
- Where does safety reporting sit within the hierarchy of your work tasks/activities?
  - Why?
- How do you manage safety reporting within your workload?
  - Are there challenges managing safety reporting within workload?
    - If so, what are they?
    - What makes them challenging?
- Does this aspect of your job affect your well-being or performance at work?
  - How?
  - Why/why not?
    - In what ways?
- Are there similarities or differences are there between:
  - Staff members and their roles within the trial team
  - The disease area or patient group
  - The design of the trial (and whether it is CTIMP, non-CTIMP etc)
  - The phase of the trial
  - Where the trial is within its lifecycle
    - If so, what are they?
    - If not, why not?

**Relationship between patient safety and safety reporting (10 mins approx.)**

- How do you perceive the relationship between patient safety the safety reporting tasks that you need to do as part of your role within the trial team?
  - Is this aspect of safety reporting important to you?
    - Why/why not?
    - What could change your opinion on this?
- What do you feel the impact is between the safety work you do and how safe patients are kept?
  - Could this be better/worse?
    - What would change this?
    - How could you change this?
- Do you think your feelings about the relationship between safety reporting and patient safety would differ or be the same between:
  - Trial design
  - Trial phase
  - Patient group
  - Where the trial is within its lifecycle

**Regulatory guidelines and CTU practices (10 mins approx.)**

- What are your thoughts on the way that safety events are reported to regulators?
  - What documentation is needed?
  - What are the processes?
    - Are they efficient?
      - Why/why not
    - Are they necessary?
      - Why/why not
- There are many regulators (such as the MHRA in the UK) that now suggest that trials take “risk-based approaches” to safety reporting
  - Does this happen in your trials?
    - To what extent?
  - What are the facilitators and barriers to this risk-based approach?
- If a risk-based approach is not taken
  - Why not?
  - What approaches are taken instead?
  - Would a risk-based approach be preferred if it were feasible?
    - Why/why not?

**General opinions on safety reporting processes (15 mins approx.)**

- What impact do you think safety reporting processes in their current form have on the development and conduct of clinical trials?
- Do you think that safety reporting processes in their current form are as efficient as they could be?
  - What aspects of safety reporting do you consider to be the most efficient/worthwhile?
  - why?
  - Are these benefits the same for:
    - Trial staff
    - Clinical teams
    - Patients
  - To what extent are they similar or different and more or less efficient/worthwhile?
- What similarities and differences are there in the benefits of safety reporting between:
  - The disease area or patient group
  - The design of the trial (and whether it is CTIMP, non-CTIMP etc)
  - The phase of the trial
  - Where the trial is within its lifecycle

**Improving safety reporting processes (15 mins approx.)**

- What areas of safety reporting in clinical trials do you think could be improved?
  - To what extent would changes to safety reporting processes benefit
    - Clinical teams
    - Trial staff
    - Patients?
- How do you think safety processes could be improved/streamlined whilst maintaining an acceptable level of patient safety?
- How might these suggested improvements be facilitated?
- What barriers might exist for implementing these suggestions
  - How might they be mitigated?
- Might there be similarities or differences between:
  - The disease area or patient group
  - The design of the trial (and whether it is CTIMP, non-CTIMP etc)
  - The phase of the trial
  - Where the trial is within its lifecycle
  - Who benefits most from proposed changes (clinical teams, trial teams and/or patients)

**Beginning to identify the burdens of safety reporting for trials unit staff: an exploratory qualitative study – v2.0 Dec 2024**

**Focus Group Topic Guide**

**Introductory questions (10 mins approx.)**

- What do you understand by the term “safety reporting” in the context of CTIMP clinical trials?
- What do you understand by the term “pharmacovigilance” in the context of clinical trials?
- What do you think are the main tasks and processes that constitute safety reporting/pharmacovigilance?

**General questions (15 mins approx.)**

- What impact do you think safety reporting processes for CTIMPs in their current form have on the development and conduct of clinical trials?
- What works well about safety reporting processes for CTIMPs as they operate now?
- What could be improved – where are their inefficiencies and issues with safety reporting in its current form?

**Regulatory and pharmaceutical guidelines (20 mins approx.)**

- What do you think of the processes that the MHRA and other regulators have in place to report safety events?
  - What works well?
  - What could be improved
    - How and why?
- Is it clear to you how the collected information is used by regulators and pharmaceutical companies and how it impacts the safety profile of the IMP?
  - Could changes to this improve safety reporting processes?
  - How?
- What are your opinions on the online portals and reporting processes for different regulators/stakeholders?
  - What works well?
  - What could be improved?
    - How and why?

*The MHRA and other regulators are attempting to streamline safety reporting processes through the introduction of “risk-adapted” approaches to safety reporting in CTIMP trials*.

- What do you understand by “Risk-adapted” approaches to safety reporting?
  - What is clear/ unclear to you about what this means?
    - What makes these aspects clear/unclear?
- How have “risk-adapted” approaches been implemented in your trials/unit?
  - What are the similarities/differences to previous approaches to safety reporting?
- How do you use Risk-adapted approaches to determine what and how much information needs to be collected?
  - How effective are the methods used?
- Are there similarities/differences in how safety reporting processes are implemented depending on the trial phase or disease area?
  - What are they?
  - How do they impact safety reporting processes?
- Do the needs of pharmaceutical partners affect the implementation of risk-adapted approaches to safety reporting?
  - In what ways?
  - Are they consistent with the needs of the regulators?
- What are the limitations/barriers to implementation of “risk-adapted” approaches to safety reporting?
- What could be done to improve the implementation of “risk-adapted” approaches to safety reporting?
  - CTU/local level
  - Regulatory level
    - How?

**Training Needs (25 mins approx.)**

*To implement safety reporting processes effectively, staff need to be adequately.*

- Can you explain the training you undertake in your CTU to learn how to complete safety reporting processes?
  - Are the similarities or differences between CTUs and trials?
    - What are the impacts of these differences/similarities on safety reporting processes?
- How effective do you think current training processes are for CTU staff?
  - What improvements could be made?
  - How?
  - What impact would these changes have on safety reporting processes?
- How effective do you think current training processes are for investigator/site staff?
  - What improvements could be made?
  - How?
  - What impact would these changes have on safety reporting processes?

**Any other areas of safety reporting (10 mins approx.).**

- Are there other areas of safety reporting in clinical trials do you think could be improved?
  - What are they?
  - How would change improve processes?
- What are the barriers/facilitators to change and improvement?
  - How can barriers be overcome?
