# Supplementary material for: Walking the tightrope of justifiable decision‑making: An exploratory qualitative study identifying barriers and solutions to efficient safety reporting
Source: PLoS One. 2026 Jul 30;21(7):e0354806. doi: 10.1371/journal.pone.0354806 (PMC13422843; doi:10.1371/journal.pone.0354806)
Supplement: S5 Appendix — (DOCX) [file pone.0354806.s005.docx]

**Transcript Focus Group 1: 01-Jul-24**

1 July 2024, 10:04am


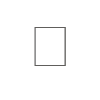
 **Thompson, Jemima** started transcription

**Thompson, Jemima** 0:03
I want to start, so we're gonna be kind of doing this in a few sections and I want to start by just asking you all, basically, what do you understand by safety reporting and pharmacovigilance in the context of clinical trials?

**Participant SR001** 0:25
Um for me, I would say it's reporting serious adverse events from the site and then reporting the SUSARS to the MHR- the regulatory authorities such as the MHRA, the uh, EMA and also the ethics committees.
It's like- in a nutshell.

**Thompson, Jemima** 0:47
Yeah, it's nice little summary. Anyone have anything to add to that?

**Participant SR002** 0:56
I mean, it's part of our crucial obligation in clinical trials.
Um, [inaudible] part of GCP.
Inherent Safety reporting is making sure that, um the sites have been properly trained in in what is required to be reported, and it doesn't just include SAE’s and so forth, includes different grades of adverse event and the pathway for reporting those. So that has to obviously be all laid out very clearly in the protocol and in the manual of instructions and everyone needs to be trained in what the reference safety information is as well, because that's your, that's your most important, not just your sort of your grading tables and being aware of those, but also what is your reference safety information for all your IMPs and your IMPs have to be defined in in each of the trials quite carefully.

**Thompson, Jemima** 1:59
Thank you [SR002].
Anyone else anything to add at this point?

**Participant SR003** 2:08
No, I’ve got nothing to add to those. Think [SR002] covered it pretty well.

**Thompson, Jemima** 2:08
No. Yeah. And so yeah, yeah, it's really nice summary. And I suppose, what do you all think are kind of some of the main tasks and processes that are involved in safety reporting? I know you've kind of mentioned a couple, but kind of what are those, how do they fit? Where do they happen?
And I guess kind of how important are they? Are there any tasks that are maybe we have to do that are less important? And yeah, is there a hierarchy?
Yes.

**Participant SR002** 2:45
I think it depends on whether we're sponsor for a trial or somebody else is a sponsor, so there might be different responsibilities in regards to our role in, in, in safety reporting depending on that crucial difference. And the reason I guess I highlight that is because I wear a different hat in terms of my responsibilities in reviewing you know SAEs and so forth across two different trials.

So, one example is where we are not sponsor, we're a sort of conduit, if you like, between our sites in a number of countries and the sponsor for the [trial names] trials where the sponsor is University of [place name] where I'm a medical officer. So it's my responsibility to make sure that, you know, to train the sites, but also to make sure that I review all SAEs, and make sure that they the story is as complete as possible in terms of- and then it's and then that is all that all goes through red cap and is reviewed by the medical monitor who has the overarching responsibility for yo- you know, what happens next in terms of whether it meets the criteria for a SUSAR, all those sorts of things.

So, I've got this sort of intermediary role on one set of trials and then a definitive role on, for example, [two trial names], where I with one of my colleagues [name], review all of the notable events, SAEs, grade three and four clinical events. you know, all the things that are required to be reported we [inaudible] those protocols, and we also have to write a summary of, you know, a clinical reviewer summary, which is all completed on Open Clinica and we you know, we look at the MedDRA coding and several which is actually automatic in is automated in Open Clinica and just to make sure that.

So, I think the crucial thing is it can be different things depending on whether or not we're sponsor, which is actually quite important because many of the trials in, in our unit work in slightly different ways. We don't, we're not always sponsor, so thanks.

**Thompson, Jemima** 5:04
Thank you, [SR002]. I think it's a really important point about the differences between whether you're sponsor or not. I don't know if anyone else has anything.
That it's different or similar.

**Participant SR003** 5:18
Well, I know I've worked on trials where you know it depends on, as well which grading you're using, because some trials only use grades one and two. Some yeah, and others just go from three and four. So, it depends on the level of safety reporting you're doing and you're relying on sites to report them to you, which you then check at monitoring, or get back to them.

**Participant SR001** 5:44
Yeah, I would say 'cause. I'm from the sponsor perspective usually.
I think site report safety reporting and sponsor safety reporting is quite different because of the different things that they do. So, for example the site report everything, so adverse events, serious adverse events, adverse events of special interests and they have, they can have different timelines, they don't do the exp- for us, they don't do the expectedness assessment, we do that, but they do the causality assessment. So, they have different, um priorities. So, and then it comes to us and we have to have general oversight over everything. And then we have to- we're responsible for the reporting to the competent authorities. So yeah, there's different timelines, different roles. So, it is quite different from sponsor to site, definitely. And yeah, from trial to trial. The- yeah, the- what is significant. What- what is considered, kind of exempt from reporting is different for each trial because as you say, you can have anticipated events from dif- if for example, you might only want to report grades four and above to, that's like uh, I guess, um, unanticipated.

So, it really depends from trial to trial. It's quite each protocol is different and you have to make sure that you know each protocol. So, the safety reporting can be different per protocol really.

**Thompson, Jemima** 7:15
Yeah, it's definitely been my experience as well. I just wonder from listening to you all speaking about that, you've talked about the differences between the sponsors and the sites and things. And I just wanted to get your perspectives on that kind of team element and how you all work together and who's involved and it's, you know, it doesn't, it seems like it's not just a one man- one person operation.
How does- how do you find that that works? Those kinds of relationships with the sites and with other members of your team?

**Participant SR001** 7:49
Umm, for us, I'd so that we have a PV team of three people. So, I know some places have smaller or larger teams. But we work very closely with the trial managers and then the trial managers kind of they work with the sites. So we- the PV team, we don't go directly to the sites, we kind of, if we did potentially have queries on a form, we would go through the trial manager. So, we have this kind of site report to trial team then trial team can converse with PV. That's kind of how we- and we want to keep a good we always want to keep a good relationship with the trial team. We want to be there to help them, not to tell them what to do. So we want to keep this kind of open conversation that it's OK to ask questions. It's we're not, no kind of judgement if you don't know anything, we're here to help instead of here to just tell you what to do. And I think that definitely benefits because it means people are more engaged and they're more willing, they're more likely to ask questions if they have them.

I think that's important.

**Thompson, Jemima** 8:56
Hmm. Yes, [SR002].

**Participant SR002**
And we have a very good working relationship with all of our sites, we’ve worked with many of them for decades actually, and I think maybe some of the differences that, certainly in the trial [name] and [trial name], I am CI of one of those trials and clinical reviewer, one of the clinical viewers for both, we have a very open discussion. about adverse events and so forth through we obviously use Galaxkey and to really and our guidance is always you know if you're not sure you know reach out and we can we can talk about this and we frequently will hop on a call to talk through if people are not quite sure, especially if it's something quite unusual.

One of the examples where I think that we really had to work together to help the sides was, a um, a unfortunate participant, who developed a very nasty cancer at an early age and was having repeated admissions to hospital for chemotherapy and so obviously the reporting requirements is that every admission, even a planned admission, is a serious adverse event. So, that's kind of different in how we used to do things from a few years ago. So you know, having that discussion on you know how to handle that and the enormous amount of con meds every time, and so forth and we, we, we, we actually talk that through and would try to rationalise it so it wasn't just such a huge burden every single time they were reporting this planned admission as an SAE, which is a requirement, so that was really because we've not faced that situation in the past, and so we were sort of working together to minimise the amount of work it was for the sites and that was that was really important and helpful. So I think that's just an exemplar of, you may not know all the answers, but sometimes you, you know, you need to just work through and think about what it, what's really involved from the site's perspective, not just say, oh, well, you know, you have to put in all the con meds, every single time and blah, you know, just, you know, and everyone's just getting like, they insist taking hours and hours of work and you know, so. So, I think that in building on the relationship and rapport and being flexible to new situations where you're having to find, you know, work arounds, you know, meet all the requirements but also be cognizant of what the sites might be facing in terms of the reporting. So, thanks.

**Thompson, Jemima** 11:45
Thanks [SR002]. [SR003], did you have anything to add to that?

**Participant SR003** 11:52
Not really, no. I mean, we get all ours, I mean the sites enter the data directly into Open Clinica database and then we get reports off there, of AE's that need to be followed up. If there's queries, we enter queries and stuff and then any SAEs that are alerted to us come as an automatic alert. So, we're aware of them almost immediately, so we can then report on.

**Thompson, Jemima** 12:19
Is that? Something that's different to kind of previous, I suppose like iterations and trials where because with Open Clinica it's automatic right and it gives you like a flag.

**Participant SR003** 12:33
Yeah.

**Thompson, Jemima** 12:34
How do you think? Yeah, I just wonder.

**Participant SR003** 12:36
Yeah. I mean, previously you'd have to wait for them to send you in the form or fax you in a form or scan it in or whatever by. But now they can do it straight into the database and then it alerts us the next morning and it's ready for us then to activate. Yeah, to report on if necessary or send to clinical reviewers.

**Thompson, Jemima** 12:56
Hmm, and I just wonder, listening to you all talk as well about, you know some of these different tasks and the relationships with sites and things, are there any things that you're having to do any of these tasks and processes that you think could maybe be more efficient or that you kinda think I don't know why they're there in the way that they are, or some stuff that you think is like this is amazing, I'm so glad that we did this this way.

**Participant SR002** 13:27
I’m very vocal, aren't I? So, look, I think one of the things that's been really good in the [title] networks have been a long-standing network we've worked with for a long time and we're one of the founding parents, if you like of that network. But we for a long time were wedded the division of AIDS toxicity tables, which, to be honest are a complete pain in the neck because you, you know, you have to, you can put a search engine on the PDF, but, it's not a, you know, there's no web that you can just search on that. So the [title] network has moved to using the CTCAE Toxicity tables, which are absolutely fabulous because you just sort of start typing in and then it and it comes up with, you know, what, it thinks that you're going to want to call the adverse event term if you like, and it even does grading and it tells you about the MEDdra Codings.

S,o it's kind of like so much more user friendly than the division of AIDs toxicity tables, and I think that that move away from the DAIDs Tox tables, is it, it would be something that I think would be helpful unit wide and because the CTCAE tables for the sites for us are so just like it's just a world of difference instead of being a world of pain, it's just a world of easy-peasy. So, I think that sort of, you know change in the tox tables maybe more university across the unit would be, would be good. It's my, it's my opinion.

**Thompson, Jemima** 15:05
Thank you, [SR002].

**Participant SR001** 15:17
What was the question? It was it.

**Thompson, Jemima** 15:20
So kind of asking you about, basically, some of these tasks and processes are that you have to do. Are they all needed? Are there things that are great? Are there things that could be better? Just kind of off the top of your head.

**Participant SR001** 15:37
I mean, for me, from a personal perspective, because I I'm the one who does the SUSAR reporting to the competent authorities. So, for me it would be quite after Brexit things changed and you have to- if you have a trial that's both within the UK and the EU, you have to report the same SUSAR twice. Essentially, you have to do it on EudraVigilance and you have to do it on ICSR submissions and it's a real pain because it means double the amount of work and it's - the systems don't really talk to each other, so you can download an XML, you can't use that same XML on the different systems, even though they have very, very similar fields. So if they could change that to make it a bit more- So, for example, if you could just upload an XML and not even get everything in the exact right place, but at least some of the information in the in the right place, that would just be better than having you have to complete them from scratch and it can also, it's double the potential for human error as well because you are just punching numbers in really. So it's I think that for me that would definitely make a difference if there's some kind of, unity I don't know to when you can, for site trials that have SUSARS that you need to report on EudraVigilance and ICSR submissions

**Thompson, Jemima** 16:57
Thank you, [SR001]. [SR003], did you have anything to add?

**Participant SR003**
No, it's ah- I mean we have to refer our SUSARS, or potential SUSARS to someone different because we- as a blinded trial, to us, they're just potential. So, we don't know the full outcome to the finally, if it's been reported or not, so.
That’s um, one step that's sort of an extra step because you have to, can't report them ourselves.

**Thompson, Jemima** 17:24
Yeah. Yeah, and I suppose, I mean, is there a way that?
Yeah, I suppose. What's that like? You're kind of handing it over to some someone else to deal with.

**Participant SR003** 17:36
Yes. Yeah. So, they have to do the reporting for us and then there's always the questions that come back from them. Oh, have you got this bit? Have you got that bit and?

**Thompson, Jemima** 17:47
And so is it that the sites directly communicate that to you, and then you pass it to the- your kind of PV person?

**Participant SR003** 17:52
Yeah. Yes, depending on whether it's related or not as to whether it's a potential SUSAR.

**Thompson, Jemima** 18:00
Hmm. And do you think that that's like the most efficient way of doing that?
In your opinion?

**Participant SR003** 18:07
I mean, it used to be that you reported them all, whether they were.
Even if they were still blinded, you'd report the incidents, but now I mean it. Now it's just they just want to know, ones that want them unblinded. If you want to- like the authorities. So yeah, that's sort of an extra step for blinded trials.

**Thompson, Jemima** 18:29
Yeah. So there, there, there seem to be a difference depending on whether you're blinded or unblinded. As to the right, which makes sense.

**Participant SR003** 18:34
Yeah.

**Thompson, Jemima** 18:37
I suppose I'm kind of. I'm looking at the time, so I think unless anyone's got anything else they want to add about, like what safety reporting is and what that looks like for them.
OK, so I'm just gonna move on to. So we've kind of touched a bit on this already. [SR001], I know you've mentioned about the regulations, but something that we know has been happening kind of you know all over the place and in the UK in particular is the y-, the regulations and obviously that we're having to follow those when we're doing our safety reporting. And there's been this shift towards taking these more risk-based approaches to safety reporting. And I just kind of just kind of put a little definition here. And so the events that are reported is decided based on the overall risk profile and of the interventions under investigation. And I just wanted to get your views on are the trials you're working on taking this approach if they are, how successful is that? To what extent is it happening all of this kind of stuff? So, I'll open that out to you.

**Participant SR003** 19:56
It depends on the phase of the trial as well, because when you're working on phase one or two like the vaccine trials I used to work on, you have to report everything basically, all Grade 1 twos, threes and fours, whereas other studies now you know sort of.

**Participant SR003** 20:10
They do- I know some of their sort of phase four of you only report, sort of grades threes or fours.

**Thompson, Jemima** 20:24
Yes.

**Participant SR002** 20:26
Yeah, and I think we do really try and take a risk based approach.
And actually, even, you know, during the COVID pandemic, we did actually take quite a lot of IMPs where we had a little bit of phase one data and we just took them straight into sort of phase three, right, because it was an emergency situation and we did actually minimise the number of the amount of AE reporting that was required.
I think most of it we in in terms of the manufacturers of the IMPs we fought very hard to minimise you know collecting all sort of those low grade adverse events and in the end we I think that we had to do a couple of days or a week of all the adverse events and then focusing on grade three and four clinical adverse events you know things that really matter and the SAEs. So, in an emergency situation you can actually, you know, do things slightly differently. It was a risk-based approach. I mean, these were mostly monoclonal antibodies, which we knew quite a lot about from before, obviously they were COVID focused, but you know nothing, nothing terribly, you know, we didn't expect anything to emerge that was very different from other trials and other infections.
So I think that, risk based approach can is important depending on as [SR003] said, the phase of the trial, the setting of the trial. Is it an emergency setting or is it you know is it peacetime or is it wartime? I mean, you know, and I say that's a way of describing it and also just trying to really think about.

What it is that you're hoping - What is it? What you're really trying to get from your safety reporting because, you know, the problem is that when you collect everything, you just get loads and loads of noise and it can, you can, miss the really important things because there's so much noise, you know, grade one noise. So I guess you know that that is really something I think that people strive to try and minimise the noise so that you really focus on what's really an important safety signal that you don't want to miss in amongst all that, you know it Grade 1, itchy nose. You know, I don't know. Do you know, do you know, you know, how you can just get overwhelming?

So I mean, that's where all of the documents you know, like the safety management plan and all of that side of things, is important in really trying to drill down on on
what you're trying to achieve and even sort of push back against regulators saying, oh, you have to do this and this because we, we've done that very successfully. Sorry a bit long winded in trying to explain but you know.

**Thompson, Jemima** 23:19
Hmm. No, not at all. It's really nice to have these nice detailed answers. This is what I want. I want to know kind of you know what you’re all experiencing. [SR001], were you gonna say something? Yeah.

**Participant SR001** 23:31
Yeah, I was gonna say I would definitely agree with that in the sense of we've found a lot- so for example, we had- we have- because I work only on cancer trials, we do get that the a lot of SAEs and sometimes you do- So, we have a trial where patients standard of care, they're going to get chemotherapy before they get the IMP. So, they do get a lot of infections, they get neutropenia, and we've noticed that one of our trials we just get so many SAEs of neutropenia, neutropenia and it is just it's, it's a lot of work that everyone, the sites have a lot of things to report. And then because it's it's not I think it's a phase, it's a phase two. So, a lot of them are unexpected. So, it's a lot of SUSARS. So we have to report them, we have to probe out with queries. It's just so much work for everyone and it's an international trial, so you've got the double submission.
What we're thinking of doing now is updating the protocol, so we only want to collect the grade, well we're going to, we haven't got approved for it yet. So, we're going to go for submission for approval, but we want to collect only the ones that are grade four and above because it will just reduce the noise. So we can see, as you say, we can reduce the noise. So, we can see the important things more than just having the burdensome amount of SUSARS that we have at the moment.
The things that we know are most likely down to the chemotherapy. So it's, yeah, it's I think it is good to take every trial and see what you.

So, with the risk assessments, what are you going to expect and how can we kind of, focus what we are collecting. So, we're collecting the information. That's the most important and yeah. And yeah, we have pushed back onto the regulatory authorities as well and they have agreed with more risk-based approaches and it's not just like a blanket for everything, so that's quite good as well, actually, yeah, we've seen that as well.

**Thompson, Jemima** 25:36
Hmm. Thank you. And [SR003], did you have anything to add?

**Participant SR003** 25:44
Yeah, nothing more to add.

**Thompson, Jemima** 25:46
And I suppose listening to you talk about these approaches and kind of that you think it's helpful and you've pushed back against the regulators. What I suppose are the barriers, potential barriers to enacting these more risk-based approaches. Have you encountered any or kind of what's that been like pushing back against the regulators?

**Participant SR001** 26:14
We have had a certain one where we have to trial- so one trial, we're not the sponsor and then we have like an add on trial where we are the sponsor and we wanted to align our protocol with theirs, but the MHRA didn't approve it, so we had, so it's different. So, it's I think if you have two different sponsors, they don't really look at the, they look at it kind of separately instead of as like a whole because even though they are the same patients, it's just an add on trial where they get an extra antibiotic.

It's they're still seeing it as kind of two completely separate, completely separate people, which is good and bad in the sense of it's, yeah, I I don't know. You know, if that makes sense.

**Thompson, Jemima** 27:01
That does make sense. I can see what you're saying. [SR002].

**Participant SR002** 27:08
Yeah, I think it depends on the regulator.
The toughest regulator by far and away is the FDA. By far and away and what we've come across is that things that were quite easy and straightforward during the first kind of two years of the COVID pandemic have suddenly become just much, much more arduous. Now we're in a different phase of that pandemic, right? And we've faced problems with different departments of the FDA reviewing the two different trials, and they’re sort of coming up with different questions. So, there's this sort of complete not sort of joined up. One group are reviewing one thing, one group and I think that's probably also true, for example of the EMA as well. You've come across different depart- one department's kind of very reasonable and then another department almost, you know, and this is a problem in some of this sort of adaptive trials as well where you've got different bit sections of the the regulations reviewing, you know, different trials within the platform and coming up with different advice. So that's one challenge. The regulators not sort of being joined up within themselves, there’s not internal consistency.

And the second big challenge is that Pharmaceutical industry for the most part are incredibly risk averse. S,o whenever you're doing a trial that's about, you know it's a registrational trial, honestly, they just get sillier and sillier, right? You know, they, so please don't quote me, but, but, you know, some pharma companies are more reasonable than others. It depends on their level of experience and confidence. And others are just, especially if they're following into a newer area within their pharma, you know, say they've bought a drug from another pharma company, they're just not so sure.
And then it just becomes, you know, very pedantic and it's you've got to do it this way. And you've got all these different safety groups within that that, that pharma group, kind of not really joined up, not really talking to each other and it can be a sort of form of organised chaos.

So, I think Pharma is the sort of group that that is, you know doing registration trials are the worst thing really because of the demands of pharma are actually way in excess of the demands of the regulators. And there's this kind of funny disconnect because I get the impression that regulators are trying to be a bit more sensible about risk-based approaches. But pharma, especially when it comes to registration will just go, they just they just want they're immovable and. And so I think those, those, those are the challenges, the lack of internal consistency within some of the big regulators, different departments reviewing, you know, different trials within a platform and coming up with a completely different set of suggestions. And then and then pharma is a whole sort of world of pain or can be.

**Thompson, Jemima** 30:26
And do you feel that that's kind of similar like regardless of the phase of the trial or the disease area or the design 'cause? I know you've mentioned you know that with the adaptive trials for example, it seems to be a different kind of set of issues.

**Participant SR002** 30:42
So, I can't really comment about phase one. I mean I've done phase one trials in in when I worked in [Australia], but I don't have an experience of phase one in in the UK or you know working with the EU sites. So, I can't really comment, but I do think phase one trials have been done the same way forever and I do think we need to rethink on how they're done. But that's a whole you know that's it is not a- my area of expertise, but it just feels like we've done things exactly the same way forever. So, in my mind, you know, surely we should be rethinking that approach because we're rethinking the way that we're doing trials in general, you know, trying to make them more efficient, answering as many questions as you can in one go. So why is phase one, you know, you do this and then you do this, and then you do that, you know, it's nothing's changed as far as I can tell for decades. But I think, you know, even, you know, phase three trials where, you know, you know, you know, a fair amount of it's a definitive trial, for registration for example, I do think that some of the requirements are really excessive. And the other thing I never understand, and pharma never tells you, and I've asked them, they never tell you how they translate all of that information that you report, you know, grade one and two nonsense. How does that translate into the actual label? You know, the SmPC for that particular drug in that particular setting. I just have never known exactly what that process is, and I challenged [pharmaceutical company] when we had that [pharmaceutical company] meeting a few months back about how do you get from all of this data to deciding what actually goes into, you know, the reference safety information in section 4.8 of the SmPC. Nobody answered that. And when I asked my colleagues at [pharmaceutical company] or [pharmaceutical company], they're very vague about how it all, so I just never- I worry when there's a lack of transparency about how all of that data gets morphed into this table, this crucial table in section 4.8.

And you know, I would love to know more about that process and I suspect if we knew more about that process, it would reinforce the fact that collecting all this grade one and two data is just a complete waste of time even for registrational purposes, because I don't think it ever makes it into the reference safety information. But I don't know that for sure, but I would just love to know what the process is.

**Thompson, Jemima** 33:26
That's really interesting. Thank you, [SR002]. So if anyone else has anything to add that's really interesting, thank you.

**Participant SR001** 33:35
No, nothing. I'd also like to know, though, that was a good question.

**Thompson, Jemima** 33:38
Yeah, and I suppose just to kind of finish off on this section, I suppose kind of two things. Just that, you know, we've talked a bit about the reporting and I suppose things like all the documentation and things that you need to do for regulators.
How useful do you think that is? So, I know that you've mentioned [SR001] about kind of, you know, this duplication of work for MHRA and EU, and I'm guessing if you're working in other parts of the world, there's probably other hoops to jump through and things to do. And so I just wonder what your views were on that side of things and the actual documentation side of things like DSURs, all of that kind of that kind of stuff.

**Participant SR001** 34:27
Well, the DSURs, they've recently, the MHRA anyway, they've recently introduced. You have to pay for DSURs and I don't know about anyone else, but I know that we had real problems with them.

Not so we'd send the information they need to create an invoice, and then we'd get inundated with emails be like can you provide us with this information? We need to- can you pay this by this date or can it's just we've given you the information and it just wasn't working and now they have actually changed it so that you have to pay for the DSUR before you submit it, and I think that is so far we haven't got any kind of problems with that, but it's, I don't know, it's just it should have worked from the start instead of if you're going to implement something then it should work from the start.
And also submitting SUSARs and DSURs to the REC as well. They've also recently changed the process, so you have the CTIMP safety reporting form that you have to send and they used to sign it or just type their name into it.
Which they've now just said they're going to send back an e-mail, but you still have to send the form, but they haven't updated the form, so it's I guess it's kind of a cover letter, but it's more, they need to update that form because we don't need to sign it anymore. So, what's the point of sending it? I don't know, it just seems a bit like, again another thing that's changed that hasn't been massively thought through or they've kind of rushed it out.
Oh, and there's errors with ICSR submissions as well. They need to update the system. So, if you save a draught, go out of it, go back into it patient height and weight disappears. It's just a few errors here and there. Just techie kind of errors that seem to not. Yeah, and there’s, oh and acknowledgements as well. We don't get acknowledgments from the system anymore. I don't know if anyone else has had that too, but they've recently they've updated something and we no longer receive acknowledgements from the MHRA web for SUSAR submissions, and I've emailed them many times and they've not replied to me at all. So, that too.

**Thompson, Jemima** 36:36
And so in terms of your workload [SR001], I mean like what's that doing to your workload as someone working in PV specifically as well?

**Participant SR001** 36:47
Well admin, is yeah- it does yeah add on, especially when you have to keep chasing the more SUSARS we submit, the more things we have to chase for acknowledgements and if- the thing is, if they just said if they just said what, anything that responded to us once saying, oh, we're having some problems, please do this, but then we're just getting nothing from them. So, what do we do? We just have to keep doing our part and keep chasing them. And it does add a lot of time to have to keep track of all of this that's happening and getting absolutely no response and yeah, making cover lessons and things as well, like I, I don't necessarily know what they do with them, but it does add time onto them. Yeah, because, the thing is, look it, there's the information is in the submission itself. So, I don't know what the cover letter is for. Yes.

**Thompson, Jemima** 37:34
Thanks, [SR001]. Did anyone else have any thoughts about the actual kind of documentation and things like that and its impact on your workloads?
OK. So, I suppose the last thing for this section then was I just wanted to ask broadly to what extent do you think that the work that you're doing with your safety reporting is helping to maintain patient safety? [SR002]’s eyebrows are saying more than. [SR003], did you have something to say?

**Participant SR003** 38:38
No, I mean. We, I mean we've got to keep an eye on the safety of the patients but we I mean we always assume that the clinicians know what they're doing the other end. So yeah, I mean we hope they're reporting everything to us that we can keep an eye on it.

**Thompson, Jemima** 38:58
So again, it's kind of this, this team effort going on from the sites and for from you at this end at the CTU end.

**Participant SR003** 39:00
Yeah. Yeah. I'll say we can do everything. Our end just, you know, as much as we can. But we have to have been told about it in the first place.

**Thompson, Jemima** 39:14
Thank you, [SR003]. [SR002].

**Participant SR002** 39:17
I mean, I think obviously it is, it is important. But we are only a small part piece of the puzzle. Often for the same IMPs and at the end of the day it's you know, how all of the cumulative safety information is gathered together, including, you know, post marketing, marketing reports and so forth for drugs that are already licenced, at least in adults. So, I think that we will. We do contribute to the overall safety of that product. I have never been in a situation with any of our IMPs where any of our SUSARs have then translated into like a black box warning on an urgent safety alert. So, I don't know how that process really works as I've never been part of that.
But there clearly is a process that that, that, that happens you, you know, the drug companies and so forth will issue black box warnings when they've got enough information about, for example. I don't know. I think there was a black box warning for [drug name] and cerebral haemorrhage, you know, some years ago and that would have been gathered from different sources. But I don't know if anyone else in the group has ever been part of a SUSAR that's ended up being like a- an emergency, an urgent safety, you know, resulted in safety a halt or a black box warning or change the patient information. So, I think it would be interesting if anyone's been part of something that really you know resulted in a major, you know, major change, I think I think that would be, I don't know if anyone's been in that situation, I haven't. Fortunately.

**Participant SR001** 41:17
I don't think I've played a major part, but I think we did have a trial recently that with the, it was getting, a lot of the patients seem to get a lot of, there was a lot of incidences of pulmonary embolisms and I know that the TMG they had the meetings, they initially halted the trial and they changed the dose and they also added like a preventative, um, I think it was a [drug name] and they added that and then they- it happened again and I remember we had to report that and it was, kind of went from a temporary hook to a permanent halt.
But yeah, I wouldn't say I had massive role in it apart from just reporting the SUSAR, but I just it was interesting to hear that the people involved, like the trial teams and the sites that were involved in that, it was just interesting to hear about it. But yeah, my part was probably very minor.

**Thompson, Jemima** 42:19
Anyone have anything else to add on that point?
Gonna move us on, then. So, I know that we have these sections, but we've been having some really interesting discussions. So, this is all good. So, we've kind of touched on this a bit already, but I just want to kind of dig into this a little bit more about, safety reporting as part of your job and as part of your role. So, I know some of you. I know [SR001], so you're a PV person. So, it's kind of your whole job. But I just wanted to know kind of what role does it play in your typical working week? How much time are you spending on safety?
You know what, what are the challenges of safety reporting and juggling that with the rest of your workload?

**Participant SR003** 43:09
As Trial Managers, we sort of split it out between us and so, you know, if an SAE comes in, we get the alert and who if it's on the inbox that day will then action it.
So as such show we really are each on the inbox on the one day a week or but then we have to know if it's reported on to a clinical reviewer who will review it, then it'll have to go on. If it's a potential SUSAR, we'll have to, then we'll have to report it onto a Pharmacovigilance because again, it's a blinded trial. So that's been and beyond that, we don't know much more because if they say they've reported it, we know they were on active treatment, which we won't know.

**Participant SR001** 44:04
I mean for me, obviously it's a lot of pretty much all of my role, but I'd say SUSAR reporting in DSUR reporting is kind of my main priority, I'd say, half of my day is probably taken up by like this morning. I was looking at DSURS. Am I on track with those? I've got a SUSAR to report this afternoon. So, it's a lot. And then also, we're going to start looking at sponsor review forms as well. So, we're going to look at those just to make sure expectedness is done correctly and that we're not missing any potential SUSARs that we need to report. So it's yeah, it's a massive part. We yeah, we we're going to review all of the SAEs and follow-ups to make sure that we're catching everything we need to.
Yeah, it's pretty much all of my role.

**Thompson, Jemima** 45:07
[SR002], for you.

**Participant SR002** 45:09
Yeah, not too arduous for me because I split the role for being the person on [trial name] with my colleague [name], who does the lion's share of the work. But it's probably at least, I mean, it's 30 minutes to an hour a day for me and it's more for [name].
For the [project] work I'm back up Medical officer for one trial and I'm the lead medical officer for two trials.
And we haven't been enrolling very well on those COVID trials because we're in a very different phase of the pandemic. So in that it actually has been very, you know, very little work.
You know, well, we've only had one SAE in the last, you know, four weeks, which is a really a reflection of very slow enrolment but and that was, you know that was, you know, an hour's work, it was nothing so.
So I think I'm in an unusual situation where there's not much going on with one trial because we're not enrolling well in another trial where we're fully enrolled actually that the IMP has been incredibly well tolerated. So, we actually haven't had that much, but it's still, you know, half an hour, an hour a day for me a bit a bit more for Alistair, but still nothing, nothing really in the big scheme. Thanks.

**Thompson, Jemima** 46:33
Hmm. There, there's quite a lot of variation between roles. It seems as to how much time you spend, and I suppose just- Do you think that there is a way of measuring the time spent on all of these different tasks in a kind of meaningful way, that kind of reflects how this is in you know the, the proportion of your time spent doing safety reporting stuff. And do you think it's important to measure it?

**Participant SR003** 47:11
I don't think you can measure it meaningfully because it's again the type of trial or how safe the drug is and how many- I mean, we've only had 16 SAEs in 18 months and only one of those became a SUSAR, so, it again depends on how many you get in a trial. Other trials I've not had any.

**Participant SR001** 47:36
Yeah, it massively varies from trial to trial. How many SAEs and SUSARs you get. And even reporting SUSARs and DSURs they take such different amounts of time, like a trial that's in follow up, they don't really get any SAEs, so the DSUR will be very short, but a new trial or a DSUR that contains a lot of trials takes a lot of time. So, it's and also with SUSARs, yes, some can take about 20 minutes to report like a follow up that's just the change of outcome that's very easy. But a new SUSAR has massive amount of information, huge amount of tests. There's lots and lots of queries. It can take a real a very long time, so it's. I don't know if you can actually quantify it that easily and it to be accurate anyway.

**Thompson, Jemima** 48:21
Yeah.

**Participant SR002** 48:26
I think it's. I agree. I yeah, I think it's really practically impossible to quantify, but maybe one of the things that might be useful is some sort of assessment of what's the difference between someone who is less experienced as a clinical reviewer compared to somebody who's, you know, very senior and done it for a long time, not that they've, they may not remember how to do it all, but I think I think that that would be an important metric because, maybe that would lead to closer mentorship of more junior clinicians in this whole process which you, you know, perhaps the old timers amongst us, you know, can always do it on standing on our head because we've been doing it for long time. So, there's lots of things that we take for granted. But I think that when you're less experienced, less confident it, it may take you an awful long time and maybe there's room for improvement in terms of mentorship.

And that could also be the case for, you know, the trial teams, you know, the trial manager maybe very experienced or the very experienced CPM or maybe less so and so it takes them a lot longer and perhaps says there is room again for that sort of mentorship. Even cross trial mentorship, I can't. I can't think of. I think that might be an important metric to understand. You know how long it does take people who aren’t so experienced and can we do anything to change that and help them? Yeah.

**Thompson, Jemima** 49:51
Hmm. These are really interesting point that you’ve all raised and it it's kind of got me thinking about how this aspect of your jobs and of your day affects your performance and your well-being at work and things like that.
Do you think that there is a relationship there? Perhaps or not.

**Participant SR003** 50:21
You just accept it as part of your role, you know, and get on with it.

**Participant SR001** 50:27
In a way, the variation is quite nice because you know it's quite nice that, yeah, sort of, things are different. I mean, obviously there are some more stressful things than others, like a massive DSUR is quite stressful, but it's not- we don't have, yeah, I'd say overall it's quite nice to have the variation.

**Thompson, Jemima** 50:34
Hmm. Yes.

**Participant SR002** 50:49
I mean, for me, it's the only time I really feel that my clinical skills are being used.
That's really sad, isn't it? I mean, obviously when I do, when I when I do, when I do, when I do clinic, then of course I'm seeing patients and then I you know, I know I'm having to sort of think and and so forth, but this is about the only time because most of most of the work of trials is you know there's a serious administrative burden for everyone, right, you know trials are, you know, 99.9% admin aren't they and about .1% kind of science and other things. So, I think that's the truth, right. So, for me not that you feel that you.

**Thompson, Jemima** 50:57
Nice bit of practise. Mm hmm.

**Participant SR002** 51:28
You know can play a role in terms of your clinical experience and what you can bring to the table and you know how you can help your sites and you know engage with patient care without overstepping your remit. But also, you know the for me it's actually I don't mind safety reporting at all as long as the patients are OK. But I like using my clinical skills because that's what I trained for. This is my experience.

**Thompson, Jemima** 52:02
Makes sense. Makes sense. So I suppose I'm gonna kind of move on to kind of Section 4 the final kind of. The crux of the issue is improving safety reporting processes. Are there things we can be doing to improve the the safety reporting processes as they currently exist and what might be some of the facilitators and barriers to any changes we might make?

**Participant SR001** 52:42
Something that I can think of that we've had, some kind of what I'd be interested is going back to SmPCs. Some of these SmPCs are very for drugs that have been on the market for decades, so they just don't update them anymore. And the problem with that is with when you're looking at expectedness, if it you have to consider something unexpected if it's more specific than what's listed on the reference safety information. So, for example, if they've just put infections and you've got.
Lung infection, you can't we can't necessarily consider that expected because it's more specific than what they've listed. So for me, it would be if pharma companies can update their SPCs to be more kind of tailored to what we use the RSAI, what we use them for, which is expectedness. 'Cause then it would help with things that we, you would logically be like yes this - to anyone looking at this, you'd be like, yes, this is expected, but because the regulations say you have to anything that's more specific, you have to do, it's unexpected. You can't say it is, so it's quite frustrating when you have you kind of you're like, oh, I know this is kind of expected, but we have to make it unexpected and then it becomes a SUSAR and then it's triggered all the reporting and it's that's kind of the immediate I've that's something that, yeah, I've noticed that if. They could update the old SmPCs. That would be really nice.

**Thompson, Jemima** 54:20
[SR002].

**Participant SR002** 54:22
Yeah. So, I think one of the other challenges and is that we probably don't share some of the approaches that have been taken that have been successful across different trials. One example is something that we certainly used to do in my old unit in [place name] was we used to write investigator brochures.
Especially with working with generic companies where they really just don't have one and we sort of ended up having to do that and that's one of the approaches that we've taken in [trial names] where we're using, you know, the fixed dose combination of [IMP names] is is the is the control, the control drug in both trials.
And there are lots of different generic forms of [IMP name]. So, what we've done is you're basically look at look at all of those. Some of them haven't been updated since 2017 and others more recently updated like the FDA version because they've approved that generic formulation now.
And I've undertaken to basically write the reference safety information, if you like for [IMP name] and then separate components so that using you, you know SmPC’s and stuff from generic, you know pack packet inserts and so forth. And I think that is an approach that is perfectly acceptable and that is a mechanism where you could, you know up update the reference safety information and it would be completely reasonable to do that. You could draw from you know.
Post marketing reports and it’s perfectly OK to do that actually. And I don't think it's something 'cause when I first said this in the unit, everyone said, oh, you can't do that. So actually, you can, you know, it's completely as long as you, you know, have justified where you've taken it from and why you're taking that approach. And that could get round all sorts of, you know, endless SUSAR reporting just because a company hasn't updated the word infections. Or you take it upon yourself to you know you. You write the RSI and you say, by Infections, I mean XYZ, right? That's perfectly OK to do that, right? It's not an approach that's widely taken, but it's absolutely fine. So anyway.

**Participant SR001** 56:42
That's good to hear because I think we have actually that has crossed our minds. So, it's good to hear that you've not had an issue with that actually. So, thank you.

**Participant SR002** 56:52
I couldn't think of a way to do it unless we did it that way, right?

**Participant SR003** 56:52
Yeah. Yes, that suits us as well, because the wording didn't match exactly what was in the RSI, even though it came as part of it, it wasn't the exact wording.

**Participant SR001** 57:03
Yeah.

**Thompson, Jemima** 57:09
That's really interesting. So there's kind of a bit of - sounds like there's a bit of a lag or a bit of I don't put words in people's mouth, but almost like pedantry.
In terms of the regulations and the way they were having to follow things is quite specific that there might be room for a bit of bit of change in there perhaps. And do you think any of those, some of these kinds of challenges and limitations, do you think that - wonder kind of where they're coming from. Like, do you think it's- is it regulators? Is it the CTU themselves? Is it kind of a combination of the issues with sites kind of?

**Participant SR003** 57:55
It is the regulators because we were told, yeah, we were told. No, you have to have the exact words against the MHRA expect it. If they come to inspect.

**Thompson, Jemima** 57:56
I just. Mm. Which is interesting, if they're taking risk-based approaches.

**Participant SR002** 58:10
I think we could push back against that. I mean, if the word infections appears, I mean, an infection is an infection, right? So I just, I think that we're probably absolutely following to the letter of the law and we could push back against MHRA, you know, if the exact I mean, for example, and the other stupid thing about sorry, the RSI sometimes drive me absolutely nuts, is that you might have something in there which says, you know, raised liver Enzymes. But you don't have the word transaminitis. Well, it's one in the same thing, right? So, I think that, you know, sort of common sense has to kind of prevail. And I think you'd be perfectly in your rights to say liver, right, raised liver enzymes is the same thing as transaminitis.

And if people feel a bit like, oh, it's not the exact wording, then you can just, you know, put some sort of, you know, file note in there and justify why in your clinical opinion it is one and the same thing. So, you know there are workarounds if the exact words aren't there because you've got a word that means the same thing. Essentially, your own- you know, a phrase. But I think sometimes we we are we follow to the letter of the law, which I think it's good.

But I think if we're find coming across these things, we should, you know, try and sort of have a discussion with the regulator and say, you know, can we have a little bit of you know can we agree a common ground that might make this more sensible, because I'm certain the MHRA doesn't want loads and loads of users if they can avoid them. So I think we're living in fear of the inspection, but I think and so, it's like can we find a work around that works for us all include and would be, you know obviously acceptable to the regulator, it's stuff like that that I think you can push back much harder than you you have more power than we have more power than we think and I think we'd save just a world of pain you know. Just my opinion.

**Thompson, Jemima** 1:00:16
And I think so just to go back to a point you made earlier about the, kind of pharma being more risk averse. Do you think that that's part of it as well with the SMPCS in this this kind of relationship between that and the regulators and the wording?

**Participant SR002** 1:00:32
I think some of it's that it where pharma become a really big problem is with anything that's registrational. So, I see the difference for example in a registrational trial like [trial name] which is a registrational trial for the fixed dose combination of [IMP names] in [patient population]. And then my trial [name] which is, the biggest trial of [IMP name] in [patient group]. But it's not a registrational trial. So, we've had a lot more freedom in [trial name], for example, we don't collect grade one and two adverse events. We only report you know we just you've got to grade everything, but it's only, you know grade three and four clinical adverse events, not even the laboratory stuff unless you think it's clinically important. So, a lot depends on whether it's a registrational trial or not.
And the fiasco with that is that that we know even though we've been told in in [trial name] it's not registration trial. We know that they're going to come back to us to to put some of that data from our trial into the label for the [IMP]. We know that's going to happen. But the fact that it's not a registration trial means that there we have got much more freedom.

It's interesting. So that may be that [pharmaceutical company], you know a different company from [pharmaceutical company] and so, there are different rules even between companies when it comes to registrational work, some are less, you know anxious than others. It's not consistent, in other words.

**Thompson, Jemima** 1:02:21
And I suppose just to kind of round that off, I'm kind of going back to the point about, if we tried to think about improving how we do our safety reporting, what ways might so I mentioned you're kind of measuring you know, how effective might these changes be? Is there a meaningful way that you think that we can do that to say that you know if we put this thing in, we can measure it using this and look how much better it is like is there a way that we can kind of quantify in any way, changes that we make and whether they have been an improvement or not?

**Participant SR002** 1:03:08
I think we need to have a- another interim step and that is really sharing much more. some of our, you know, safety reporting processes across the unit. I think there's, I think within infections. We're probably quite joined up, especially as you know, we have people that work for example on [trial name] as well as [trial name] and [trial name] and we really tried to synchronise the way we do things right, which has been really helpful.

But I think other you know, I don't really know. That's just one part of infections I don't really know much about safety reporting within the [disease area] trials, even though that sits on infections, I don't do any [disease area] work. So, I don't really know that. So we're not very sort of joined up and then we could be and then in terms of what's going on in [other disease area] and what's going on in the with the [other disease area] trials, I don't think we're joined up at all and so, and you don't have clinical reviewers who sit across, you know you don't have somebody who is a clinical reviewer in infections who sits across and does [disease area] and [disease area] work. So you haven't even got that person who could sit across and give you a sort of well, we do this in this trial. Couldn't we apply that? So maybe I think there's another step before we get to sort of thinking about how we can improve things and that is a- we should have a coming together to share some of the things that we think have worked well, in in some of our trials and could work well if adopted in other trials.

I think that's I think that's important and one of the reasons, it's also even before even at the Protocol Review Committee stage because I was on the PRC for one of the [disease area] trials a couple of weeks ago and it was like it was like a revelation when I said why are you collecting you've got repurposed drugs you why are you collecting all this grade one and two you know AE stuff I mean.
Do you have to and the P- The CI was like, well, that's what I thought you had to do. It's like no, you know, push back. You're just going to kill yourselves with all of this. You know, people with [disease], especially advanced [disease] have got lots of ongoing problems. It can get worse. It's nothing to do with the IMPs, right. You know, you want to know what's really important. And so, this was like, Oh my God, if we if we can just collect grade three and four clinical adverse events. So, life is just become it much better overnight.

So, I just found that it was just, I just happen to be the clinical reviewer on that PRC. Otherwise, I don't think that would have been something that they sort of thought about as a possibility. So I think that we need a much better coming together of how we're doing things. For trials in planning trials that are obviously ongoing, that's more tricky, but trials and planning certainly before we get to you know what would be the metrics where we could measure you know how our processes are better as a consequence of change, does that make sense? Long winded, sorry.

**Thompson, Jemima** 1:06:22
Yes. Not a problem, not a problem. I don't know [SR001] or [SR003]. Did you have anything to add to [SR002]’s point?

**Participant SR003** 1:06:32
Yeah, I think it's a. It's been a case of, yeah, we've always done it this way. Just keep doing it sort of almost.

**Participant SR001** 1:06:40
I've noticed that as well. It's very much, people just do what they're kind of no know what it's said, and they don't think about well, why are we actually doing this? And I also agree I definitely think because in PV, I know that we do have like meetings every few months or so, sometimes more, sometimes less depending on what's happening with the other, some others clinical trial units just to see are we, what have you got any queries? It's just so we can kind of align with each other, and it is actually really useful and I think maybe we could do a lot on an even bigger scale in the sense of not just the PV people coming together, but hearing from the trial teams or the clinicians. Because as you say it's I think it's.

Do you learn well if they can do it, then why can't we or like kind of it's good to hear about other people's experiences with the MHRA in particular because they have to do have- you can ask one person something and someone else will come back with another thing and they're very vague with their advice like we've asked them advice and they'll say, well, you can do this and they'll have a caveat at the bottom of me like, well, but you have to follow XY and Z, which isn't clear. So, it's good. It's always good to align with other clinical trials units as well. Just so if you're all on the same page then.

**Participant SR001** 1:07:56
Yeah, power in numbers.

**Thompson, Jemima** 1:08:01
Yeah, it's a really nice point you’re making about yeah, working together with others and I suppose, just, it got me thinking as well about things like the SOPs.
And you know the committee's work together to put the SOPs together for safety reporting and how we do those processes. Do you think that they’re as efficient as they could be or are there things that we can do to improve those as well because they're kind of I suppose how we might join together to make sure everybody is doing the same thing to some extent.
But do you think that that kind of works or is enough?

**Participant SR002** 1:09:01
I've lost my train of thought. Say again?

**Thompson, Jemima** 1:09:05
So just thinking about the standard operating procedures and the SOPs that we follow, just you, you, you kind of mentioned about working with others and it just made me think about the SOP is the thing that joins together all the processes that we follow within the unit. And I just wondered whether you know how helpful that was, do you think it streamlines those processes enough or that there's more that can be done with the SOPs? Do we need to be following the stops as closely as we do?

**Participant SR002** 1:09:34
I think the SOPs are really important and they're well written, aren't they? And it is an absolute requirement for us to continue to be a CTU. But I think that, with all the changing landscape, I you know MHRA, apart from being in complete disarray for the last 18 months, as far as I can tell, it's sort of becoming good now, but it's hard for the SOPs to keep up with all of the changing- so I think that, perhaps, you know it is, the one SOP that I suppose you can't update the shop every six months and it has been a really it's been it feels like a revolving door of change at the moment.
So maybe it's about having a session where we summarise some of the changes because I think people are quite lost and th- and say that you know the planned date to update the SOP with some of these changes is going to be this. So, in the interim maybe you know, please ask if you've got any questions because.
'Cause you do, you do you see what I mean? 'cause I the so it's never gonna be completely on top of all the changes. So I think we probably have to be you know.

We are all aware of that. I can't keep up with all changes. You get some alert and it's like, oh, no, you don't have to do safety, annual safety reporting or something. It's like, well, OK. And, you know, I find it kind of hard to remember all of that and. And so maybe we do need a session on, you know, what's changed in, in, in safety reporting. What are the absolute, you know, what's the MHRA decreed now and then what's the timeline for us to update, you know, our SOPs just so that people.

You know could or implement what's already changed and you put in. You know we're deviating from the SOP because what we know the SOP’s gonna, you know, you could do something like that, right? But it's just about knowing what's changed. Does that does that make sense?

**Thompson, Jemima** 1:11:31
Mm hmm mm hmm. Yeah, that I thought that was very clear, very well explained. Thank you, [SR002]. [SR003]?

**Participant SR003** 1:11:43
No, I've got nothing else to add.

**Thompson, Jemima** 1:11:45
Good and I think [SR001]’s had to step away for a second.
You're back. So just [SR001] did you have anything to add about the SOPs and the way that we use them and?

**Participant SR001** 1:11:59
I think I agree with [SR002]. I mean, sorry if I missed the if you said this, but I think
training alongside the SOP is really important, so if you do have a lot of updates, I think it is quite good to just have a few training sessions because I think people will take it in more. If you be like this as you say like just have like a session of this is what's changed.
Yeah, 'cause, I think leaving people to just read the SOP. I don't if it necessarily goes in, so I think it's always just important to have some kind of training alongside that as well.

**Thompson, Jemima** 1:12:34
Thank you so very, very last thing. Is there anything else that I suppose about safety reporting and all the processes that you've been burning and wanting to say? And I haven't directly asked about? Your input is important, so if there's anything you want to say, now is your opportunity.

**Participant SR001** 1:13:01
I don't think so.

**Participant SR003** 1:13:04
No, I haven't.

**Thompson, Jemima** 1:13:08
And [SR002], that you were shaking your head when you as well so. OK. In that case then.

**Participant SR002** 1:13:16
Thanks.

**Thompson, Jemima** 1:13:16
It just remains for me to say thank you, all of you for your time today. It's been really good hearing all of your views, hearing what you have to say, and hopefully we can use some of this information to start trying to think about how we can make sure that we're doing safety reporting to the best of our ability and you know helping patients and you know having a nice work environment to be in as well. So, I'm gonna stop sharing so that I can see you all properly.


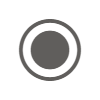
 **Thompson, Jemima** stopped transcription

**Transcript Focus Group 2 10-Jul-2024**

10 July 2024, 01:09pm


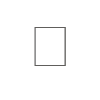
 **Thompson, Jemima** started transcription

**Thompson, Jemima** 0:10
Is just what do you all understand by safety reporting and pharmacovigilance in the context of clinical trials? Go on [SR004], you've unmuted.

**SR004** 0:34
I guess it's like an all-encompassing term to talk about collecting, analysing and recording reporting data on safety of things that are being tested in a clinical trial situation. Yeah. So that's quite a broad term, but yeah, maybe that covers a lot of it.

**Thompson, Jemima** 0:56
Thank you, [SR004]. Does anyone have anything they want to add to that or disagree with that? All happy we’re talking about the same thing.

**SR005** 1:07
And just to add to that. So, for our safety, we're reporting would be sites recording adverse events and reporting events which are serious to us at the [CTU] and from there, we would review whether they're SUSARs and if they are, we would onward report them to the MHRA, REC and any other competent authorities.

**Thompson, Jemima** 1:33
Thanks, [SR005].

**SR004** 1:34
I guess there's sort of different levels where the safety reporting is happening. There's site level, the clinical trials unit and then even broader than that, like the MHRA. Reviewing all the safety data that's coming into them.

**SR006** 1:49
I'm just going to add as well that it's not just related to drugs that are new, but also drugs that are already on the market as well and how they might affect different patient populations or any other like unknown safety effects that have been discovered.

**Thompson, Jemima** 2:10
Is anyone- anything to add to that at this point?
So we're all kind of working from the same page. This is good. So just- I know we kind of couple of you kind of touched on this, but what would you say are kind of some of the main tasks and processes that make up safety reporting and pharmacovigilance?

**SR006** 2:36
I think one of them is just trying to define what your what safety reporting is relevant for the study that you work on. So you're not always reporting every single event that happens.

**SR007** 2:55
Sorry, I've, go on-.
 **SR009** 2:59
Yeah, I agree with [SR006] because on the trial that I work, we only collect safety that’s related to IMP. Those that are not related to IMP, we just ask the sites to just enter those on a log, whereas if it's related to IMP, we ask them to expedite that to us within 24 hours. **SR008** 3:26
Yep. So, it's similar to what I was gonna say, really. So on [trial name], we do tend to refer quite a lot whether it's related or not. So we do- so yeah, we might have for example on the metformin and [trial name] or [trial name] even the patients progress, you'd think you'd stop reporting, but we'd still be reporting until the patient stopped the IMP as well. So, it's defining what's an SAE, a SAR, a SUSAR or even not an SAE a lot of the time as well.

**SR007** 4:01
Yeah, we report in the same way as [four trial names] as well.
So whether or not it's related or not to the drug, we know about it and classify it.

**Thompson, Jemima** 4:17
Thank you. Anyone else got anything to add? I don't.

**SR010** 4:20
I'll say. Oh, yeah, sorry. I'll say also, just having a clearly defined procedure documented. And just in relation to what everyone said, making sure that all aspects are clear. And everyone knows when things should be sent and what should be sent, when it should be sent and also the sorts of information as well that would need to be collected.

**Thompson, Jemima** 4:48
Thank you. And [SR006}.

**SR006** 4:51
And it's also having, like periodic review of the, I mean I'm working on IMP trial, so maybe relevant for like devices and other things as well. The periodic review of the
intervention that you're using to see if the safety aspects have changed and that might affect your review of the SAE, whether it makes it expected or unexpected.

**Thompson, Jemima** 5:14
Thanks, [SR006] and [SR004].

**SR004** 5:17
Yes, I'll so again to add to [SR006]’s point. So collecting all the adverse event data and then that will be reviewed in IDMC at some point and also, not just review of the RSI, but review of external trial results, which may be relevant to what's going on in your trial to justify ongoing risk benefit. Sort of justifying continuing with the trial as well, like in the DSUR report.

**Thompson, Jemima** 5:46
Hmm. Hmm. Thanks, [SR004]. And just to go back to [SR006]'s point about CTIMPs and kind of non-CTIMPS, do you think that there are big distinctions between CTIMPs and non-CTIMP and ATIMP trials? I don't know if anyone's got experience across those different trial types.

**SR009** 6:10
So I've only worked on a CTIMP trial so before aseptic I used to work on [trial name], but I felt like on [trial name] one everything was collected so adverse events and serious adverse events regardless if they were related to or not related to IMP I think when I moved trials into [disease] I just found the concept a bit different and weird because I was under the impression you have to collect all serious adverse events regardless of if it was CTIMP or a non-CTIMP or even an ATIMP.
And – when - it took me a while to get around that concept where we were only collecting SAEs related to IMP, but I guess it just based it's based on the trial specification and the requirements.

**Thompson, Jemima** 6:59
Hmm. Thanks, [SR009]. [SR005] you had a hand up.
 **SR005** 7:19
So, we've got a number of -18 trials running here and the biggest difference with them compared to our other CTIMPs is that in their reference safety information, we have no events which are considered expected. So, any events which are serious and related would all automatically be assessed as a SUSAR and we would report them to the MHRA and the REC. And the difference with the non-CTIMPs and the CTIMPs is with our non-CTIMP we would only report only report, related unexpected SAEs to the HRA, whereas we would report all related unexpected serious adverse events to the regulators and the REC.

**Thompson, Jemima** 8:06
Thanks. And just building on the points, a couple of you have made, I just wonder like all of these different tasks and things that we have to do and these different processes, do you think that they're all necessary, or are there any kind of ancillary things that you kind of think? I don't really know what you're doing. [SR005]?

**SR005** 8:32
Oh, have I got my hand raised?

**Thompson, Jemima** 8:33
You still got.

**SR005** 8:36
OK. In terms of what's- I think in in line with the regulations, I don't think we're doing anything that doesn't need to be carried out.
I think one of the hardest things for us is that with when we report SUSARs when we've got trials in the EU, we have to report the SUSARs into Eudravigilance and then report again to the MHRA into via ICSR submission, so that is a time consuming job. I mean, [SR011] you can tell you more about that. She she's the one that does it, but it's dual reporting. It's exactly the same information that goes into both systems and we were hoping we could report into one and then import the XML into the other system. But that doesn't work.
So I think that is that is a bit of a frustration on our part and we do have that a couple of international trials where we have to do that.

But I don't know if [SR011]'s got anything to add there.

**SR011** 9:46
No, I agree. Especially when you the Eudravigilance submissions portal is completely different in terms of what you enter on the portal compared to ICSR. It's like yeah, it is double work.

**Thompson, Jemima** 10:03
No ideal. Thanks, [SR011]. [SR008]?

**SR008** 10:11
Yep, add on to that. So, I think it's fine when you're reporting to just the MHRA ICSR portal. That's if you do it a few times, it's quite straightforward. But the addition if only there was a way to link MHRA, a portal to REC, it just always feels like it's extra admin and extra paperwork when you've already reported one thing to one, yeah, it just feels like additional an additional step that's not really required, although I think has that changed now that SUSARs - REC don't need to reply I think.
you can just send them the report so.
Could be a change maybe?

**SR005** 10:51
It has changed to. Oh, sorry, I should have put my raised my hand.

**SR004** 10:57
Do you want to respond to something first and then I'll raise a separate point.

**SR005** 10:58
I was just going to to say it has changed for trials that are now opening through combined review. You would only submit SUSARs once.
And that would go to the MHRA, and if necessary, they would onward report that to the REC. So, there is a step cut out there, but again, it's only for trials that are open through combined review.

**Thompson, Jemima** 11:24
Is this the new CTIS process? A combined review.

**SR005** 11:31
No, that's something that's different.

**Thompson, Jemima** 11:34
OK. Thanks, I wasn't sure. [SR004], and then [SR006], I think.

**SR004** 11:44
So I was just going to say it and I I think they're sort of aligns with the direction that things are travelling, but sometimes it feels like there could be more risk-based approach. For example, when we're using repurposed medicines where there is a lot of established data about them and I take the point that we are testing them in a slightly different setting that they would be prescribed in normally, but they will have been prescribed in the same populations just by chance. For example, metformin in men with prostate cancer, there'll be loads of people who've been prescribed.
That treatment whilst they have Prostate Cancer.
And it just it would be - I wonder how much useful information is generated by that process of submitting SUSARs about patients on combinations like that, where there's probably been hundreds of thousands of patients already treated, and whether you could think of a process for things like that where it could be maybe slightly less onerous for data collection and reporting purposes.

**Thompson, Jemima** 12:47
Thank you, [SR004].
Yes, very interesting point. I'm gonna come on to your risk-based approaches shortly. You've pre-empted me, [SR006].

**SR006** 13:01
Mine's just a small one. But I can't remember what it is now. It's something like if the site investigator thinks that the event is related to IMP regardless of what the clinical reviewer thinks it has to be reported as a SUSAR. If it is unexpected.
Obviously it's not for our - we don't have that many events happening, so it's not too can time consuming for our clinical reviewers, but they were very confused with the first one came through and kind of thought like what's the point of that process if- if one person thinks it, then it has to go forward.

And I can't - from what I can tell when you upload it on when you do report it, I think you can put what the site puts and the CI puts, but I don't know if that's actually considered like, could that just be a step that's removed If the site thinks it's related and they've given their rationale for it, and then you just report it without the clinical review. I guess you're gonna do the expected this assessment, don't you? But-

**Thompson, Jemima** 14:00
It's just that's a question for the clinical people in the in the group. So, I've got [SR004] next and then [SR007] and then [SR005].

**SR004** 14:12
Yeah. So I was just sort of, I mean, one inefficiency that frustrates me sometimes.
It's not really an extra process, but is that all events where the site haven't completed causality go for urgent review because we have to do a conservative assessment and say it might potentially be related. But it's just frustrating because like, I mean, in some periods we do 10 SAEs a week. So, many of those it's just because the research nurse hasn't felt confident to report those. So, we end up reporting lots of unnecessary SUSARs. Whereas if maybe you change your safety management plan to allow like a three-day period of trying to chase that missing data, you might avoid things having to be reviewed twice and unnecessary submissions to MHRA. And I appreciate there are tight time frames around all of this, but it just seems like just for lack of having a bit of flexibility to chase missing data, you end up doing lots more work.

**Thompson, Jemima** 15:15
And do you think, [SR004], just to kind of expand on that, do you think that expanding or giving that bit of flexibility would have like a major impact on patient safety?

**SR004** 15:29
So I guess in the trials I work in, probably not and I guess the with I would still be hoping that would keep within like the seven days for seven days SUSAR 15 day for 15 days SUSAR. So, it wouldn't actually mean that we are changing our sort of reporting responsibilities. It would just be how we actually deal with it in the unit. So, I think we'd still be meeting all the legal requirements. And also, I think like if you saw something as a clinician that you thought this is really important is really important. You wouldn't like- you don't need that information. You could - you could make that decision, but most of the stuff that comes in is, I guess, then you're saying that you need to see it that quickly to make that have that knowledge. But yeah, I don't. I don't know.

**SR006**16:13
[SR004] can I just - can I ask a question; if you're over reporting because this is what our CI’s were worried about with, you know, if they disagreed with the relatedness is if it's over reporting that would have an impact on the safety profile of the IMP or like, would you be for your trial, would you be withdrawing the ones that are found to be-

**SR004** 16:15
Yeah.

**SR006** 16:37
Like unrelated, that's what happens?

**SR004** 16:37
Yeah. Yeah. So, when they're finally like once - if it is ever confirmed that it's not related, then we always withdraw it. But also like in my- it's hard because there's not in my opinion that sometimes we get people who don't really understand the question “what does causality mean?”. So, I have things that I've we have reported as SUSAR to the MHRA, which I promise you were not related to the to the drug that they put it as related to so.

**SR006** 16:56
Hmm. Yeah.

**SR004** 17:08
That feels a little bit concerning that we're going to end up skewing the actual safety data that the MHRA receives, but then at the same time, like who am I as one person who has one site of this data, maybe they got a much broader view so they can come to different decisions. But when we've submitted about, I don't know, 50 SUSARs that are saying [drug name] causes [symptom/condition] because we gave it in combination of [drug name], then it just feels a little bit worrying that if that ends up in the safety information, it's not really useful data for people to have.

**Thompson, Jemima** 17:47
Thank you. [SR007]?

**SR007** 17:53
I think the fundamental problem with how long safety reporting takes is, as [SR004]'s alluded to, incomplete or incorrect information on a safety form that happens at site and ultimately you can never- you can question things, but you don't have that patient in front of you and you never really know what's fully said and you can't really go against it and you shouldn't obviously, because that impacts, you know, its bias and it that impacts what you send to the MHRA.
But a lot of the time, if you ask for more complete information and questioned it with site, then they would often come to a conclusion that something is unrelated.
So I wonder if more training at sites about how to report SAEs is probably the one way to improve how they're reported and how we are able to classify them.
And I compare that with [trial name], because we'd have an India arm of that trial, and their ethics is very different.
When they submit a safety report, they don't just submit our SAE form. Their ethics requires them to do an entire analysed report, which is over and above anything you would probably need for half of the events, but at least you can kind of say they've looked at all the information and this is how they've determined their causality. And the other thing that India ethics requires you to do is for you to give your assessment of causality based on all of that information, to see if it matches theirs or not.

I don't really think that's necessary from our end. We've obviously got a very different ethics and most of the time you agree that there is sort of a difference between countries and how things are reported and I think completeness of information is the one reason we always send queries back to site. That's all I’ve got to say, yeah.

**Thompson, Jemima** 19:42
Thank you, [SR007]. Thank you. I think I'm going to move on to [SR005] and then if no one's got anything to add to that, we'll move on to the next section, if that's OK, [SR005]?

**SR005** 19:56
Yeah. So just to add to the conversation earlier about.
The clinical reviewers causality assessment, which I can you hear me, there's an ambulance screaming past -

**Thompson, Jemima** 20:08
Yes, Oh no, we can hear you.

**SR005** 20:09
We, have just dropped the clinical reviewers assessment because it's only the site investigators assessment causality assessment that the MHRA are interested in.
So that's the only one we need. And I know I had a discussion with other PV managers and some places are still doing it and many are not. So, I know the [University] sponsor office are not doing a sponsors clinical causality assessment.
So we have just dropped that, which and we're just introducing that now. So that should that- should that should cut down our reporting quite a lot because so our clinical reviewers can't downgrade a site reviewers’ assessment. So, they can't say it's unrelated if the site reviewer has said it's if the site investigators said it's related then we have to consider it a SUSAR. But what they can do is they can upgrade it and quite- and so often we've had the site investigator saying an event is unrelated and then our clinical reviewer has upgraded it.
And yet the MHRA aren't interested in the clinical reviewers assessment. So, and that's why we have we have dropped that. Yeah. So that should simplify things for us.

**Thompson, Jemima** 21:27
Thanks, [SR005]. [SR004]?

**SR004** 21:29
Oh, sorry, I just wanted to ask a really quick question. What do you do if they don't complete causality? Do you just assume it's possibly related?

**SR005** 21:37
If the site doesn't complete it yeah, we if the site doesn't give us a causality, then we assume it's a related until they tell us otherwise.

So, we will still be sending our SAES for clinical review. So, the clinical reviewers will review each and every SAE as they come in, but they just won't be asked to do a sponsors causality assessment.

**Thompson, Jemima** 22:05
Thanks everyone. Did anyone have anything they wanted to add to that before we move on? OK. So, we have kind of already touched on this.
And I know [SR004] explicitly said talked about risk-based approaches, but it's something that I wanted to know a little bit more about. So, I just wanted to get.
An overview from as many of you as possible about your thoughts on the regulatory requirements in the UK and internationally. If you work internationally, you know how efficient are they and all of those things kind of what are our risk-based approaches being used in your trials? Are they being used most effectively? I throw this, open to all of you. Deep in thought about the regulations.

**SR004** 23:26
Just I guess I was just gonna say, I guess lots of the decisions about how things are done in a risk-based approach are probably put in right at the beginning of planning a trial and I don't have lots of involvement with that to know.
What bits have sort of been affected and risk-based assessed so?
Maybe some people here would like if you're working in established trials, you don't. You don't have so much insight into that. I feel I don't.

**SR007** 24:03
Whenever you do a new sort of trial on the unit, don't - there is like a risk form that everyone fills out, isn't it? Is that what you're sort of referring to and then alluding safety reporting based on the risk scoring system of that? Or do you mean based on the RSI of the drug and what you already know about it?

**Thompson, Jemima** 24:17
So I guess kind of both or all or neither of those things so.

**SR007** 24:25
OK, fine.

**Thompson, Jemima** 24:28
I suppose I just wanted to keep it quite broad to see what people understand by risk based approaches and because, like the MHRA and some other regulators are trying to move towards these more risk-based approaches and I just wondered if anyone had had experience of that or like what that means to all of you.
I mean the fact that I'm hearing silence suggests that maybe either it's the way I'm asking the question, or that there's not a clear understanding of maybe what risk based approaches are to safety reporting

**SR006** 25:04
Would that be like to an art trial, for example, because it's already a marketed drug?
Where only we only want that was reviewed at the beginning of the study during my setup and it was decided that only like grade three and four events are going to be reported and then SAEs as well.
Sort of like I can't remember who mentioned it on here, but someone on here mentioned that they're only reporting like related events, rather than all of them, I guess that that's a risk, but based approach isn't it?

**Thompson, Jemima** 25:35
Yes, exactly. Exactly. Yeah, that kind of thing. So, you- only you're reporting your higher grades. If you already kind of know the safety profile of the IMP and stuff like that.

**SR006** 25:48
Yeah, we had as well one of our DMC groups meetings did actually request that some of the narratives were more information was provided in the narratives as well. So, like I guess that changes as well depending on what their review is.

**SR007** 26:15
I think some of it probably comes down to trial design as well. So, I'm just thinking of like blinded [trial name] trials where you have- the highest dose of [drug] or whatever in a new population, and maybe that adds to the risk of how you report for it, even though obviously a lot of that won't be relevant later down the line. So I think it's - I think it's probably referring to sort of the drug itself, the dose of the drug in that population and sort of how it's used in the trial.
Which would probably require like a sort of independent stratifying system at the beginning of the trial for you to decide what events would be important in the worst case scenario if you like. And, yeah.

**SR004** 27:06
I was just going to say two comments. So, the sort of the small bits of risk-based approach that I've been involved with was decisions about what was going to be exempt from expedited reporting and what was not going to be exempt. And the idea that a less risky trial, you can exempt more things from being reported, but then actually the process is to manage that became even more complicated than just reporting everything, although some of the other risk-based approaches about maybe only reporting certain things sound like that would be time saving and efficient. The second point I was going to make was I guess the thing that makes me nervous is that when you we've made these decisions locally that were taking this risk-based approach and we might say we think it's low risk and for that reason we're going to take this approach. But it's- the only feedback I'm aware that we get that about whether that's meeting the MHRA requirements for managing the risk appropriately is when we submit the protocol and I just - it's never clear to me that they've how fully they've reviewed that, because there are things we've put in that protocol that we're doing that when they come to inspect us and they say, well, that's not right. But then if they're not reading the protocol and telling us that at that stage, I think it just makes you worried and probably always act in the most conservative manner possible because you just want to be making sure that you're trying to meet the requirements. So better, like interactions and feedback or more help from the MHRA of what you're doing is appropriate might help us. Be more risk-based approach and more appropriate way.

**Thompson, Jemima** 28:43
Mm hmm. And can you is it possible to reach out to the MHRA to ask them for, like, more support? Or is that not really the done thing?

**SR004** 28:54
Like in one trial, we used to have someone that we could reach out to ask for help and but that person's left and now we were asking for help with another problem at the MHRA and it's just very much like, no, you just have to go down like, read the frequently asked questions and just do everything that tells you on there. And there's no one you can actually speak to is is my experience recently anyway.


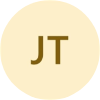
 **Thompson, Jemima** 29:15
Hmm. I suppose it kind of that leads me to ask then, do you think that the way some trials are doing safety reporting might be more conservative than they need to be, or perhaps not conservative enough, in your opinions?

**SR004** 29:45
Without understanding the flexibilities of the system it's hard to say. I feel like I feel like the trials I work on take a safe approach. Definitely a safer and maybe some of the things we do are not required.

**Thompson, Jemima** 30:03
[SR007]?

**SR007** 30:06
I think just drawing upon that, I think we're interested to know what people thought about elective procedures in long term follow up trials and whether or not they need to be reported as SAEs all the time because trials which are using repurposed drugs and have a really long follow up a lot of patients going to get admitted for various reasons, even if the sponsor and the site feel it's completely unrelated to the study drug. That feels like a slightly wasteful aspect of safety reporting for sort of an elected expected procedure.

I don't know what other trials are doing, but that takes up a lot of time on some of my trials.

**Thompson, Jemima** 30:58
[SR008]?

**SR008** 31:00
Yeah. So on [trial name], we don't really report a lot of the elective hospitalizations. I mean, it seems like we are constantly reporting SAEs, but that's just because of the volume of patients we've got. But yeah, and that is in the protocol that [SR004] alluded to earlier that it is part of the trial exemption. So, if it's an elective procedure or surgery, we don't report that, but it doesn't really feel like we're not reporting as much just because we are constantly reporting all the time.
And yeah, so maybe that's something to be added to the protocol at the beginning before we even proceed to anything with safety.

**SR004** 31:42
So, but just in just in response to [SR008]. Sorry [SR012].
Just to say quickly, so in in [trial name] when we submitted the protocol, we said we weren't going to collect elective events as expedited SAEs. But then and we sent that protocol to the MHRA which was approved and then we subsequently heard actually you still need to submit the data, but you just don't need to do it in an expedited fashion and then it required quite a lot of reworking of the CRFs to collect the data we needed to submit them as SAEs on the DSUR at the end of the year. So, we sort of tried to do something risk-based approach because we thought, oh, we don't need that data, but then it ended up being a complication down the line because the MHRA hadn't told us that what we'd suggested wasn't quite right. So yeah, it was just. It's just a little bit difficult sometimes working out - that you're following the rules and not getting feedback on whether you're following the rules or not.


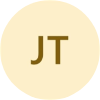
 **Thompson, Jemima** 32:40
Hmm. Thanks, [SR004]. [SR012]?

**SR012** 32:45
Yeah, it just made me think of something slightly sort of the opposite of that 'cause. I work on a lot of trials for [drugs] that already licenced in adults that we're using in phase three in children that have huge amounts of safety data and it's more of sort of treatment strategy, type trials. And then we'll occasionally we thankfully don't get a lot of events in with the world kids and it's when you get a complex medical condition diagnosed incidentally within the trial such as malignancy.
And then it becomes sort of - a quite complicated safety reporting for lots of the complications and treatments of that malignancy within the trial for that patient that obviously completely unrelated to the IMP and continue to be so throughout the trial.
And it although I mean there's nothing compared to the oncology trials, but it does result in a lot of superfluous reporting I think for regular sort of weekly admissions for chemo and things like that. That obviously isn't anything to do with the IMP, why does that involve huge amounts of data entry for the site team etcetera that's unnecessary.

**Thompson, Jemima** 33:50
Thank you, [SR012]. [SR006]?

**SR006** 33:55
And mine's more related to like the actual, so we use DAIDS grading scale for our events and other than just having this like PDF document that we look at and I think you can look at some like slides online, there's not actually, like a proper training for it, I sort of had to be quite self-taught in that sense. And then also like seek the guidance of the CI’s and like the other trial team members. And that's what the sites are using for their, for their, for their grading, their things as well. And they've a lot of sites have far less like resources to like safety training, that sort of thing, I guess it's kind of like outside the scope of this, but if there's if everyone wants it to be reported in a kind of standardised way, it feels kind of a little bit - it feels that people might be doing it differently, a little bit open to interpretation in the way that they're grading it.

**Thompson, Jemima** 34:46
Hmm. Thanks [SR006]. I don't think that is outside the scope of this. I think it it kind of. You're actually going to lead me into my next point a little bit.
So yeah, about sort of training needs, I don't know. I'll tell you what. I'm just going to move on the slides because I wanted to ask people more about kind of, you know, within your roles and training and things like that. So, I suppose to build on what [SR006]'s just said, does anyone else have any thoughts about training needs with safety reporting and guidance in in that respect within your, within your, within your actual working roles?

**SR006** 35:26
I think mine would be like training the people doing the safety reporting which what said so how to do that in a like SIV training for example.

**Thompson, Jemima** 35:35
Mm hmm mm hmm. And is that something that is generally done in - So I don't know how many trials you've worked on, [SR006], but is that something that you've done as part of your SIV? Is the pharmacovigilance training with the sites?

**SR006** 35:47
Yes, you always do safety training and when I worked in hospitals, there was always a safety training aspect and the training is normally there's like a generic aspect to it, like what is an SAE, what's a SUSAR, that sort of thing. And then the specific requirements for the trial. But you've got a very diverse experience of medical staff work- on the calls. And we've also got we're working like 12 different European countries and I think people it can be quite a boring training to deliver when it's all just the generic stuff.
And we found that, you know, we'll have to deliver retraining, like sometimes we've had investigators doing late reporting of SAEs or not really knowing that they needed to report SAEs if it was, even if it's related to like, progression of the illness, for example. So, it's trying to like some training, how to deliver a good training so that they actually understand the requirements of the trial would be helpful.

**Thompson, Jemima** 36:43
Thanks [SR006]. [SR007]?

**SR007** 36:47
Yeah, I completely agree. And I think I said earlier as well, I think training at sites is probably improved. I think we all assume by having GCP training and everyone submitting their certificate that they've clicked through sort of assumes that everyone's got a understanding of how to safety report. But it needs to be more trial specific and potentially just half an hour after an SIV, or in the week of an SIV with a worked example of what is actually required for this site would be helpful.

**SR004** 37:22
I think.
There are elements of the training that I've had that, so some things that I've had to do which just aren't helpful. So example, it was recently added that we had to have MedDRA training and it seemed to be the view of everyone else that if we were having this MedDRA training, it was going to be like teaching us how to code stuff and it's going to be really informative for like all the non-clinical people that are, like, very reverential about this and then. But the actual MedDRA training, it was like, so useless. It was just talking about how the database was formed and the extent they spoke that that's what we're expected to watch. And there's nothing that actually really talks about how to use it on a more practical aspect.
And so I think sometimes there's a slight misconception about the how useful some of the things were asked to do actually are. That's one of them. And then the other thing is, I think there's still some things that even though we have the SOPs in place and the safety management group, there's some things that are still a little bit ambiguous. So, there's things that I still have like, very, I know there's variability in practise and how I do things compared to other reviewers. For example, one of the things I'm not clear about is.
How precisely the site need to write the main event term to the term that I code it and some trial teams say that you have to correct them so they're exactly the same on both pieces and some say, well, if it's exactly the same, you wouldn't need a clinical code. It's obviously acceptable that you can code it to something more - so. So even though I've had training from senior people in the in the workplace, I get told different things by different people so it's just interesting that there's always going to be things that are a little bit ambiguous, I think.

**Thompson, Jemima** 39:12
Hmm. Thanks, [SR004] that’s a really good point. [SR006]?

**SR006** 39:20
This one's more of like a trial management admin kind of side of things as we've moved on to flex now. And like eTMF like where to file everything and that's like, there are a lot of a lot of saved documents. But and also, what do you need to file? So like I think when you up report a SUSAR, you need to save the receipt and do an export and save the XML. But we've sort of had to figure that out as we go along and probably be helpful if that's shared unit wide.

**Thompson, Jemima** 39:52
Do you think there's more the units could potentially be doing in terms of sharing information and kind of helping alleviating some of our ambiguity that [SR004] was talking about as well.

**SR006** 40:11
Potentially, I think with our unit, they're obviously working towards that.
Trying to share like trackers that people have done and that sort of thing. I don't think it's completely there yet like everything we've done for our trial has - ff it was not on SOP Box, we've just completely created it ourselves and not seen someone else's. It's not like I definitely don't think they're perfect spreadsheets in the slightest, but you know, certainly people within the unit have better skills on Excel than I do.
But in terms of like across CTU to CTU, I'm not aware of any like sharing information.
I think that the safety group has been mentioned a couple of times, but.

**SR004** 40:55
I wonder if, like some of the ambiguity like I mean the more people you speak to, the more opinions you might get on how to do it. So just like more getting more cooks, it needs to be maybe from the top. There needs to be more detailed descriptions of what is required for each for each aspect, from the MHRA. So we don't have so much ambiguity maybe.

**Thompson, Jemima** 41:22
Hmm. Anyone have anything to add to that? Those points. I suppose my next kind of question then would be how or sort of, what role does safety reporting play in your sort of day-to-day working role and you is there a typical week? Does it vary? What does that look like for all of you?
[SR009].

**SR009** 42:03
So for me 'cause, I'm the only trial manager working on my trial. It's really ad hoc, so whenever safety report comes through the inbox, but we actually don't get many very often. So, it's quite chilled. Yeah. So, it's as and when really.

**SR008** 42:28
So for me, we've got quite a- we've got a safety team just because of the numbers that we're getting. So, it's almost like a daily thing for me.
Pretty much every single day we're processing SAEs or sending them for clinical review. We've been getting a lot less SUSARs lately. Probably just because of sorry. We're getting closer to the end of the trial. But yeah, it is a daily occurrence for me.

**Thompson, Jemima** 42:56
Thanks [SR008]. And do you think that there is a difference in sort of where your trial is at in its sort of life cycle as to what safety reporting looks like?

**SR008** 43:08
Definitely. And I think it's the size of the trial as well. I mean, [trial name] is huge. So, it makes sense for us to have a number of trial managers as well as data managers as backup on there. Umm, so yeah, I think it depends on the number of patients. You've enrolled quite a lot of the time.

**Thompson, Jemima** 43:12
Mm hmm. Thank you. [SR007]?

**SR007** 43:28
And yeah, I think it also comes down to a risk thing and the drug and for instance in [trial name] because again, it's sort of nearing the end of recruitment in the UK and only open India as of next week, they've been sort of sending SAEs on like a two weekly basis, unless there's anything that seems like a SAR or SUSAR. And that makes it much more manageable clinically because you can just sort of bash them all out all in one go. And actually, I've just had my e-mail of my batch of SAEs this week, which is very timely, but it does allow for better time management from a clinical point of view and probably from a trial manager point of view as well. Because if I send loads of queries it tends to be all at once. When I've reviewed all of them which they can send out as well. So maybe depending on where the trial is, batches depending on, you know the risk of the trial is kind of a good way to approach it. I found it quite helpful. And I'm so sorry. I'm going to have to leave in 2 minutes.

**Thompson, Jemima** 44:33
Yes. No, I know you'd said before. Thank you, [SR007]. Yeah, I appreciate your input today.

**SR007** 44:35
Yeah, no worries.

**Thompson, Jemima** 44:39
[SR012]?

**SR012** 44:41
I guess I can only give the point of view of someone that works very part time for the CTU, so I'm employed half a day a week to be a clinical advisor on several trials and, yeah, I guess it being part time and the nature of safety reporting is quite difficult to balance out, especially if it's across several trials and with very few clinicians working on them. And so effectively I'm on call all year, every day, safety reporting, even though I only work half a day a week, unless I very much say I'm on annual leave for these two weeks, someone else has to cover then it can expand into quite a significant workload for someone that's part-time. So, so I don't obviously you work out ways to do it and everyone has their own ways of limiting their work life balance and different roles, so.
But I think that could be maybe I I don't know if there is a solution to it but that's just something to highlight. I think probably everyone's aware of.

**Thompson, Jemima** 45:36
Hmm. Yeah. Thanks, [SR012]. And it kind of yes, [SR004]?

**SR004** 45:43
Oh, no, you. You answer [SR012] first though.

**Thompson, Jemima** 45:45
Oh, I just was gonna, I suppose. Ask another question on top of what [SR012] was saying just about your workloads. And you know the hierarchy of, you know, where safety reporting sits in all other tasks and how that affects your, but I suppose like well-being in the workplace and how it makes you feel about how you're managing your, your workloads and so, [SR004], I don't feel you were gonna say anything related to that, but I'll let you speak.

**SR004** 46:15
[SR012], do you want to say anything on that first?

**SR012** 46:19
Well, I think as clinicians you're very used to sort of prioritising things according to urgency. So it's not an unusual situation to be in to sort of like flex things up and down according to how major a deal is, a reporting issue or safety issue is within a trial.
But yeah, I think, yeah, I don't know- I don't know what the solution is, but yeah, you do. You're very used to flexing up and down and expanding your time and prioritising things with your other workloads. So, I don't think that's that particularly novel or challenging for us. But I think I guess it's sort of like.
Yeah, acknowledging time devoted to trial activities and assessing that and sort of having room for, yeah, how that's managed in terms of contractual arrangements, things like that, sorry, it's a bit boring to talk about that, but I think that's something to think about because often we just do it because we have to. I don't know what any of the other conditions things.

**SR004** 47:27
I mean, I'm the same. So, I work part time and obviously always have one eye on my inbox for anything that comes in as related. So, one thing I've asked my trials to do is always like red flag anything if it's missing causality or related. So I know I definitely have to look at that SAE out of hours as opposed to waiting until my working day to look at it.
But I was just gonna say like, so I've worked in. I worked in a trial where in [trial name] when it was at its height of SUSAR and SAE reporting. And it kind of did feel a bit miserable because we were getting, I don't know, like 20-30 SAEs to review a week. So it was quite time consuming. And also, there's a lot of missing data as we've alluded to before, lots of things that didn't make sense or were filled in like incorrectly and. And also, I was like the backup for reviewing, re-reviewing other people's where they haven't re-reviewed it correctly the first time, so it did feel a little bit depressing when it was such a large proportion of my work, but now I'm working on trials where the- where there's like fewer events and it's fine now. I was just going to say as well that it's not just about the number of patients, it's about the years of follow up you're doing for them and also about the health of those patients. So, like following up patients with [disease], you're going to see more SAEs compared to maybe following up patients who've had curative cancer surgery and it's an adjuvant treatment, things like that. So, you need to factor all of those things in when thinking about how large a problem SAE reporting is going to be, not just numbers.

**Thompson, Jemima** 48:57
Hmm. Thanks [SR005]. No problem and thank you [SR004]. [SR006]?

**SR006** 49:20
I'm a bit like [SR009] in that we do very minimal- we don't get many adverse events, but when we did have a SUSAR come in, me and my fellow trial manager just hit the panic button and it felt chaotic and worrying and scary. And thankfully I think [trial name] were doing a reporting a SUSAR at the time so were able to watch their one. And now that I've done a couple, I can now if one came in, I wouldn't panic as much. Do you know what I mean? Like it does much, much more manageable. But I think that would be helpful if trial managers, generally data managers, whoever needs to do reporting are able to observe other trials that are doing reporting.
It's particularly if they've never had a SUSAR before, like, and I think that could be tracked within the trial. And whether the per trial manager wants to observe or not, because like I said, we were fortunate that [trial name] were reporting at the time anyway, but sometimes you might not have that situation. And you know, with the short timelines to report, you don't want it to be panicky. You want it to be, you know, smooth and calm and make to make sure you're doing the process right.

**Thompson, Jemima** 50:33
Yeah, I think that's a really good point, [SR006]. Does anyone have anything to add to this point about the kind of the safety reporting within your roles?
So I think I've got one more section that I want to do, but I just want to check that everyone is happy. Does anyone need a break or are we happy to continue?
Ploughing through to the end, we're all good. OK. I just wanna make sure everybody's OK. So because I know we've had a few people have had to disappear because it's that 3:00 o'clock meetings and school drop offs and all of those things. And, so yeah, just the last kind of thing I wanted to cover. It was just we've talked about some of the problems and I know we've kind of alluded to some of this, but I just wanted to get a feel for - are there any specific areas of safety reporting that you feel could be improved? And kind of how might we facilitate those? What might be some of the barriers? How can we measure how effective they are? Kind of all of those things? [SR008]?

**SR008** 52:23
So yeah, I can only complain about [trial name], but yeah, it seems like there's so many steps to just so initially you get the SAE form just through Galaxkey. That can also be an issue just to get that because you might get a site emailing can I have access to Galaxkey. So you've already encountered one issue and it's figuring out how to receive that form in a confidential way.
And then I mean [trial name]’s probably one of the only few trials now that uses macro and cactus. But as the processing team, there's two different, two additional steps that you have to do there to log the SAE before you even well, at that point you determine if it needs to go for prior to review to the if it's related or not, if you've got missing information. So, you've got all these different steps before it even gets fully entered into the database. So yeah, it's - I think OpenClinica sounds like it's a lot easier I think. But yeah, for [trial name], it's just to process one SAE you can take you up to half an hour, so.
If you're on the router for that day and you've got four or five SAEs, that's probably your whole morning or all afternoon just doing that. Yeah, but sometimes you get a perfect SAE and you don't have to, but that's rare.

**Thompson, Jemima** 53:35
Yeah. [SR012].

**SR012** 53:44
Yeah, I guess it's more the logistics and ease and quick speed of doing these things, working across four different trials, one using Cactus, 2 using open Clinica, 1 still using paper forms. Or some of them even having where you have to sort of edit a pdf and then save it and encrypt it and put it on Galaxkey, it's just so time consuming and it could be so much quicker, especially if you're working across lots of trials. If it was all just the same across all the trials. That's the dream life and then Galaxkey's nightmare. Like, honestly, I think everyone hates it. But. it's just we- logging into open clinical Galaxkey and my normal e-mail all the same time, just really hard to search for old emails, all that sort of thing. Anyway, that's my moan

**Thompson, Jemima** 54:30
[SR009]?

**SR009** 54:32
Yeah, I agree with [SR008] and [SR012], like we don't use Galaxkey, but we use Egress. But even with egress, if you wanna go back to an SAE report, the link doesn't work and you have to request a new link from Egress and that can take a while. And I think an issue that I have is sites don't - so we use we ask sites to use the CTCAE version 5 to classify event terms and I don't think they understand the concept of using that and they end up putting any random terms which you will have to then query with the site and sometimes it's ongoing even though, you know how SAEs you have like a criteria for it to meet an SAE and if you don't have an event term then technically that's not but so it's just like logistics. To, you know, for them to report an initial report, but yeah.


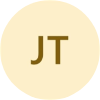
 **Thompson, Jemima** 55:31
Thanks [SR009]. [SR004]?

**SR004** 55:35
I was. I was just going to say, like, sort of backing up [SR009]’s points as well. One - a few years ago, we did an audit in [trial name] and the average number of times you need to go back and forth between site to get an SAE to completion was I think it was five times on average with a range of like 1 to 15 or something crazy. I was - but then I was the other thing I was wondering, and I was going to ask [SR012], have you - are there new problems reviewing SAEs on Open Clinica or do you find that
there's certain things which are no longer – so, for example, I frequently get the start date of the medication being given obviously wrong, like very recently when I know they've joined the trial a long time ago. Does having Open Clinica does that have ways of like checking some of that and does it prevent some mistakes or are there new issues that arise because of it? I'm just interested.

**SR012** 56:28
I do. I do like, Open Clinica for [trial names]. It is much more streamlined than sort of the old days of paper things. I mean everything it comes up with new issues. Again, it it's much slower to look at concomitant medications like you have to click into every single one and if there's like 50 then that drives you absolutely mad. But I think that I think net Open Clinica is easier than the other thing - systems I've used and it's got little things that are frustrating, but overall it's that it's good, easier to use apart from. I mean, if in in a trial there's new safety reporting that needs to be added in, for example in [trial names] we've had to have much more detailed pregnancy reporting then that's a whole separate paper form system that hasn't been built into Open Clinica and then I've got two systems I'm using. So that again new things come up as good systems going, but I think net Open Clinica has improved things. I find it much easier than cactus.

**Thompson, Jemima** 57:36
Yeah.

**SR006** 57:39
I'll just say what I said earlier. Mine is all just about trackers and if they can help streamline all of that, that would be great.

**Thompson, Jemima** 57:51
So I suppose, listening to all of this, and all of that kind of what some of these problems are, and they all seem like they're fairly in the same sort of area.
What do you think we could be doing to improve that? How can we make it better?

**SR006** 58:22
I think from a tracker sense would do you like just sharing the resources that people have already created and someone deciding, yeah, we'll take a bit of that bit of that and you know then sharing that again. Sorry [SR012], I got in there before you. **SR012** 58:42
That's alright. I was- I was just thinking I mean.
Yeah, I don't think I have enough insight into what the plan is for the CTU in terms of the different software that's being used. But if Open Clinica is being used across a lot of trials, then thinking of a subgroup that's looking at ways that shared experience and user experience improvement and optimising the software across trials over time within the in, within the unit. If I think about sort of the electronic patient records within the trusts we use like Epic that has a team that looks at optimising as you go along and then that's transferred across all trials and oh not all or specialties or whatever or or teams that are using it and maybe that I mean maybe that already exists, but like a way of shared experience and optimization going forward.

**Thompson, Jemima** 59:37
Oh [SR004].

**SR004** 59:39
I was just gonna say so in our last clinical science functional group, someone suggested that we like as clinicians have a meeting where we talk to each other about how we do safety reporting across trials and then seeing if we like compare and practise taking best practise. And I imagine that that happens at different levels, but maybe that could be something that happened like this statistical team could talk to each other. The DMS team working on different things and you could use that shared experience to come up with the best potential, best ways or solutions for problems people are having.

**Thompson, Jemima** 1:00:19
Hmm. And do you think that there? Because I'm just thinking 'cause. You're coming at it all from different perspectives. We've got clinicians and trial managers and I just wonder, and you've mentioned DMS as well and I just wonder, do you think that all of these kinds of different ways of looking at it are all joined together enough. Or do you think that there's too much distinction between, like the clinicians do this part and the trial and just do this? And DMS does this?

**SR006** 1:00:55
I think there's so much to cover in safety reporting that.
It's really important that you know exactly what your role is within it. I don't know if this is what you're indicating, but I don't - I don't, I think it'll be easier if I just know what the trial manager role is within the clinical review section and then the clinical reviewer knows what they need to do in that aspect. If you know what I mean, I don't think we need to know necessarily know what the whole picture looks like. Yes, but I don't think we necessarily need to know every single role independently, because there's already so much we need to do. **Thompson, Jemima** 1:01:31
Yeah.

**SR008** 1:01:36
Sorry, [SR006], I think you mentioned the safety group does that is there, are there different expertise in that group or is that just mainly just for reporting?

**SR006** 1:01:46
Yeah. So, the safety group has a few CPMs. It's got [name] who I think is like the PV officer now for the [CTU], it's got a few different expertise on it and they've recently brought me and [name] into it as trial managers to I think try and you know have a voice of making the decisions that they make or the discussions they're having actually be like translated into like the role that we would be doing as people who sort of take on the bulk of the reporting. So yeah, but I mean, I didn't know who was part of the safety group either before I joined it. And I've only been on two meetings recently. I think there's quite a lot of plans coming. Yeah.

**SR008** 1:02:31
Just thinking maybe. Well, [name]’s not here. Of course we can't speak for him, but that could be a root because for example, in the RGC you've got so many different roles and expertise that you tend to discuss quite, you can probably answer a lot of questions within that meeting. So yeah, maybe something to.

**SR006** 1:02:48
Yeah, the updates that I think they give are not always shared in like, kind of, what’s the word - that's what they did a lot of people sound the same group, have been in safety group for quite a long time. So, like the updates they're giving, I can't. I'm not always following what they are. But I think as I say, I think they're going to do some safety training for the MRC soon. So, I think we'll get all of the necessary updates from them.

**SR008** 1:03:02
OK. Yeah. That will probably help, yeah.

**SR006** 1:03:16
Yeah.

**SR004** 1:03:19
Do you know if the safety group ever get?
Feedback from the MHRA. Do they have a link to get advice on certain aspects? Sorry, I know you just started.

**SR006** 1:03:29
I don't know if they have - I don't know if it's necessarily advice like I think they're normally just speaking to the MHRA about what their like, new regulations mean for safety reporting. And like other CTUs. And I think they also speak to other CTUs academic CTUs about, like their process as well. So, I think there is a level of like communication and sharing between them. And, but like I said, I think they've updated the safety SOP recently. They've been updating quite a lot of the documents and the training’s coming. So, I think this is all being translated into like communication and training outwards now for the rest of the unit.

**Thompson, Jemima** 1:04:13
Thanks. So I've just got two more points that I wanna cover before I let you all be free. So just, in terms of these, you know, we've talked about the fact that there seems to be this quite a big variation in who's doing what and how long it takes and how much time it takes. Do you think that there is a measurable there is a a way to measure the efficiency of our safety reporting processes in like a meaningful way?

**SR006** 1:04:54
Could it be like how confident the trial team fills in their reporting procedures?


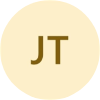
 **Thompson, Jemima** 1:05:05
Yeah, yeah, this all open to you? It's. Yeah.

**SR006** 1:05:07
Or how long it takes? Yeah. How long it takes to report a SUSAR once you've received it.

**Thompson, Jemima** 1:05:17
I'm interested in what you say about the confidence of the trial teams.
And how we measure that?

**SR006** 1:05:27
Would that just be their opinion on it? So like, you know from one to 10, how happy are you with this step this you know these different aspects of it?

And then, like, you know, after they've done training or you've done X amount of work, you can do the same assessment, can't you and see if that's generally improved?

**Thompson, Jemima** 1:05:48
OK, that's a really good point. Especially 'cause we talked earlier about training needs as well. So thanks, [SR006], [SR004] And then [SR012].

**SR004** 1:05:58
this is just like a very rough idea, you could do like a time in motion study from the point that you've received the first e-mail to when it's finally like all completed and just record every step of the way and how long it takes.
Obviously there's like then that's not taking into account the complexity of the patients and things like that. But if you did like say the 1st 10 SAEs on each trial and follow that up afterwards and that might give you an idea of some of the processes that are involved.

**Thompson, Jemima** 1:06:33
Do you think that sort of time on task is a problem or isn't in a like there are inefficiencies in terms of time spent on safety reporting tasks?

**SR004** 1:06:49
Nothing. I'm not sure. It's like the time on task but per say, but that would highlight how many times for example you need to go back to the site or if the clinicians are not answering you how many times you have to e-mail the clinician to ask them to review it and what's the delay at each step between you asking for something to be done and getting the answer. So, it might not tell you much about the CTU processes precisely, but it will give you an idea of what a burden the SAE processing whole procedure is on on a trial basis.
It's just one way looking at.

**Thompson, Jemima** 1:07:26
Yeah.

**SR012** 1:07:30
Yeah, I guess it's the same the same thing, but like sort of some figuring out some way of auditing intermittently within trials or between trials about time taken on to us or number of emails per task sort of scaled according to complexity of the events.
I mean, just subjectively, it will take me half an hour to edit a pdf for a [trial name] event and it'll take me 5 minutes to do it on Open Clinica just clicking the buttons that I need to click so.

That's I can tell you just from subjective that there's efficiencies that could be made in one trial compared to another. But I don't know how you'd formalise that.

**Thompson, Jemima** 1:08:08
Hmm. I just wonder as well. Like, do you, you know, do these things?
Get help you to get out the kind of root cause of some of these so so things like you know how long it takes you to click through a pdf is quite you know the time is clearly like the root cause of the issue. But I just wonder where things like why is it taking so long for a clinician at site to reply to my e-mail with a query when they know that they're supposed to do XY and Z? I suppose is that the thing that we need to be measuring or is it like, is that the actual cause of the issue?

**SR0012** 1:08:52
Well, I guess I mean you have the reports within trials, if you got multiple sites about their time like delays in reporting and compare between sites. And I think you can take that back to sort of TMGs and if there's a particular site that's performing less well, then you can cover in SIVs and things like that, but not SIVs- monitoring visits but.

Yeah, I think that would be the easiest way to do it, but I can't think of a different, more formal way because you don't add to their workload. You don't want to get them to have to do audits of how long it takes them to fill in a form because they're quaffing the site's international sites are so stretched anyway, and that's probably why they're got delays anyway. So, to add a step, it'll probably make everything delayed even more so, so it'll be. I think it'd be easier if it was monitored from our end rather than adding to the work.

**Thompson, Jemima** 1:09:43
Hmm. Thank you.

**SR004** 1:09:47
I was just going to say, I guess, like doing like a time in motion study. It's not going to tell you the 'cause. It will just tell you how bad the problem is.

If you need to go to the cause, you probably need to ask people that you need to look at where the hold ups are, where the bottlenecks are, and then go and speak to the people at each of those points to find out why there's a bottleneck at each of those points.

**Thompson, Jemima** 1:10:10
Yeah, that's a good point. These are all good points.
They don't have anything to add before I move on to the last little section.
OK. So my last thing, and maybe the most, I don't know, I'll leave you to decide what you think of it. But I suppose to what extent do you feel that the work that you will do in safety reporting is actually helping to maintain patient safety?

**SR004** 1:10:55
I mean I I've obviously like the SAE and the SUSAR system is a way to be safe about immediate problems and I do think that that is a way to keep people safe. It means that if there's unexpected side effects, they're picked up quickly and reported back to the MHRA very quickly.
It feels unlikely that some that that's you're going to get new safety signals in some of our trials, but that's sort of going back to the risk-based approach, right. It's probably slightly aside from this, but I feel like we spend literally hours and hours of site time collecting adverse events and trials and then the amount of effort that takes compared to how much of that data goes in the publication, it always feels like there's like a vast mismatch there. So, I don't know whether the answer is that we try and review how we collect that data. There's better ways of collecting it, more efficient ways of collecting it. But also I think we should report that data better because I think just having a table of worst grade experienced by patients and in some cases like only grade three to four or five events reported in that it's kind of doesn't really capture the patient experience of what being on those treatments is like. And we sort of just ignore all the grade one and Two's even though that might be making people miserable. So I think - I feel like our adverse event reporting and collection is like absolutely a behemoth for sites, it's such a big piece of work for them to do and I don't think it adds very much to the data we produce.

**Thompson, Jemima** 1:12:41
Thanks, [SR004]. It was a really nice comprehensive answer.
Anyone else have any thoughts on yeah, the work you do and it's kind of relationship with patient safety. How you interpret that? [SR012]?

**SR012** 1:13:03
I mean, I guess I having one little optimistic thing I think.
The learning, the shared learning across clinical sites with some of the trials that I've been on it I think is really valuable, right? If you're discussing complex cases and you open up to the clinicians or other specialties that are at each of the sites then then I think that can lead to continuing professional development for all the clinicians at the different sites and and I think that's really that's really that's not and that's not the aim of trials, but that's a just that's a benefit that can be really, really valuable.

**Thompson, Jemima** 1:13:43
Thank you. Anyone else have any thoughts on that?

**[SR008]** 1:13:58
I mean, I'm not a clinician, but it seems like for example, we have the [trial name] Clinical Safety Committee meetings on every three months. So, a lot of the SUSARs that we report for the standard of care hormone therapy, it always comes up that there might in fact almost all the time there's a SUSAR, it's reported for a fall, but everyone is all the clinicians are usually in agreement that it's already known that this is going to cause that. But obviously the investigator has put that as a probably related. So, I wonder if, because we've reported that as a SUSAR, will that at some point influence maybe the SmPCs for these for that particular hormone therapy? Yeah, because we do seem to report a lot of that, but.
As [SR004] and the team, they usually put a comment that it is known, but obviously investigator has reported it. We kind of have to report it as a SUSAR, so wonder if that will maybe at some point influence the RSI and SmPCs.

**Thompson, Jemima** 1:14:54
Hmm. That's interesting [SR008] and so is it, do you think it's, like, the relationship between like, do you do you feel like you understand the relationship between the work that we do at the CTUs and then what happens with the RSI and the IB’s or SmPCs?

**SR008** 1:15:17
Yeah, I think, some of it, I mean, of course we can't say. And so we have to report things that are not on our RSI or not on our expected terms, so.
Yeah, I think that's you could probably get a positive spin from that, just that the knowledge that we are giving because of what we've learnt from [Trial name] and that could maybe in the long run could influence future trials, just don't know.

**Thompson, Jemima** 1:15:45
Thank you. Anyone else have anything to add? OK. That being the case then, is there anything else that anyone wants to get off their chest about safety reporting processes? Because now's the time anything that I haven't covered or anything that you you think is important about the process and how we can improve it.
All of your burning desires have been met fantastic, in which case I'm going to just say thank you so much for being here today and for your participation. It's been really nice hearing all the different discussions, a different point of view on this issue, so I'm just going to stop sharing and I'll stop recording as well.

**Transcript Focus Group 3: 27-Feb-25**

27 February 2025, 02:11pm


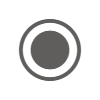
 **Interviewer** started transcription

**INTERVIEWER** 0:04
So yes, lovely, we've got recording, we've got transcription. Excellent. So, thank you very much everyone. So, I just want to start this discussion today with just some introductory opening questions. Just I want to find out, so that we're all working kind of you know from the same page, is what do you understand by the term safety reporting in the context of clinical trials?
What does it mean to you?

**S013** 0:37
That's funny. That's like an interview question, isn't it? It's like one of the first questions.

**S014** 0:43
Transparency. That's one word for you, transparency.

**S013** 0:43
You would, I would ask.

**INTERVIEWER** 0:46
It’s not a test, I promise.

**S013** 0:50
So it's just about ensuring that we know what we want to collect and ensuring that sites know what they need to collect and what we need to be doing to meet the required regulations in terms of what needs to be reported safety wise. You know the different in sort of stuff that needs expedited stuff that doesn't how things are triaged is quite broad term really isn't it.

**S015** 1:21
Yeah, I guess they have to define in their protocol what they want to report.
And we just make sure that they adhere to that, but also follow the guidelines that like the regulatory requirements.

**S016** 1:35
So it's all. It's also ensuring that what we collect is adequate to make sure that we're maintaining patient safety for the trial to make sure that we're keeping an eye on what kind of reactions that we're getting, adverse reactions etcetera to make sure that the trial is actually safe. You know, that's one of the main things I would say as well as all the regulatory things. It's making sure the trial you're running is actually safe for the participants.

**S015** 2:02
Yeah. And they use the up-to-date reference safety information.
I need to make sure that they're like keeping on top of that.

**INTERVIEWER** 2:02
Thanks for that. S017?

**S017** 2:16
Yeah, I guess just same thing in a different way. I think it's the data that we need and when we and defining when we need it to keep our patients safe and comply with our regulatory reporting requirements.

**INTERVIEWER** 2:32
Thank you, everyone. So, we're all kind of on the same page there. And so that kind of leads me into asking about what some of the main tasks and processes constitute safety reporting and particularly in the context of a CTIMP trial, which is kind of our focus today.
Are there any, you know, how would you define those different tasks and processes?
S018.

**S018** 3:05
I think building off of a lot of what everyone just said there.
There are the kind of preparatory tasks, so the considering your risks doing your risk assessment, writing what your requirements are in your protocol, that's kind of everything to get ready for safety reporting. And then there's the act of safety reporting itself, so ensuring that you've got a process for sites or whoever's responsible for reporting on the safety of the participants to the CTU and or the sponsor and then a process for the internal management escalation and if necessary, expedited reporting of those events. That's it.

**S017** 3:52
That was an excellent interview question answer.

**S018** 3:56
Do I get the job?

**INTERVIEWER** 4:00
Everyone's doing excellently well done. S014?

**S014** 4:06
So to an extent, I would imagine that some of the safety would be considered in the risk assessment at the beginning of the trial because that’s going to constitute what it is that we will be reporting on and what level of detail. So, it becomes about associating where those risks are really in terms of the validity of the data in its own right.

**INTERVIEWER** 4:35
S019.

**S019** 4:37
I think the answers that were given were really accurate. The preparation, the monitoring, the reporting. I also think training is a huge thing that falls into that, ensuring that, for example, from a CTU perspective, that sites understand those requirements and are following through as per protocol and then we're monitoring to make sure that that's happened. I think that forms a big part of it.

**INTERVIEWER** 5:08
S016.

**S016** 5:09
So, it's also a large part of it is making sure you have systems that are adequate to carry out the safety report, so the eCRF systems are adequate, you know to make sure you capture every aspect of kind of the SAE reporting that you need to do so, for example, you know sign off by PI’s, additional review, making sure you've documented everything correctly and the ability to report on that so that you can ensure that you've done it correctly within a regulatory time frame.
I mean that's a large part of what I do is trying to have systems in place to make sure we can do it for all trials. But on top of all the other things that everyone said as well.

**INTERVIEWER** 5:51
Thank you, S016. S015.

**S015** 5:55
Yeah. And we also need to make sure that we follow it up. So, they might initially report it, but it hasn't, the event hasn't finished. And so we need to make a log and make a note that we need to follow it up to when you get we need to get the site to follow it up to resolution, which is particularly important towards the end of the trial because the events might still be ongoing at the end of the trial, and then depending on what they are, it could be that they have to be followed up to completion.

**INTERVIEWER** 6:29
Thanks, S015 and S013. And then over to S017.

**S013** 6:34
Yeah, I was just going to say, like, going back to the very beginning, it's like getting PV engaged very early on into studies is important when it comes to things like we work a lot with pharma companies. So, it's having those early discussions with them about contracts, about writing the protocol, reviewing IB’s just to make sure that we're collecting only what we really need to collect safety reporting wise and we're not collecting anything unnecessary and also agreeing what we're going to do throughout the study when dealing with these pharma companies, because sometimes they can be asking for far too much. So, I think it's important to have those discussions earlier on to agree how what information we're going to share with them and when.

**S017** 7:14
Everything that everybody's already said, I just was thinking of one more thing to add. I think probably reference safety information management is a big part of it and I think a part that is overly, hugely bureaucratic and resource intensive unnecessarily for us as academic triallists. It's hugely important for those manufacturing IMPs, but the kind of current process of managing that and the need to submit updates to substantial amendments and things like that is something that we were really hoping that in the update to the regulations we wouldn't need to do. But it doesn't look like that's going to go away. So, I think that's, just as we're in a focus group and it's about kind of collecting things, I think that that it takes a lot of time, probably that could be saved for us as academic triallists.

**INTERVIEWER** 7:58
Thank you, S017. And you know any, honestly any opinions and thoughts that you have are super important and you know I want to hear everything and a lot of the things that you've touched on so far are things I have questions about. So, it feels like we're kind of going in the in the same direction and everybody's kind of thinking the same sorts of things and we're all on the same page. So, before I move on, does anybody have anything else they want to add when it comes to kind of the processes and things like that at this stage?
OK, so I've kind of got something's gone wonky with my slides. I don't know why. OK so.
My next question was actually supposed to be asking about more generally, what impact do you think safety reporting processes for CTIMPs in their current form have on the kind of development and conduct of our trials? So, it's quite a big question, but I will filter down later. S019?

**S019** 9:11
I think it's an interesting question because we're kind of in the landscape of the MHRA, particularly pushing pragmatism. Which you know, we've not seen so much before. But in reality, at least in my experience, that translating through on the ground and what's being approved is very different. So just to give an example, we've recently put through a trial where we've said we don't want to report all AEs, only SAEs, and that's based on it being a type A trial, it's very low risk. There's lots of AEs within this disease group. We don't want to over report. We don't think it will be helpful. We won't be applying for an MA because it's a supplement and they've come back and fought against that, which it's not pragmatic and it creates difficulties with safety reporting the resource that then that has on the site teams who are delivering the trial, when what we're trying to do is make the research as easy as possible to deliver so we can get these medications out in the world to the people who need it. I think that the bureaucracy, I think is my main point there.

**INTERVIEWER** 10:25
Thank you, S019. I've got S018 up next.

**S018** 10:30
Yeah, I'd actually like to firstly wholeheartedly agree with and second everything that S019’s just mentioned there. I wonder, I was going to say something, it's slightly along these lines. I wonder if you could because it's not on the screen. If you could just repeat the question just to make sure I am going to answer that question. Oh, there it is.
Yeah, I think I would go back to the bit about the being able to take the risk proportionate approaches and that actually becoming embedded in practise, I think there's still hesitation on that front from Sponsors and some Chief Investigators, and I think CTUs are perhaps a bit ahead of the game on this sort of thing, trying to take these risks proportionate approaches.
And so it's often us suggesting it to other people like me as the Trial Manager, suggesting it to other people. But it's perhaps not something that's being thought of beforehand, like at the at the sort, maybe the grant writing stage or anything like that. So, it kind of leads to lots of things being done differently, across very similar trials, either within a clinical trials unit or across different trials, you could be running an almost identical trial and be doing your safety reporting very differently. Somebody could be doing absolutely everything and some people could be collecting and reporting very, very little or expediting the reporting of very little, for example. So, I think it's that uncertainty around what you can do. And I think then the different ways that the MHRA deal with you. So, S019's just given an example of push back on taking a proportionate approach, whereas we've just taken a very similar proportionate approach for another type A CTIMP and we didn't get any, we didn't get any pushback on that from the MHRA, other things that came completely out of the blue, but not but not that. So, I think it's very much kind of who you who your application lands with, affects what really happens to, what they decide to do, but I know you've asked about, I'm sorry, I'll finish my comment in a second, but I know you've asked about CTIMPs specifically, but I actually think it's also even harder for non-CTIMPs. The lack of guidance around what you can and can't do there.
is, does make it actually more challenging. I feel like it's so much easier to actually know what to do in CTIMPs there's almost like there is literally a guidebook for them, whereas I think there isn't for non-CTIMPs and I don't think it's appreciated actually, how much more difficult it is to run things like complex intervention trials than it is drug trials.

**INTERVIEWER** 13:34
Thank you, S018. And just to your point on the non-CTIMPs, this is why I've kind of I'm focusing on CTIMPs here and I actually want to focus on non-CTIMPs later on because I kind of, you know, later down the project, because I think you're right there that there is a distinction and that, yeah, there is a difference with non-CTIMPs and there are a different set of challenges, I think, to address. So, I appreciate you raising that because I think, yeah, there it is important and it's something that also needs to be considered. And as you say with CTIMPs, we do kind of have a bit more of a guidebook from the regulators about what they want from us.
So it's probably enough of me talking, so I'm going to move on to S014. I have next on my list.

**S014** 14:20
So, something relatively simple that we are trying to bring in is to have the full access to the some of the documents in a protected form. So, at the moment we request from PV, what are the SAEs for this monitoring visit we're about to go on.
But because it's not on SharePoint, it's not a document that's available. So, the efficiency of being able to go in and be able to do something live whilst on site and be able to help understand when they need to delve into issues or whether actually all the correspondence is happening straight with PV, you need to stay well out of this sort of a briefing meetings going on before and afterwards, and basic access like that. I think there's some work to be had there.

But I also think we are working quite closely with a risk-based monitoring working party within our unit and that's to do with ensuring that we're not overlapping with some of the checks that we're doing. So, the checks that are being done centrally by our data teams, we don't need to be double checking and triple checking at site. It's all being done somewhere else, but it's that permission to be able to stand back and think actually we don't do that, that’s not in our remit. This is what's been agreed, particularly for this trial, that's sat with them and that's- and I think there's a tendency to try and provide like a gold standard in a more commercial kind of field, particularly for early phase trials, when actually the work is being done. It's just understanding that and having that documented somewhere. So DMPs, TMPs, trial schedule, which document am I looking for this activity on and that can be quite confusing.

**INTERVIEWER** 16:04
Thanks, S014. Yeah, definitely. Where to find information can certainly be challenging. I know that when I was trial managing, it was definitely something that I struggled with and because it's all well and, wanting to know the information and knowing what exists. But even trying to find it sometimes can be a bit of a hindrance. Thank you. S016.

**S016** 16:24
So I've been finding the opposite really in terms of risk adaptation for the MHRA and that I tend to get it through quite successfully. The vast majority of the time. I mean, I've just put through a phase three where I'm not collecting any SAEs whatsoever, for example.
Or any AEs, because the drugs been in use for so long that collecting them adds nothing to the drug safety profile, and none of our endpoints so are related to the safety of the drug. We know how safe it is. It's been in use for forever.
But what I'm finding at the moment is we're getting a lot of conflicting information back from the MHRA. Often, so we've had conflicting information around RSIs and what is acceptable as an RSI, particularly with reference to SmPCs, rather than using the IBs. We've had IBs where we've complied with the guidance that we need to be complying with in terms of the CTFG and preparing IBs and they've been knocked back despite complying with them completely. We've had some very funny non-acceptances around- wantonness around CI review and wanting that to be removed, for example, from the Pharmacovigilance process of CI review of causality. We got that completely - just take it out of your protocol was one of our, because they didn't want it done.
And also, I mean, generally there's always this thing of you've got to balance what you need to collect in terms of safety reporting in terms of what's needed to monitor safety of the drug, for example.
So, you know, I'm trying to think of example, so I've got trials of [disease] with the drug itself is actually [IV drug]. And in and of itself, it's not particularly, we have no concerns about it from a safety perspective. The drug itself doesn't cause anything. We, from a quick look at our data, 2 out of 1000 SAEs are related to drug and they're dubious. When you go back to the site and just kind of go, you know, “are you sure?”
You've got to balance that against the need to collect SAEs in order to see if the intervention itself is causing any issues. So, there's this thing of like, especially with cardiovascular disease, the disease process is quite slow, and so what we tend to find is that in we get this global CIs wanting to collect SAEs for everything.
So, any [disease] admission, for example, I don't know if you ever worked on a cardiovascular trial, but even a small trial, these patients are in and out of the hospital like every 5 seconds, and especially in heart failure trials.

So, we've had a lot of issues with the CIs wanting to collect all this information so that it can go to endpoint type committees. Whereas in reality, it's not particularly, you don't really need to do that via SAEs, you can do it via other means by a follow up et cetera. And it, essentially it adds burden to the particular pharmacovigilance department, which is consists of me and half another person.
And when we're receiving 2000 + SAEs where only two of those events have ever been related to drug. And so, it's a balance of collecting events, safety reporting for drug safety and reporting to MHRA versus collecting SAEs for endpoints.
Which is, it doesn't affect many trials as much as it does cardiovascular trials, but it's hard to balance the two. And so, it makes cardiovascular trials very expensive due to this need to collect so many- so much data that's actually not all entirely required or relevant. I mean I knew the outcome of that trial about a year into it, because I'd bene collecting SAEs and endpoint data and drug safety data so I could I knew exactly what drug was better. Which I probably shouldn't.

**INTERVIEWER** 20:54
Thank you very much, S016, really well explained and yes, there are definitely differences among disease areas as well and that's really interesting to hear you bring that up. S013.

**S013** 21:08
Hello. I just had a couple of things to add and one of the things for us I feel that is an issue, and can have an effect in the trial is the amount of duplication. We feel that we're doing sometimes, especially going back to studies where we've got pharma companies involved, and we do a lot, we're all oncology and we've got multiple drugs, multiple pharma companies and we're sending the same SAEs to multiple people several times. Then we're sending line listings, then we're sending our DSURs. So, I just feel as such a lot of duplication of information that we're sending and we’re recording and we actually had an audit and that's what our auditor commented on was duplication that we're doing.

And the other thing that I think is important is, I think somebody touched this already, was training, but not just the site training, but CI training and PI training and making sure they understand the process for using your RSI for safety reporting. Because I don't think they do like, for example, knowing what's expected using an IB versus an SmPC and why for some trials you have to use your IB, you can't use an SmPC as your reference safety information. So, it's just I think training from everybody from a CI right through to a site is really important.

**INTERVIEWER**22:23
Thank you, S013. And yeah, training has come up a couple of times and it's actually something that I had some specific questions about later as well. So hopefully we'll be able to come back to this and find out a bit more about your thoughts on all of that. S018, I had you next.

**S018** 22:39
Yeah. I just wanted to pick up on the conduct end of things because my last comment was more on the development side, and talk about the impact that safety reporting has on the conduct for a trial from the point of the Trial Managers to in our CTU. We’re quite small CTU and we don't have a pharmacovigilance team or anyone like that. So all of the safety reporting whether it be AEs, SAEs and any version of an SAE is all handled all handled by the Trial Manager.
And it does create a lot of work if we don't get to take, or unable to take sort of a risk proportionate approach, and even if it is quite a risk proportionate approach, it can still involve a lot of additional work on top of all of your normal sort of duties. And I think the difficulty with SAEs as opposed to a lot of the other work that Trial Manager does is the unpredictability of them of when you'll be alerted to one. So, you can be happily working away, going through your current To Do List by order of priority that you've got it in and then suddenly bam an SAE comes in and it sort of throws your whole list off.

And if it might be that that sometimes they come in threes, and you'll suddenly get like 3 SAEs come in when you may not have had some for weeks. And then all of a sudden everything else is sort of like thrown out and you're just doing that. And then there's so much time spent chasing up the site for the missing data and the Research Nurses, Research Coordinators and things at sites are normally quite good at responding, but it's getting hold of the PI's to get them to do their bits, their assessments is quite is challenging. And so then all of a sudden you spend all of your time doing that and then it does involve a lot of kind of cover arrangements as well that I've not, before I was a Trial Manager, so just worked in normal research and you didn't have to find someone to cover you when you were on holiday and think I know it's just, it sounds like really trivial things, but it's just an extra layer of stress and complexity. Whenever you think you want to take any time off, you have to make sure that someone can cover your trial for SAEs, and that they're trained to do so, that they're going to know which ones need expedited reporting that they know who to send it off to. And obviously we have all of this written out in working instructions and things, but it's just kind of making sure they're aware. So, it's yeah, it's just like that extra level of work when you when it's sort of just you on the trial who's doing it.

**INTERVIEWER** 25:26
Thanks, S018. And yeah, definitely the bit about you have your To Do List and then it all goes out the window just reminded me of what it was like to be a Trial Manager.
S019.

**S019** 25:39
Again, I completely agree with everything S018's just said. It's, I think there's movement towards trying to reduce some burden. So, you know, we have seen some changes to the DSUR and maybe not needing line listings for that in the changes and removal of the progress report for the REC. So, you know, all of that is very resource intensive for Trial Managers. So at least there's some steps in that direction. But what I did want to say is I know that we've mainly been talking about AEs, but just to touch on pregnancy and safety reporting.
I think that, you know, we've talked about that on the surface, we're kind of talking about pragmatism and aiming towards that. But I still think in reality, as we've said, there's still a very cautious and risk averse approach, which often leads then to pregnant persons being excluded from trials potentially when they don't need to be. And then that has implications from an EDI perspective as to then we're not treating populations equally and getting that information. So, for example, I'm not sure you know, like supplement studies, things like that, where people can access this normally and should we actually be taking that approach or not?

**INTERVIEWER** 27:00
Thank you very much, S019. S016.

**S016** 27:05
It's just added to the DSUR thing that you just mentioned, S019. So, I think the proposal for a DSUR is bringing it more in line with the one that you report for devices, in which case it isn't any better, I'm afraid. It removes the need for line listings, but what it does add is the need to do a literature review and to write risk mitigation strategies and to write a full kind of, like, description of what's happened in your trial.
So it reduces the burden data wise to a point, but kind of adds additional work around the interpreting your safety data to a point. So, I would, yeah, I'm not terribly excited about that one yet. Hopefully it doesn't involve that. But from what I've been involved in, I think it is going to be more like what they ask for device trials.
So just as a warning there really.

**S019** 27:59
You're not making me feel very optimistic, S016.

**S016** 28:02
Well, you know, at least it's still yearly. Device trials are quarterly, so it's better than that.

**INTERVIEWER** 28:14
Thanks everyone and S018's just added in the chat as well that she agrees. We just managed to negotiate on one of our trials collecting pregnancy details, but not doing any follow up or other onward reporting on that. So, thank you everyone for your input on that.

And I think actually so lots of the things that we've touched on were things that I had questions about. So, we might be able to dig a little bit deeper and I think we kind of already touched on, you know, kind of what we could be improving and where the inefficiencies might lie, and what works and what doesn't. So, does anyone that have anything else they wanted to add on this point before we move on?

**S016** 28:57
On the second point, I think there's we do need more clarity from the MHRA to a point on some of these aspects. So, we have endless discussions in [location CTU] around IB’s and SmPCs and what the difference is between them in terms of when you use each one as an RSI. And although there's very strict guidance for IB with the CTFG guidance etcetera, but very little of the CTFG guidance is applicable to SmPCs, for example.
And the bulk of what we use for most of our trials is SmPCs and the guidance there is far from clear. There's good EMA guidance, but who knows what we're following. I mean with, with respect to the EMA. And so that that's one thing where I think the MHRA could do with a lot of clarity and also that as I've mentioned several times this this recent push back around having CI review of things which a few of us have seen at CTUs, it's I can get why, I can understand their point. But what do they actually want us to do?
Historically, most people have used CI review as kind of a backup. If they don't want us to do that and I can, and again, I can see why they don't, but it does add an extra layer of caution. Possibly over caution. But you know they give advice and they put out via blogs and they put out via their website, but there's no guidance that's actually useful.

I mean, I don't know if anyone's been involved in writing IBs, but that's a particular horror show, in terms of MHRA. They give you, they give you strict guidance, you follow it, they knock it back and you end up just scratching your head and wondering what you can do given that you've followed everything to the letter.

So yeah, I mean that’s where I think most and also the whole thing about legislation at the moment. It's very difficult to know what you're following, at points. I mean medical devices we’ll probably not talk about here so much, but the MHRA guidance on medical devices is verging on comedy at points. Since several bits it says you refer to the UK law and then you read the UK law and it says go and refer to the EU law.
But it's the EU, it's the old EU regulation, not the one that's currently in practise.
And so I think there is a bit of a need for the MHRA to clarify a lot of points around what they're actually doing at the moment, particularly with respect to the recent increase in GNAs that we're getting, most of which seems to be quite random, so arbitrary.

**INTERVIEWER** 32:01
Thanks, S016. And I can see like S019's nodding. Feeling the pain. Thank you. S015.

**S015** 32:09
Yeah, I was going to agree about the MHRA being inconsistent. Depends who you speak to as to what answer you get. But we're thinking of doing, so at the minute we have it that the site teams have to put like causality and expectedness and then when it comes into the central team, so we do a check of it, but we're actually going to make it so that the Sponsor, so our team, has to complete the expectedness assessment because that is what obviously taken from the RSI. So, that is clear, you don't have to have medical background training to be able to make the expectedness assessment and we think it might alleviate a lot of the complications if we do it centrally.

**S016** 33:00
So we dropped that ages ago. So, we don't have any assessment by site of expectedness. We do expectedness as the sponsor. We sometimes involve the CI but primarily, well, when I say we, I mean me. So it's kind of, yeah. So, we do it all centrally and it has solved a lot of problems because there's this whole thing of with investigators around, they think expectedness is did you expect it, clinically, did you expect it? Which is always a problem.

**S015** 33:22
Yeah, we're thinking.

**S016** 33:29
But I'd recommend moving expectedness away from everyone except sponsor, personally. It's worked very nicely for us.

**S015** 33:45
The other improvement I think we could have is better technology to be able to do it because at the minute we're mainly still on paper. So, it's a paper SAE report that goes out to the site teams and they fill it in and scan it and send it back. But if we could do everything electronically, that would improve our improve things dramatically I think and improve timelines probably.

**INTERVIEWER** 34:13
Thank you very much, S015, S018.

**S018** 34:20
Yeah, just picking up on that point, I wanted to touch on your what works well section and mention that I think the move towards electronic methods of reporting and processing SAEs has actually made things a lot better. It's a lot easier, I think, to manage a safety report or any data, whether that be adverse event data or SAE data when it's coming into you electronically, because first of all, you don't have to manage the handwriting issue. I mean, I'm sure we've all seen SAEs in the past where we actually can't read what's actually happened, but.
And then obviously updates and edits to that information with the whole cross it out, put your initials and the date and everything, and eventually by the end of a report you just end up with this awful looking form. So, we have moved completely online now for our SAEs in [CTU location] and that has helped a lot. And we're, I was also going to say about the expectedness, we are dropping that as a site responsibility and putting it now as a as a central responsibility for all of our trials. And I think that that's one of the most annoying queries I've had to deal with in the past is when you know that the local PI has put that something’s either expected or unexpected, and it's the complete opposite to what we know it is. And you're obviously you're not allowed to actually tell them what they should report to you. But you kind of have to suggest to them that we might want to change it because actually you know it's wrong and so, yeah, we're perhaps a little bit late to the party, but we are bringing that in as a central process now. And I think that that will really will really, really help.

**INTERVIEWER** 36:14
Thank you very much, S018. S014.

**S014** 36:20
Something that one of our lead monitors has identified is that sites have said to us, oh, you're not very consistent as CTUs about what information you collect here at sites. And this was just in response to a couple of comments ago, when you're saying you know it's all done centrally, clinicians don't do this. And I'd just be really interested in that process that you've done, because I think obviously sites are finding our AE logs or AE forms or forms or that the collection this information quite frustrating.
And it's really what sort of mitigations that we could bring in ourselves to alleviate the work that they're having to do if they're feeling it is due in duplication and are they best person placed to do this. I think that was a it is something that's been brought up time and again and I don't really know where to start with that. So, I’d be interested to hear more about the people who have been doing that.

**S016** 37:27
I'm sorry. You mean in terms of reducing the burden on sites to a point, so we.

**S014** 37:30
Yeah. In terms of the documentation of all of these, you know, what are you going to clarify this, You know, it's -

**S016** 37:40
I mean, the main thing we've had with it, the main look we've had of it is it is in risk adaptation to be honest. So, you know, for all of our low-risk trials, we basically don't collect anything. We very rarely collect AEs and if we do, it's a subset. We're trying to limit what we collect from AEs. We reduce SAEs down to the bare minimum if we can.

**S014** 37:59
OK.

**S016** 38:05
Everything is done electronically, from the review to the you know.
And then the only other and then, yeah. And then so I'm just trying to think of example. So, like we've got a couple of acute trials where it's basically like acute sepsis for example, where there's so much going on that you can't possibly tell whether something is due to drug or the sepsis or anything else that's going on or the myriad of drugs that they've had inserted into veins since they appeared. And so, we're basically just collecting nothing.
We're just, we've just written a big, elaborate thing in the protocol saying this is too complicated, there’s so many confounders nobody could possibly tell whether this is due to one [drug] over another [drug], or whether it's, you know, whatever, whether it's the sepsis. And so, we've literally just restricted it down to what would be the distinguishing characteristics of a serious adverse reaction for the IMP that would that not be present in any of the other medical conditions that they've, that are concurrently trying to kill them at the same time and myriad of drugs. So, what would be unique to that? And then we've limited the safety reporting to that. for example. And that seems quite popular with sites.

**S014** 39:23
You're getting that one through.

**S016** 39:23
Yeah. And also, this thing of removing the collection of endpoint data and things like this. You know, once we have patients come off drug, we don't collect SAEs anymore, we stop. We're not doing it then we just stop dead and then and shift over to collecting events via follow up, for example if hospitalisation is one of your endpoints but it's being collected via SAEs for the bulk of the, while they're on drug, the minute they put in a stop or they tell us that they stopped treatment. All that for all those hospitalizations then shift over into other bits of the eCRF, again to try and reduce burden.

**S014** 40:05
I think that's the point isn’t it, is to continually adapt to the risk associated with the trial at the time not to be the risks associated at the beginning of the trial.
Yeah.
**S016** 40:18
Yeah, I mean I’ve done that as well. I’ve also adapted trials on the fly, as I’ve been going through, you know. So, we've seen, you know, one out of we've had two out of 1000 SAEs have been due to drug. Right, let's limit the, what we collect.
And then we limited it, and then even then we would still find that we weren't getting anything. So, we reduced it even further. And yeah, so it's just it’s whatever you can do to make it easier, I mean, I’ve done every job there is in clinical trials, so I know how horrible it is to do all these things so.

**S014** 40:48
OK. Thank you.

**INTERVIEWER** 40:50
Thank you S016 and S014. S015. Sorry. You're muted, S015.

**S015** 41:05
Sorry, I was going to say in the protocol you could design it so that you list the adverse events that you don't expect to be reported and also the adverse events you do want reporting such as the adverse events of special interest and like say how they need to be reported. And also define when you want to start and stop collecting the adverse events. So, it could be from consent. Or it could be from when you have them starting the actual IMP.
And it should also list when they should stop reporting adverse events. So, depending on the drug, it could be that it has a long period after they finish treatment or if the drugs like not so severe, then you could like stop reporting, like not long after they finish treatment. So, the duration, the design of the drug or the activity of the drug would like, when you design the protocol you need to think about those things.
And also you could design it such that the adverse events don't have to be reported by the site team, they could be picked up by the National databases. So, you could get data in from the national database as instead of having the site team follow the patients that get them followed up by extracting data from NHS digital centrally.

**INTERVIEWER** 42:38
Thank you, S015. S018.

**S018** 42:43
I don’t know if this is relevant actually to any of your specific questions here, or whether it'll come up later, but one of the things that I always find a bit confusing isn't about thinking about safety events that are expected or not expected of the drug, but of the absence of drug, which kind of tends not to get covered. Or maybe I've not done my research enough, but I've done a few trials now where marketed drugs are routinely given by clinicians to patients for a condition that they're not technically Licenced for or there's no evidence basis, this whole history of a lot of medicine being done without necessarily being evidence based. And we're in a period now where we are trying to obviously collect evidence for all the things that have been probably being done for decades. So you know patients with X condition are given a steroid or some other kind of regular generic, quite cheap medication without really there ever being any evidence that it works and it's not in the sort of the indications for this medication.
So, we do a lot of trials now of these marketed drugs to see whether they are actually effective and whether they should be being prescribed for these conditions. So, they're often placebo-controlled trials where half the people get the drug that this clinician may ordinarily prescribe and half the people will get a placebo and they won't get any active drug. And then it's the worry then of even though there's no evidence base for giving this drug, would it be harmful for the placebo group who aren't getting it and people perceive these trials, you know, you get more problems with the Ethics Committee and your PPI group and various things like that of the idea of not treating people. That's sort of more the worry and it's not giving the IMP, which you know, probably has this vast safety profile. It's not giving the active IMP and how do you, when you're doing your expectedness assessments and your causality and all of those things, it's all to do with it being related to or expected of the actual drug itself, but there's not much around the impact of the absence of that drug. I suppose if it's a blinded trial, you don't actually know if they're on the active drug or not, you assess it as if they are but, yeah, I always find that it's quite a challenge with placebo-controlled trials, whether it's a perception of withholding treatment rather than it being a brand new drug that's never been tested, and you, you are literally testing a brand new thing against, you know, the absence of an existing treatment, which is very different situation.

**S016** 45:50
Is that not what you'd use an IDMC for though?
That's what the IDMCs are for. To compare event rates outside of SAE report. SAE reporting is not really there to pick up differences if there's no drug but your IDMC will be looking at rates of hospitalisations etcetera and rates or should be looking at rates of hospitalizations between arms and comparing to what they would expect as a clinician to see if there's a difference between-

**S018** 45:55
Yeah. That is. Yeah.

**S016** 46:20
the baseline patient group and the patients receiving placebo, for example. I mean generally they tend to do better because given the placebo always has a massive effect on patients, they tend to do better than baseline as it is. But that's what your IDMC should be looking for is differences between not being in a trial entirely in their clinical experience and the event rates happening in that placebo group for example.

**S018** 46:48
Yeah. So that that's fine. That is what we have committees do during a trial. I guess in talking about in preparation for that trial in terms of working out what level of proportionate approach you're going to take to the collection and assessment of that sort of data. Because you might think oh, we're doing a really, really safe drug, it's been around forever, we’ll hardly capture anything in terms of safety reporting, but then you might be missing out on things related to not being on the drug then, in that in that case, which is something to.

**S016** 47:25
But in general, you would still collect endpoint type data though in that case. So, you can then compare endpoints to see if there's a difference between arms, even though you're not collecting drug safety data, you would still collect something you would usually still collect, for example, hospitalizations and deaths and things like that. But I mean, we always do sorry, so.

**S018** 47:42
In some cases, yeah, I have had some trials where they haven't had safety or hospitalizations as an end point in these cases. So again, it really varies. And there's a Trial Manager you're often not in control of it of these decisions.

**S016** 48:01
OK. I mean, what we always build things up and to have a look for differences between arms.
I don't know. I just always assumed everyone does, to be honest.

**S018** 48:11
Yeah, I suppose it comes back to that variability, doesn't it? With not always definitive guidance or very clear things guiding us. We are all doing different things that we probably all have our, we'd probably all write a very different protocol for the exact same trial, which is where things get difficult, I think. But yeah, we do have IDMCs check out our unblinded data for where it's relevant.

**INTERVIEWER** 48:41
Thanks, S018. Really interesting discussion there. S015, you’ve been waiting patiently.

**S015** 48:48
That's OK. The placebo trials are interesting, as you say.
So we've had trials where some sites won't take them because they think that the patient should be on some sort of treatment. So, they won't take the trial because one of the arm was placebo. So, and then we've had other cases actually where the patient were randomised and because they were randomised, it wasn't on, it wasn't blinded, when they were randomised, they were randomised to placebo and then patient decided they wanted to withdraw because they were wanting to go on the other arm.

**S014** 49:25
Mm hmm mm hmm.

**S015** 49:26
So yeah, so that's not brilliant.

But the other thing that we can do as well is in the protocol, design it so that you don't have to collect the events if they're related to the patient's background history, so or disease area. So, in cancer for example, patients have a lot of adverse, of like particularly if it's if they're like towards the end of their life, if it's like a late-stage cancer. You don't want to be collecting all the adverse events that could, would be related to their cancer, because otherwise you're going to have lots of reporting. So, you build that into the protocol to design it such that you don't have to collect those events.

**INTERVIEWER** 50:22
Thank you very much, S015 and everybody.
I'm really aware of the time and I've just been kind of watching these discussions and it's yeah, there's so much to dig into that. I'd like to dig into more. And but I am going to just move us on slightly because I know we've actually already been talking about this a lot, which is why I was, I'm just going to let people talk about regulatory and pharmaceutical guidelines and the things that we are expected to do. And for those that are working alongside pharmaceutical partners kind of, a little bit more about all of that and we have been talking about risk adapted approaches which I want to dig into again in a minute. But I wanted to start this section now by talking about SUSARs specifically. So we've been talking about SAE and AE reporting quite a lot generally, but I wanted to dig in a little bit more into those processes.
So we know that SUSAR reporting is important and it's a requirement.
And I just wondered kind of, what your opinions are on those processes for reporting suicides and the portals that we use and things like that go for it, talk SUSARs. Yes, S019.

**S019** 51:44
It's a bit quick, wasn't it? I think my comment it really comes back to training. So, I know we've talked about training site teams, CIs, PIs. We found from a CTU perspective, sometimes that does involve the sponsor. So, if you've been delegated, you know safety reporting by the sponsor, usually the sponsor will retain this responsibility for onward reporting of SUSARs. I've come across it quite a few times where the sponsors, don't quite understand this requirement or haven't set up on the portals. You know, we've had a SUSAR reported and they don't have an ICSR login and that's happened at the point of the SUSAR being reported, so that gets a little bit frustrating sometimes. You know, I think it's because at least when the trials have been involved with the SUSARs have not been very common and have not happened very frequently.
But yeah, I would say, clear delegation of duties and making sure that all of the parties are aware and trained on how to do that, is something that's come up for me.

**INTERVIEWER** 52:54
Thanks, S019 and S018. Don't worry. It's interesting you've only reported one SUSAR. I did lots of SUSAR reporting, but we were an international trial that I was running as well. And that was chaos. S015.

**S015** 53:10
So we've seen loads of problems with the portal trying to report adverse event report SUSARs like via the MHRA portal. And I think in some of our other working groups as well, I think other people have also had issues with trying to report stuff, so the MHRA need to sort stuff out at their end, I think.

**S013** 53:33
Yes, agree with that definitely ICSR portal is not user friendly at all.

**S016** 53:40
It's terrible. The setup for people who use the CIOMs for they can just upload to it, but so for us it's useless pretty much.

**INTERVIEWER** 53:58
Thank you. There's some strong feelings about ICSR. I’ve got S015 and then S018. Oh, were you a legacy hand S015? Sorry, S018.

**S018** 54:13
Yeah, I realised that I actually wanted to ask about the, perhaps the fact that I've only had one SUSAR to report, despite having multiple CTIMPs is, I guess this is sort of a question for the other people on the team that actually have much more interaction on this on the site - oh, that's good to know, S019, I feel a bit better now,
on the site side of things, because I've never worked at the site level and so I don't really have much interaction with the PIs and things, but from the normal trial management stuff.
Is I think the, I don't know if there's any solution to this, but the causality assessment I think is an imperfect system and I wondered what people's thoughts were on whether PIs or the person that sites responsible for causality assessment, whether there's a tendency to down grade them or not, so this is an open question. I'm trying to lead anyone in any particular direction, but you know there's a, you can get lots of SAEs reported and it really is just up to an individual to kind of decide if they think it's related or not. And then we don't really question that. You might question it if it seems like kind of barn door, obviously related that you know there would be a discussion then with the person who's made that assessment. But I kind of feel like most PIs in my experience, just sort of tick the unrelated or the probably unrelated box, and that's sort of the end of it and there's no real evidence that we collect as to what they've considered in making that decision.

Of course, in the guidance and in the training, there's things like, you know, think of the timing of the event, think of whether there are any other possible causes, but we don't really actually get them to write down anything about that decision making. Or maybe they do in the medical notes, but they don't on the CRFs. And I sort of wonder whether, overall, we may be massively under reporting SUSARs just because potentially there's a tendency to just kind of tick that they're unrelated and then we all think, oh, thank God, now we don't have to expedite that report that so and so made and so we will move on with our lives.
But yeah, that's all it really.

**INTERVIEWER** 56:42
Thanks, S018. S013.

**S013** 56:46
I'm going to say that the opposite happens for us, S018, to be honest. I sometimes find that we get people saying that they think they just think, oh, it's possibly related and we get more, we probably over report, to be perfectly honest. And I'm sure S016's going to agree with me when I say that we overreport because he tells me that all the time. But I think especially like we've got a study, it's good for [drug names], and sometimes some days it's an event, will happen and they'll take it's possibly related to them all and then we'll send this to the CI for review and she'll always say there's no way this got anything to do with the [drug] because that can't cause anything like that, but the sites will just tick every drug because they just don't know. So, we get that kind of opposite thing happening. And then we sometimes end up with SUSARs for things that potentially are probably not SUSARs because they probably should have looked at a bit closer and looked at the reference safety information and thought, OK, well, it doesn't actually cause that, so, that can't be the reason for it.
But yeah, I don't think there's really any easy way to answer that question though, because if we get something that's related, we don't, we're not clinicians. We don't always question it. Our CI will question it.
But I was what was I going to say? I was going to say something. I say ICSR’s rubbish and there's something else that's going to say it's going right at my head, so I'll just shut up while I see if I can remember. I was going to say as well.

**INTERVIEWER** 58:12
No, we can come back to you in a minute, S013, when it suddenly pops back into your head. And I actually just had a kind of follow up question for you about the potential over reporting and how often do you end up like rescinding your SUSARs that you report and then having to you know like you get an update and you're like actually it's not related and so that's no longer a SUSAR. Does that happen with any frequency?

**S013** 58:35
Yeah, I mean, that happens. Yeah, that happens because you can have maybe have only seven days to report something that's life threatening or fatal or 15 days. And if you're struggling to get a bit of information, we always err on the side of caution and report.
And then something may happen. Something may change, so the patient will have a scan, it shows something else. Therefore, they realise, oh, it's definitely not related to the drug. So, we do have to downgrade. So that does happen, yeah.

**INTERVIEWER** 59:01
Thanks, S013. S019.

**S019** 59:04
Yeah, it's really interesting, S018. I think it's really subjective depending on the investigators, in my experience. A trial that previously I worked on was a stroke study and you know over 200 SAEs or something like that. And I think in my experience for that trial specifically, it depended on the openness of communication between the local investigator and the CI. So sometimes if we had and SAE come in that they assess as being related, sometimes you can kind, the CI would have a conversation with them to find out a bit more and then they would downgrade off that, which I think is a bit of a grey area because some people don't think you should do that and others think you should because some of it's retraining, you know, if you get an SAE and they say it's related to the drug, but the drug stopped two weeks ago, that doesn't make any sense. So, you can go back and say no. And then in the instance where we had the, the one SUSAR, honestly, it was just pushed from a clinician ego perspective.
They were not receptive to being asked why they thought it was related, so they really hunkered down on it being a SUSAR. And it was, it was very strange. Even if we, you know, from a placebo assessment perspective, they said it would be and the placebo was saline. So, it's interesting and I think it there is an element of subjectivity and politics sometimes, I think. If I'm being really honest.

**INTERVIEWER** 1:00:39
Thanks S019.

**S018** 1:00:40
I would agree with that politics bit. The one SUSAR I did have it was a battle between the local PIs in the international, our PI here in the UK and the sponsor the overall CI in Australia. And they're pretty much having an argument about it. And then the sponsor won and that's why it was a SUSAR and I had to get reported. And they'd argued up to the point of like the 15th day. And then all of a sudden had to be reported.

**INTERVIEWER** 1:01:06
S014. And then I've got S016 after S016.

**S014** 1:01:12
So this may be a little off topic, but it's a kind of triggered me when you're talking about marking it as unrelated or probably unrelated.
So we've got different points and scales that we use for our AE logs, where we've got a three and a five point that we tend to favour. And I just wonder whether there's a bit of a sort of obscurity as to what you know if this, if you're using different types of logs and how they become this SAE, how they become the SUSAR, that translation of that particular range. Was it appropriate for that trial and what decides what's going to be appropriate?

Because I've asked a number of times where we've got it written down as a rationale why we use this scale for this protocol and this scale for this one and it's not down to necessarily phase or anything, it's very difficult to try and get why we do one thing over another.
And I worry that that sort of transpires to a different reporting level if we're not using the same one. And quite often, we'll get there, the sites are made-up their own scale entirely if they haven't got an AE log, so they might be using a four-point scale and they've got to go back and have everything reassessed again, which obviously gets them grumpy. So, it's kind of what does everybody do in the lower stages before we even get here to decide.

**INTERVIEWER** 1:02:44

S016. You're on mute, S016.

**S016** 1:02:52
I’m going to add to what S013 was saying, really. I see everything based on what type of trial I've done. So, when I worked on a cancer trials unit for the phase three trials, a lot of the PIs were rather blasé about whether things were related or not.
And you know, and then you've got to work in other trials where they err on the side of caution, most of the time. But I think I think the only thing we do as a sponsor is we just want it to be the local investigator who carries out the assessment. I don't think we ever really questioned their assessment. Certainly not often.
Unless it's clearly daft, you know, like the SAE’s 60 days post drug, for example. Then we would query it and say what I don't think so, mate, do you know what I mean? I'm kind of like I'm, I'm that kind of thing we would blatantly do. And then other times, you know, most of our SUSARs I've had in the emergency care trials because I've upgraded them rather than the CI for example.
So I've had a look at them and gone, no, this is going down as unexpected and a SUSAR, thank you very much. Because you sometimes do get a bit of a reluctance by CIs particularly to upgrade events because they don't want SUSARs in their trial.
You know, and then on the other hand, you get people who are overzealous and SUSAR everything. So, it just entirely depends. I mean, the way I look at it, is if the local PI has assigned causality and signed off on it, then I'm happy with that. I don't need to question it any further unless it's clearly daft.
You know, that's what the MHRA prefer, is that the Local investigator looks at causality, and we're tending to just leave it at that now. We don't question them unless I mean we, unless it's daft, obviously.

**S014** 1:04:50
And if it's, if it's out of window, that's all that's. That's an obvious one, isn't it? But it's other thing.

**S016** 1:04:53
Yeah. Or it's something like. It's a [disease area] trial, and this is one of our most common ones, it’s a heart failure trial, we get an SAE of [event] and they say it's due to the drug that's supposed to be curing [disease]. And then and then we tend to query it going, come on, mate. Do you know what I mean? I doubt it.
And there's that and also like I we also, every trial unit I've worked at, we've always just limited their options. So they can say it's due to drug or it's not.
They don't get any other option other than that you have a say it's due to drug or it's not. And we limit it to that rather than having these kind of granular assessments, because the problem with a granular assessment is if somebody says it's possibly related then how then then somebody else, how can you, then you can't. It's not consistent. How do you ensure consistency in that? And when you report, it doesn't matter what the granulation was, it's a yes or no.

**S014** 1:05:53
Do you see what, I'm pleased you're saying, yes. So frankly because it's always been a bit of an odd concept to me to have such a range on why you'd have different ones that I didn't.

**S016** 1:06:04
Yeah. I mean, there's papers on this where you basically like, there's whole papers on this about the assessment of causality and the general leaning is towards a binary choice of yes or no.
I mean, again, device trials are a pain in the bum because they have 5 and they want you to use them. But for CTIMPs, it's generally, I mean a binary choice. It's a binary choice anyway. At the end of the day, it's either related or it isn't. You know, so why? Why try and grade it?

**S014** 1:06:36
I do feel like we're trying to catch out the sites on the monitors for spotting the difference on something that we don't really want to know the difference in.

**S016** 1:06:44
Yeah, exactly.

**S014** 1:06:45
And data guys that are doing all this verification. If it's a yes, no.
Related not related. You know it's just more room for errors, isn't there? If the more choice we give, if we're not going to report on it.

**S016** 1:06:59
About consistency, it's like, how can you be sure that that one person's probably is another person's probably? You can't really.
And that's what I find difficult. And it's always about consistency of everything, you know. It's why we took expectedness off sites, for example. They weren't being consistent. I don't know if we looked at what our CIs were saying. They weren't being consistent, so we shifted it away from them to make sure that we could have a kind of it's on the list, therefore it’s fine, rather than them taking into account well, I don't think it's expected. Well, I don't care what you think. It's written right here.
So I'll be quiet.

**INTERVIEWER** 1:07:45
Thanks S016. S018, I'm going to let you say your piece, and then I do because I'm just looking at the time. So, I am going to move us on to the next section to sort of start winding down a bit. So, S018.

**S018** 1:08:01
Yeah, I was going to say that we've just finished doing our sort of all singing all dancing template electronic SAE form as part of our Little Pharmacovigilance working group and our CTU and we umm-d and aah-d went round the houses over every component of the form but also particularly over the whether to do the five point relatedness assessment or just a binary related or unrelated and we ended up sticking with the traditional five point method. And I think I think our only justification for that was that some, I’m sorry I can’t remember where I heard this from or where someone else on the team heard it from, I think we've had some investigators who kind of weren't so sure about making such a definitive putting such a definitive answer. And so therefore they liked the option of saying, like, possibly or probably so they're not being so like, definitive but ,yeah.
Don’t really have a very good basis for the decision. In the end, it was just kind of I guess we must have just taken it to some kind of vote or something. But we've, yeah, we've stuck with the five-point system.
But I just wanted to briefly touch on the whole, how does the how do these investigators make this relatedness assessment? Because I think it is an imperfect system. That's so it's going to be so variable that it could, unless there's already been some, be an interesting piece of research on its own, where after people have reported an event, if you could sort of speak to the PI and do a brief interview with them about their decision making process, not in a bid to change their mind or negotiate their decision, but just to understand more about the decision making processes, it could be an interesting thing for someone to do, not me. I don't to do it, but put the idea out there.

**INTERVIEWER** 1:09:49
Thanks, S018. I'm not stealing your ideas. I'm just jotting them down for prosperity. Of course.
So honestly, I wish we could talk about this stuff for hours, but I'm aware that people are very busy and so I was trying to think, what can I, you know, cut from this conversation? I don't want to cut any of it, but I think what I would like to just move on to as we start moving towards the end is about. So, I know this has come up a few times, kind of peppered throughout the conversation. So, it's probably quite a nice place to tie this all together is and I just put here. This is just my opinion that to implement safety reporting processes effectively or staff need to be trained adequately. I hope you think that has a fair statement, but I suppose what I want to get at is so you've some of you have talked a bit about kind of you know how your training processes currently work.
You know how effective are they and what else could we be doing and where are you know, strengths, weaknesses, barriers, facilitators, those kinds of things. S015.

**S015** 1:11:00
Well, one thing we could do, I guess probably we do is limit who can do the reporting. So, on the delegation log, there's some of the reporting's only delegated to clinicians, for example, that have got experience and are working on the trial from a day-to-day perspective, not just the clinicians that step in occasionally and do something. It has to be the more senior ones that are making the decisions, and they have to be the ones seeing the patients regularly at the trial visit and maybe even responsible for their day-to-day care.

**INTERVIEWER** 1:11:40
Thanks, S015. S019.

**S019** 1:11:45
Two points, I guess. So, in terms of AE training something I found recently a trial that I work on is a CTIMP but it has some research naive staff because it's being split across university and NHS sites and the training of research naive staff and AE reporting has been a challenge all in itself. So, kind of adapting how you train.
Sites based on the audience of which you're delivering it to has definitely been something that we've learnt recently. You know because, we take it we take for granted, saying assess AEs at this time point. Some people don't know what that means. They don't know what questions to ask if they're not clinical also. And then I think from a CTU perspective, something again that's come up for our unit has been MedDRA training. So, I came from a CTU, that was very CTIMP heavy and I've been at [location of CTU] for about a year now. Where we're picking up a lot more CTIMPs etcetera.
and MedDRA training is certainly something that's come up for us and making sure that we're doing that and implementing it very early on, so you don't end up with this backlog that comes later on. And I know there's different ways that you can implement MedDRA coding. Some trials might ask sites to do it, some sit with the CTU, some sit with the sponsor. And again, I guess that's a kind of the lack of consistency across trials, units and places. But yeah, so from a CTU perspective, I think that's a training need that's flagged up for us.

**INTERVIEWER** 1:13:20
Thanks, S019. S013.

**S013** 1:13:24
Oh, yeah, yeah, I think I kind of mentioned training a little bit earlier and I think it has to start right back at CI training. And I was actually part of a CI training working group and it was about new young CIs and what they need to know about studies is they're starting to take them on. And it was things like importance of PV processes and safety reporting processes. But I also think it's actually a really hard job to train somebody to do, to report SAEs and to process and clean SAEs from both sides of the like from the CTU sites and from the site aspect. And I've worked on both sides and you can sit and read SOPs galore and read protocols and read completion guidelines, and you don't actually figure out what you're doing until it comes to actually reporting an SAE or cleaning an SAE. I've tried to train staff both sides and it is really difficult. I think it's good to have SOPs, working instructions, the protocol needs to be written clearly so that the site know what they're doing and we know what we're looking for. Having the correct IBs understanding the difference between an IB and an SmPC.

So I think it is a really big thing, but I think it's really difficult to do. And we do obviously have our site initiation visits to train our sites, but you can sit and talk through slides, show them an electronic SAE form, but nine times out of 10 when it comes to reporting an SAE, they've forgotten and they'll phone you and go, I don't know how to report this SAE. And it’s like, well, I did show you and there are completion guidelines, but let me talk you through it. So, it is a really big thing training and I don't know how. I don't know what the answer is to make it more effective, I suppose, but it's a constant issue.

**INTERVIEWER** 1:15:05
Thanks everyone. And so, there's stuff going on in the chat as well. So, I don't know if people have got half an eye on that as well, but yeah, difference in experiences of different levels of experience and research naivety, visual aids and flow diagrams have been useful. And S019 suggests Drawio is a good resource for that. So, thank you to those of you in the chat.
So, I'm really aware that we're about 3 minutes away from being at our 90 minutes. So, I just wanted to, I suppose my finishing point is, is there anything else from any of you? And I suppose one, I mean, maybe that's probably not the time to be raising this question. And I know that it came up right at the beginning, but just how closely you think that the work that we're doing in safety is actually like filtering down to keeping patients safe? And what that connection is between what we do and patient safety and how you kind of feel about that.

**S019** 1:16:12
Big question.

**INTERVIEWER** 1:16:14
Yeah, I've realised that that's probably not the question to ask right at this point in the proceedings, but I'm going to ask anyway, S016.

**S016** 1:16:22
I mean, I've only had it once where I've had a direct thing on office in the one of the trials I worked on when I was at [location of CTU], we actually identified a proper signal in a clinical trial, whereby we were getting lots and lots and lots and lots and lots and lots. I think it was in the end it was like 20,000 SAEs or something.
And when we looked them by arm, like it was very clear that there was, a constantly being assessed as due to drug as not due to drug, so unrelated but present.
In all patients in one arm, but not at a massive level, but inflated levels to all the other arms.
And so we ended up having to look into this.
And turned out that people with a certain genetic mutation ended up with a side effect that was being experienced by lots and lots of patients, but only in one of the arms. And so it fed directly into guidelines for that drug. That's the only thing that's the only time I've ever seen any benefit to it.
Rest time, I just feel like you're going around the treadmill like a hamster, but you know. But it's important to notice that it does actually sometimes feed in and that actually made me feel like I'd done something worthwhile. Possibly, rather than just gone number goes up and thing goes to MHRA. So yeah, that's for the only time I'm afraid.

**INTERVIEWER** 1:17:47
Thank you, S016, S019.

**S019** 1:17:50
I think S016’s explained it really well. I think for a period where we're over reporting due to an abundance of caution, despite what the MHRA and the regulators are saying and over reporting is masking any of the important information that we actually want to know. I think that probably what, I think summarises where we're at.

**INTERVIEWER** 1:18:15
Thanks S019. That was a really nice little kind of footnote to today's discussion. So, before I wrap up, does anyone have anything like burning that they want to say that they haven't had the opportunity to say so far?
OK. And I imagine everybody's probably ready for a cup of tea, so I'm going to stop the recording.


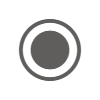
 **interviewer** stopped transcription

**Transcript Focus Group 4: 04-Mar-25**

4 March 2025, 02:12pm


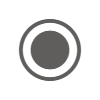
 **Interviewer** started transcription

**Interviewer** 0:03
Hopefully you should have had a little notification pop up to say that we've started recording. So going to start with some opening questions, some nice easy questions just to get us settled in and make sure that we're all starting from the same point and that we're all thinking about this in in the same way. So my first question is what do you understand by safety reporting in the context of a clinical trial? I'll throw that open to the floor.

**S020** 0:34
I understand it to mean according to your protocol. Reporting the safety of the participants on your trial according to the protocol and according to what you're studying in line with your sponsor requirements.

**Interviewer** 0:51
Thank you, S020. Very succinct, clear answer. Anyone got anything to add?
S021?

**S021** 0:58
I suppose, yeah. I'd only had and also regulatory requirements. Otherwise, I think yeah, that really that covered it really well in my head.

**Interviewer** 1:10
Thanks, S021, anyone else? We're all happy that we're thinking about this in the same way, OK. And then just to kind of add to that. What would you say are some of the main tasks and processes that constitute safety reporting? And pharmacovigilance, so we're thinking about specifically from the point of view of CTIMPS today.

**S022** 1:38
So I tend to think in terms of the sites have got the task of monitoring for doing ongoing monitoring for adverse events, serious adverse events, anything that basically falls under the banner of adverse event.
And determining like you said, like S020 said, according to the protocol, whether it needs reporting on to the sponsor. And then from the sponsor or the sponsor delegates office, sort of determining whether it's looking like it falls within potentially needing expedited reporting timelines.
And whether it might constitute any other urgent safety measures and then obviously then take it through processes for having it reviewed by chief investigators in according to what processes you've got defined in your risk assessment. So yeah, for review by Chief investigator of the members of the trial management group and then so I was talking about the expedited reporting, but then obviously there's the annual report that I whinged about earlier. So yeah, there's some of the main tasks.

And in terms of, I suppose there's a lot of other dialogue that goes on once something's initially been reported by a site between our units, our data management team rather than so we have a staff of divided into data management and trial management and the data management team tend to be leading on the pharmacovigilance and it's, you know, a lot of dialogue then going on with sites, just ironing out queries, inconsistencies. You know, any missing information and taking it through to its conclusion really. So that's, I think that's a really, really big part of the time-consuming element of it.

**Interviewer** 3:23
Thanks, S022. And yeah, I definitely resonate with the dealing with queries and all of the inconsistencies, all of those things. And anyone want to add anything?

**S020** 3:32
I completely agree with everything S022 said. What I would see the primary main task before everything that S022 quite rightly documented there is the main task for safety reporting is identifying.
Something that requires safety reporting correctly according to the protocol. So, the categorization of an event into a safety report and determining whether or not that requires a safety report and what category that goes into is the main primary task is categorising your information correctly.
And then you can save yourself a lot of that to-ing and fro-ing with the sites. If your protocol is clear and all of the associated processes with that are clear.

**S022** 4:21
And I would, I would 100% agree with that because I think when we were first saying what our experiences were at the start of saying, just deciding what you're going to put in your protocol and you know what level of scrutiny you're going to go into, I think that's I found that it's so subjective. I felt it was so subjective when we were doing it and I felt very vulnerable doing it, making those decisions because our chief investigator wasn't very.

**S020** 4:34
Yeah.

**S022** 4:44
Experienced either. So yeah, I would 100% go. That's your first starting gambit. Just what where you know, what do you want to even collect?

**S023** 4:59
So I would agree with all that absolutely first step, get your protocol right. Make sure you're very clear on the protocol, what you're collecting, what is what isn't, and if it is timelines, when does it need to reported by and who does it need to be reported to?

**S021** 5:17
Yeah, absolutely. Especially from a QA perspective, that's one of our key things is getting that protocol written as well as you can in the first place sometimes, I suppose it's going to be matters, you won't know at the start or you can't be sure of. And sometimes therefore you need a review phases you know establish where in your monitoring plan where you think you're going to review your current safety reporting profile.
But adding on to that I suppose even is, I mean I guess this is actually defined by other matters, but what drug are you are you testing? And then defining your RSI by that as that's key to part of your safety reporting. So yeah you can go as far back as that even, isn't it really when you're talking about the tasks and processes involved. As it's it sits at the heart of any clinical trial with a drug is safety.
But everything else as well, yeah. Absolutely. And I think that's a curious part. And I guess why we're here, where do we think the time saving can be? You know, where what tasks are the most resource heavy or unnecessarily especially resource heavy.
Where we do it is Trial teams. We have a trial team TM, probably a data clerk, maybe a trial manager trial coordinator. They do a lot of that work. They do a lot of the work and as QA we have oversight, and we advise and inform. But we also, you know, establish the SOPs for how we're going to do this and review, you know, review their SAE reporting forms.
Review the protocols and the Safety reporting section to help inform that so kind of getting away from the main question. I suppose a little bit there, but these are all the parts aren't they that come in. These are the resource usage that go into establishing what your safety reporting is.

**S022** 7:18
Yeah, I'm now would I would argue, not really get moving away a tangent. You know, you're just taking it up to the, the whole thing, isn't it? You know, it's not just on the individual trial. It's deciding what your processes are for training people in, you know, the SOPs and well designing your SOP and then making sure that people are trained to understand what to think about when they are designing the protocol. So yeah, it's a very big flow chart, isn't it, of tasks which where they all come relevant.

**Interviewer** 7:59
I just want to pick up on a point that was made about how I suppose how do you decide what information you're going to collect? What is that that process like, for you?

**S022** 8:23
I mean, I think you, you obviously your RSI is going to have a lot of description in of you know the previous anticipated events and what have you.
But the particular trial that where this issue with the DSUR arose is looking at some drugs that have been in use since the 1950s generics and they have so many side effects at different doses.
So, sometimes these one of them is sometimes use oncology and much higher doses. But in our study, it's at much lower doses only weekly and you don't the safety profiles differ, but the RSI well there's the Summary of Product Characteristics, doesn't really differentiate between high dose and dose and low dose and whether safety events will occur. So, but I mean I think that's certainly one of the starting points for deciding what you want to collect.
But, I suppose I'm sort of like dodging the question because I almost say the same thing as you just asked about. How do you decide? When this it could these drugs being around for so long. So, everything's been reported everything that possibly could ever be reported to anyone. So, you could have two hundred 250 things classing as AEs if you want to go down to that level. So, I guess you have to be driven by what might all your safety data collection add to the known safety profile of the drugs. And you know there's an argument, I think in our in our study to say, it probably won't add very much at all. Yeah.

**S021** 10:08
Yeah, I'll be happy to jump on the back of that and where we are, we mostly handle phase three phase four, which I think that in itself probably plays a part in deciding what information do you need, what should your profile be.
So most of the drugs we handle similarly, they're well used, they're generic. But they may come in lots of different formats or strengths, and we might want to use them in a slightly different strength. Which can cause I think that does cause some resource issues.
Probably better if I had a trial manager here with me who could answer that a bit better because they would have done that hands on work of IMP selection and RSI, but I'm aware that that in itself can have its problems. While I don't know, but phase one phase two, perhaps it's maybe more straightforward, or maybe it's worse, I don't know. Yeah, sorry and probably not adding loads there, but I agree with S022.

**S020** 11:09
I think we end up over reporting a huge amount of things and I think it's sometimes that's the veil of the spot, the particular sponsor you have governing the study on top of what you're actually doing, because we all know, you know, dragging it back to the original definition, anything that we're doing to a patient that is different to standard care because they've signed a consent form in our study that causes an event to them because of taking part in our study or taking our drug, then that is the definition of an adverse event, an adverse reaction, a serious adverse event, you know, depending on what tick box you know, it drops down into.
I think the complication comes when, and I've worked in and out of different sponsors for the last few years, and I have seen different approaches to it, very risk averse and very, you know, very, very relaxed, not, relaxed is the wrong word.
Very compliant and secure sponsors and then very, very insecure and risk averse sponsors. And that has added an enormous veil onto the top of it where we have found sponsor almost forcing us in protocol to classify things that are happening to patients in the area that I worked with major trauma where literally everything by definition that comes through the door is a serious adverse event. It's something that's happening to them that puts them in hospital, changes their life, maims them, you know, it's and it becomes very, very blurred and you end up continually reporting and reporting and reporting and reporting and really, you know, if you keep dragging the definition back to what have we actually introduced to them, that's different as a result of being on this trial that's what we should be looking at and, you know, reporting that in line, as S022 said, with the safety profile of the drug or the device, depending on what you're doing is a really clear way of saying it. But each protocol under different sponsors can have different interpretations and can send you into a reporting frenzy that can confuse everybody. And can, you know, takes you further and further away from your original sort of biblical meaning of your protocol.
And that can be really difficult, and everybody wants to overreport, it's better to over report than under report. We all know that, you know, safety first and but sometimes I think, the over caution the risk aversion and the over reporting can jeopardise the delivery of the research, can jeopardise pushing forward good science in order to answer a question. We can be tied with so much red tape that then we've forgotten what we're doing.

**S021** 13:52
Yeah.

**S023** 13:53
I don't think I've got anything further to add. I would agree with everything that everybody said, definitely.

**S021** 14:00
Yeah, I know. I was only going to add on there as S020, you were saying it risk proportionate, isn't it? I think we've tried to go down that path and I think impacted by, we run a lot of non-CTIMPS and that's its own kettle of fish of safety reporting. In a way it's kind of easier but in a way it's kind of harder because you don't have established safety profiles. So, it's all very much down to the definitions in the protocol. But, Yeah, risk proportionate we have established in our SOPs.
General examples of when in a CTIMP, and this probably like I said again reflects working in phase three, phase four trials as examples or rough examples of when you could exempt certain AEs or SAEs from reporting. I mean we state it should be defined clearly. It should be justified, but we've got that in there, I think to help support not over reporting. I mean, yeah, generally it's, be risk averse you know, be worst case scenario.

But, also, because we know the nature of the drugs we're testing aren't well, it's usual care. It's often usual care, the usual care. You know, we're just trying to see which drug is better, you know, which one has the least amount of worst outcomes or provides the best overall outcome. So yeah, yeah, I mean that's a key area. But whether or not that's defined by the level of trial you're running as well you know.
If it's drugs that are well established and you're not really testing them in a particularly novel way, then I think you can be very risk proportionate. But otherwise, I suppose maybe you have to be very risk averse because you we're, I think we're leading into the liability then isn't there and that's probably where you say in your in the PIS for this you know that's where a lot of sponsors of are not are very worried about very cautious. Understandably.

**Interviewer** 16:06
S021, just as you were talking, you might have seen the slides moving and it's because you pre-empted me and you started talking about risk proportionate approaches and it was something that I was actually going to ask about, was about, about the information that we're collecting and you know how are you using risk proportionate approaches in your trials and kind of how is that being implemented within your units and within your trials. So, if anyone has more that they want to say on that. Please do.

**S022** 16:45
Hi, yes, I'm just looking at our guideline template for the protocol template, in the pharmacovigilance section. So, it does talk about a CTIMP with a well-known safety profile using licenced drugs in licenced indication.
In such trials, it may be considered appropriate that certain AEs and ARs are not required to be reported, but should be, so don't need to be reported, but should be recorded. Potentially they don't need to be reported if they will not improve the knowledge regarding the safety profile of the drug and are not required for the trial analysis. So even though we we'd still, but we will still ask the sites to be recording every adverse event that happens in and seeing it through. So, you know, even just a minor cold or something like that. You know technically they should be recording that.
We don't want it reporting to us, so obviously that helps our burden.
But it doesn't necessarily help sites burden. I'm suppose they don't have to report it to us, so it takes away the burden of them having to do that. But in terms of, you know, they're still having to record it all.
So yeah, we have got allowances for disproportionate, but then you'd get very little guidance and I don't know that the SOPs really could because there's you're going to be looking at such a wide variety of drugs, but you know of what sorts of things you can exclude. And it says, yeah, you can, but doesn't then give you any further idea of how much you can exclude. Which is why I think we're on, I was saying, well, maybe the thing to be guided from is whether it'll add anything more to the literature and safety, known safety profile of this drug. There's so much literature out there. What more could you get? And other than the collecting SUSARs.

**S020**:42
I definitely think for lower risk trials and well-known drug profiles, that's completely valid. I think it changes a little bit with higher risk stuff where you're you know maybe using it off label or your dose loading or you're doing something like that then you know I think there is higher anxiety, and I understand that.

**S022** 19:04
Even using off label is a little ambiguous, potentially, because things are when something's a generic, in our particular trial this it's not on the in the label not being used in its licence indication. But in standard care it is commonly used for this indication, but you know if you're driven purely by whether it's part of its licence indication, it's not.
So technically, it just becomes a little bit again, and again it's just another grey area really, and I don't know that there's very much in the regulations that is there. Is there much in the risk adapted guidance. You know the grey guide? I don't think there's much necessarily about that slims down says you can not report this. Not report that, but I could be wrong.

**S021** 19:59
Yeah, I can't say off the top of my head, it’s been a while since I looked at it. A point that grows with me with my experience in clinical trials more and more is justification. If you can justify it and then assuming it's in, you know you're putting in your protocol, you're getting that approved at a REC, which you will be, it feels like, it's not as definitive as I suppose it would be nice as we'd like it to be.
But it just seems more and more that it's whether or not you can make your case as to why it shouldn't be and if you're not making a good enough case, or if you're not justifying why you shouldn't report certain things, then that should hopefully get pulled up and brought, and then you need to reconsider.
Yeah, it's a bit. It is bitty there, it's not clean and cut, at least not with lower risk trials. I suppose in some ways your every time we run one, we're perhaps looking at what the boundaries are in terms of that. Whether or not that’s a good or a bad thing, I'm not sure, you know, ethically and morally speaking, I guess.
I mean, I guess in addition to note, I was thinking of is I guess something to be wary of is cumulative AES, isn't it as well. If you don't collect AEs because you've said we don't need to for this reason, there could still be value and understanding though what, how many people are getting colds? You know, if this was a drug that was not known to commonly give people will get cold's taking, but then a large percentage of your population on your trial is. How would we know that if we've decided not to collect or we've justified, we're not going to collect that that information? Yeah, I don't know where the line stands there really, unless it's it probably does come down to context of the drug you're testing, doesn't it, of the drug you're looking into. You know, if it's really that well established the profile. But yeah, what's the chance of you learning something new?

**S022** 22:17
I suppose that's where you slip into being able to collect some safety events as endpoints rather than actually pharmacovig-, for the purpose of pharmacovigilance and the onward reporting. And you can still get them, still collect the information as an end point as an end point.

**S020** 22:36
Anything known in a drug? Oh, sorry, S023. Oh, no. I was just going to say anything. All I was going to say was I think anything known as a safety as a, as a contraindication that's written, you know, in a drug pack. It should be put as an outcome. It's not about avoiding collecting data. It's about walking the fine line between fully reporting outcomes having reportable trial that is responsible that provides you DMC with enough data that it can make correct decisions, you know that are statistically weighted, about the trial, about collective patient safety that definitely, definitely has to happen. It's not about getting out of work or not reporting anything, but it's about walking that fine line between absolutely overburdening not just the trial office but the sites themselves. We all know post COVID how hard it is to engage sites, have little capacity, people have.
And it's about not making research so burdensome in terms of reportage that we put people off and we ultimately you know we don't get sites or we don't get the correct report they don't engage with it properly. So, it's walking that line you know I think it's quite hard. Sorry, S023.

**S023** 23:54
No, you were saying exactly what I was going to say. It's not about not collecting. And sometimes we collect it. So, we've got that information for safety committee type things but we're not on board reporting to sponsor MHRA REC that type of thing. But we're still collecting the information.

**Interviewer** 24:17
I think there's been some really interesting points raised in this discussion and I'm just trying to think like what's the best way to move this forward, because I think we've kind of been talking a bit about some of the challenges of trying to implement these risk adaptive approaches and touch a bit on the guidance from the MHRA.
But I just wondered, in terms of what the regulators do have in place and what the MHRA does kind of say that you need to do. How clear is it, I suppose you've kind of touched on this a bit already, but kind of how clear is it to you how the information being collected is then being translated into the safety profile of the drug and what that means for patients? Because we've talked a bit about the impact on our workloads and on sites, particularly given the current climate, and I just wonder about how do you feel about how that trickles down into patient safety and patient care?

**S020** 25:47
Sorry, I'm not quite sure I understand the question. So, you're asking how we feel about the data that we collect getting into patient care. I hate classifying it into MedDRA terms, if that's what you mean. That's the first. I think that's really hard and a complete dark art. And I just, I'm terrible at it and I don't want to do it. But do you mean?

**Interviewer** 25:59
Yeah.

**S020** 26:09
Sorry, could you rephrase the question? I'm just not quite.

**Interviewer** 26:11
So yeah, I'll try and kind of re rephrase it in a way. So, in my head it made sense. So when-

**S020** 26:14
Sorry. It probably did for everyone else, it's probably just me.

**Interviewer** 26:20
Because everybody's looking a bit blank, which suggests that I have not worded myself very well. I suppose I'm trying to get an understanding of how what we're doing here. Is there an understanding about how this information that we're collecting and all these things that we may or may not be collecting, how does that relate to keeping the patients safe? Like how does that line sort of follow along that, you know, we're collecting this information or we're not.
And I suppose it's kind of trying to understand how you see that information translating into keeping patients safe.

**S022** 27:04
So I think there's two, well, I think there's loads of elements to the answer to that question.

**Interviewer** 27:11
So it's a big question, I think.

**S022** 27:13
Yeah. No, no, no. And as it should be because, yeah, no, it definitely should be. So, I suppose with there’s two sets of patients that you could be considering and that's the patients in your trial or the patients who subsequently could be treated with this intervention after your trial. You know, if they if the study, come, you know comes positive outcome, and the patients, it could potentially become standard care.
And I think you might have sort of revealed if everyone, I mean everyone else might be going. Yeah, I understand exactly how it that information is used like downstream and what have you.

But, there's a bigger question to an extent that how much does all our clinical trials work always get translated into changing clinical practise anyway. And I don't really personally know that very much about how much data there is available to show how much you know it all our trials will change clinical practise. I think there are you know there's entire Departments over at [University], I think looking at this kind of thing.
And then you know your safety data collections are sort of a small part of that, isn't it? So, if the whole, if it's a little bit ambiguous how well our trials do change clinical practise anyway, some of them do definitely. We know. Definitely we know, certainly some of them do, but if things don't philtre through anyway, then again it's a whole lot of data collection that maybe has very little impact, in the long term in terms of the trial patients actually within your trial at the time, then I guess that's the DMEC isn't it, whether they, if you're collecting things as outcome measures and they're able to see that there's a much bigger burden of people getting colds in Arm A versus Arm B.
There's a potential there for that to have an impact that they might pull, you know, advise that you either modify your dose or I think for colds using that you know there's the basic thing then you know they're not going to say stop it, but it might impact on some elements of the trial delivery.

**S021** 29:43
I'll jump in. I mean, if I've understood how, I think what you were saying is,
I suppose frankly I my primary concern is, I think, about how it affects patients on the trial.
And the actual there's there are different aspects, as S022 was saying. Then there's the impact of your outcome of your trial and how that impacts. But then the accumulated safety data, how that directly effects the profile of the drug, which I think is kind of what you were, you were sort of asking about, I think it might have my interpretation.
I'm not really sure there's my honest answer there on that bit. I'm not really sure. I guess my focus has always been more on the impact on the trial and the safety of those, of the participants than the long term other than you know the success or what's a success, success is completing the research and having good quality data, you know. But yeah, yeah, things like the DMEC who can review the events and look at whether or not it's, it's safe for the patients involved.

And yeah, I can't say. I personally think I've thought too much about the downstream of where, where does that data particularly affect the, I guess the SmPC of that drug, but that might just be because of my position. You know my experiences and my involvement that I haven't really thought about that as much, that that that's my honest answer on that bit.

**S023** 31:27
Again, I would agree. I'm not sure where how the results of trials get fed back into SmPCs and things like that. One assumes it's kind of big meta-analysis. Nice Commission. These types of work.
But yeah, I've not really thought about, I've always focused on collecting the information for my own trial. How that implements into standard care and safety profiles of drugs and stuff, I'm not entirely sure, but probably should be more aware.

**Interviewer** 32:04
Thank you. So I know that was a tricky question, but it just from what we're talking about, it kind of just made me think about that kind of bigger picture.
I think if no one has anything to add to that point, I'm going to move us along because I'm also conscious of the time.
I think maybe we'll come back to this, but I think we've already talked a bit about regulators and pharma companies and how that can, the impact that that has on how we're setting up our trials and writing our protocols and things like that being, you know, more or less risk averse. And I know that a couple of you have mentioned that SUSAR reporting is not a big part of your job specifically, but it is an important element of safety reporting kind of more generally, and it is a requirement, and we have to do it. And I just wondered what kind of what those processes are like for you and what you think about the process of SUSAR reporting and things like the portals that we use and the processes that we have to follow, if anyone has any opinions on those. Generally, when I ask people about SUSARs, people have a lot of thoughts, and a lot of therapeutic venting happens. So I will, S020.

**S020** 33:31
I haven't actually reported a SUSAR through the newest system, so I'm afraid I don't have any lived experience to share. I'm a bit scared, so I'd quite like somebody to give me, share their experience so I could learn from it.

**S023** 33:51
I don't have a particularly good experience of reporting SUSAR through the new is it the ICSR? Have I got the right acronym there?

**Interviewer** 33:59
Yes, ICSR in the UK. And then there's also EV web is the European one. And then if you're reporting in other countries as well, I think they will have different portals and systems outside of the EU.

**S023** 34:12
So I've only reported in the UK, so I reported using the new ICSR system.
It didn't go very well. Because there was something wrong with the University of [redacted] set up, so I had entered all the data, complete the all the form, checked the sponsor was all happy with it and then when you tried to submit it came up that here was an error, couldn't submit, so we tried to get it sorted. Couldn't get it sorted, so I had to take screenshots of everything. So I didn't lose all the information I'd entered.
Tried phoning a couple of people from the MHRA. Nobody could help me, sent an e-mail. We'd sent e-mail. We followed up emails and eventually at the end of the day, I just had to come off the form and lose everything because I couldn't keep it kind of active on the screen.
So thankfully we were Co-sponsored, so we managed to submit it through the [location] So, we did get it submitted in time, but it took the MHRA four months to sort it. And when we did get it sorted, they said well, do you want to try and submit one? And we were like, well, we can't submit one. We're not trying to submit anything at the minute, and they said you're not got a SUSAR to report. It was like, yeah, but that was four months ago.
If we were only reporting it now, I think you might have something to say about it. So, it wasn't a good experience, but thankfully it's all fixed now. But there was a lot of to-ing and fro-ing and it took a long time to report one SUSAR.

**Interviewer** 35:40
Just hearing that stresses me out.

**S022** 35:44
Yeah, that's horrific. So you'll.

**S023** 35:45
Because I'm sure they would have something to see if it took us four months to report a SUSAR, but.

**S022** 35:51
So, no one else in your unit been having to because I again, I don't have the experience of doing it, but the way you worded it sounds like you have an account for your sponsor for your university. So, it's not like it's a trial specific account.

**S023** 36:07
No. So it's a sponsor account and then the sponsor gives trial managers that are involved in CTIMPs access to the portal, but the sponsor couldn't submit it either from the university account, there was something wrong with the account, how it was set up in the not something that you know we had set it up wrong but something just hadn't quite happened somewhere, and yeah, we couldn't get it fixed, but thankfully we did. We did manage to report through [location] because it was Co-sponsor, so that was fine, but I don't know what we would have done if we couldn't do that. Send them screenshots, I guess.

**S022** 36:43
The yeah and the none of your other colleagues had had to submit a suicide, so it'd never come up before, right?

**S023** 36:43
So, I would that was the first time that the university had to submit a SUSAR on the new system. And unfortunately, it was me.

**S022** 36:56
You must feel. Yeah. Oh, yeah. So, you almost feel like part of the setup of that system setting for the account should be submitting a test case?

**S023** 37:08
Yeah. And it's probably, yeah, I mean we don't think.

**S022** 37:08
You know the MHRA should do that as a standard thing.

**S023** 37:12
Yeah, I don't think anything about it. You know, everything sets up correctly. You can enter the data. It wasn't, and you got the submit button. It went. I can't remember what colour it turns when everything's there. It did all that. It's just when you try to press submit. It said an error and I couldn't save the form. There was an error with saving the form as well so.
But the next one went OK, but there might be something for, yeah, as you say, doing a test one is when you're set up in the system to make sure it all goes through, OK.

**Interviewer** 37:44
S022, this probably isn't making you feel much better, is it?

**S023** 37:48
So S022's at the university, it's all working fine now. So, you're OK.

**Interviewer** 37:57
OK, I think unless anyone's got anything to add specifically about SUSARs, I might move that on because it sounds like.
There's not super loads for people to say about it because it's not something that we're all doing on the regular, so that's fine. It's just like some groups there's been loads of people that are doing it all the time and then others you have less and it just it really varies from group to group, which is also interesting, that kind of variability.
We've talked a bit already about these, kind of, risk adapted approaches in CTIMP trials. I decided before I kind of move us on to the next section. Did anyone have anything they wanted to add about risk proportionate and risk adapted approaches to safety reporting and how that's all working out for you, or not?

**S021** 38:49
I mean, I was obviously already spoke a bit about it and I guess the interview I'd say is off the back of the last one. Yeah, I've got a little to no experience with reporting SUSARs directly, but our TM delegated usually delegated from the CI. Obviously, the CI would be involved. The have the odd ones we've had of which some of them are actually non-CTIMPs, so slightly different.
They've gone smoothly as far as I'm aware, but perhaps part of the reason, not just because we're only doing, you know, as I've said before, phase three and phase four drug trials but is also a risk adapted approach is probably meant we're getting less to report. Maybe, this is anecdotal, but because of our approach towards trying to.
Yeah, take a risk adapted approach to how the safety reporting is done and maybe that that that's because of that we're having less SUSARs. It could just be, though, that the drugs we're using on our trials are just not having many unexpected SAEs.

**Interviewer** 39:59
It's kind of a good thing, I guess.
Just on to the carrying on the point about the risk adaptive approaches, I just wondered if anyone had experience of what it was you know trying to set up your trial and write your protocol with these risk adaptive approaches and doing your risk assessments and things, if you've had any experience on what it was like trying to get that put together and sent to the MHRA, and what that was like trying to do that in practise?

**S021** 40:38
Yeah, I just say sorry. Yeah. Unfortunately, I can't really. That's, it would be the TM and probably actually one of my QA managers who might be more involved in that discussion unfortunately. Otherwise, I'd love to be able to add a bit more onto what exactly the specifics of what we've done or what we do, yes.

**Interviewer** 40:57
No problem.

**S020** 40:59
Before I came back to [redacted], I put in a phase four type A so low risk trial using a drug.
Sounds similar to one of your studies, S022, a drug that's been around for a long, long time. It was being used in a way that it's not usually used, it's been used. No, it's been taken in the same way, but it was used in a patient group it’s not traditionally given to. It's anticoagulant, and what's the name? [drug] given to patients after they've had a stroke rather than before, they've had a stroke.
And that's contraindicated. You shouldn't, but there was some evidence. So, they wanted to try this out. They felt that this would decrease myocardial events. So, this was a very low risk. It's a really, I mean the safety profile of aspirin is so well known. You know, it's absolutely, you know cool. So, the submission of that via like Combined review was very, very easy, but it didn't save us any work because we had to first get through sponsor risk assessment at [redacted] who give the categorisation to that. So, the workload I would say was the same. It was easier to it's easier to get it through MHRA. Yes, since it's classified by sponsor. But you know, to get it classified by sponsor and for them to agree with it was an enormous amount of work, so I think.
It's a risk adapted approach. It's streamlined the safety reporting, but it didn't save any work. For that initial, ahead of what we had to do.

**Interviewer** 42:42
It’s just moved it to be more front loaded.

**S020** 42:44
Yeah, it's just moved it to somewhere else. So yeah. That was my N-of-1 experience with a low risk one. The high-risk ones are, yeah, completely different because there's no way you can streamline those.

**Interviewer** 43:03
Thank you, S020.

**S021** 43:04
I was wondering if I can ask, do you feel that that's, so sounds like then, yeah, it's gone to the sponsor kind of is where the delays and the workload, the resources coming in.
Is that if you can even answer, do you think that's because of familiarity with the sponsors or you know, are you, did you feel your team knew the sponsor well or that they understood what you were managing. We I mean I, I guess I asked this because that where we are our sponsors office they have the overall responsibility but they pass a lot of responsibility over to our unit and our QA team.
Although the final sign offs will always still be with sponsor, we find them, they're a bit distant, you know they're very friendly and all but their knowledge and understanding is probably yeah more risk averse. Because of that I wouldn't, I don't know. I guess I don't know quite what my question is but.

**S020** 44:03
I can try and answer it, I think. I think I was only, this was when I was in [redacted] which is where I've just been for sort of just about a year.

I had personally not worked with that sponsor before, but the sponsor was very well known to the team that I was working with. So, I would say in that sense, our trial team on the whole was like familiar with the sponsor. They are particularly risk-averse and they are incredibly tied up with process and bureaucracy.
Which makes getting almost anything through really, really difficult through Lothian compared to almost anywhere else I've worked, for sure. So, I think it was more a factor of the way that they go about things that made it a really, really arduous task.
You know what we were actually asking. It was a complicated trial that we did with something very simple. And the answer we needed was very simple. And what we were actually doing was very simple, but it was, it was complex around it.
For many different reasons. And yeah, I think that in that particular situation was what caused the, but I think for me, yeah, getting trials through [redacted] as a sponsor is the hardest thing I think I've ever done.

**S021** 45:25
Yeah, I because I thank you, jumping in there asking the questions, sorry, interviewer,

**Interviewer** 45:31
Not at all.

**S021** 45:37
but I was going to, I was going to add on that. Yeah, because I know in some of our scenarios. They basically will turn around and say have QA reviewed it and if we say yeah yes, that's it, that's fine. They'll sign off, yeah.

**S020** 45:43
They've got the assurance that they need, and they should have that assurance because I'm sure that your QA, like the QA at [redacted], is incredibly supportive, incredibly knowledgeable, very, you know, very experienced person. You know it's they should have that trust in it. It's very yes, it's it just varies. I think it just varies between sponsors.

**S021** 45:46
Yeah. Yeah, it's. Yeah, it's curious, isn't it? Yeah.

**Interviewer** 46:13
Thank you everyone, I'm going to move us on again now to something that popped up right near the beginning and I'm sort of glad it did because I thought, OK, we're again that we're thinking along the same lines here is training. So, to implement safety reporting processes effectively all staff need to be trained adequately. I hope you think that that is a fair statement.
But I just wanted to ask a little bit about the training that you undertake within your trials units and your trials. And, yeah. How- What does that look like and how effective do you think it is and what could we be doing better and you know all of those kinds of things?

**S021** 47:03
I don’t mind jumping in first.
I think that's a good point. I've actually, I've even made notes beforehand on this is that. It's probably, I guess that's a good thing because that's a problem. I
receive the SAEs as the QA, oversight, and pretty much across the whole of our department, so that can range from, you know, to a few trials that are currently actively recruiting and reporting to more than 10. So, there is a lot of reliance or requirement really for the trial teams to have that knowledge and understanding of what they're doing with SAE reporting.
And my experience is it's not really there to go back to what are we doing about it and that is a struggle because of resource. We have our SOPs and the SOPs are quite clear what they should do, what they should know. But that's not as simple and we have systems in place for compliance there. You know, that they should be reading them and we may you know we run audits and whatnot. But I guess my experience and knowledge that I've got from it is that unless you're regularly working with SAEs on a trial, you're not familiar with them and therefore you can be very uncomfortable, very unconfident in handling them, and that's a barrier that's a barrier in terms of I think of how you move forward.
And we started working on something my, line manager, she put together a video.
Some training on SAE reporting, but it kinda just goes over the basics. It's really good but it only go it goes over the basics so introducing basically to there's a lot of terminology in in safety reporting that you don't get in the day-to-day stuff.
And I think it's really good, but in my head, I've kind of established this sort of a three well, I don't have three stages is quite right, but you've got that basic level of just introducing people to what reporting is that anyone involved in research could probably do with what clinical trials specifically, but then you've got the next stage, which would be management, which is if there's, you know, if you've got safety reporting going on, if you've got an SAE or a potential SAE, all of the management and oversight of management oversight, checks of that that can be very local based perhaps because that might need input from what your SOPs are.

And then there's perhaps the sort of I guess next level, which is actually establishing, or defining it in your protocol. What you want to be reporting on. What you need to report on justifications for what you're not going to report or what you're going to establish as your outcomes, as recorded in your CRF rather than reporting-

And that's kind of like, yeah, I mean, I wish I could say I've got. I've got a proper written, a plan of how this is all going to work. But, it would be really good to have a set of videos for each stage that people or something slides that people could easily refer to.
But we don't, which I guess is the simple answer here. We don't have that. And so, what I find is if someone has not got that previous experience of working with SAEs, they've worked on a trial where maybe they've had none. We have some trials that just don't have SAEs, they won't have SAEs because of the nature of the trial.
Then if they move to another trial, it's all new to them and therefore from my perspective is I'm kind of sitting down introducing them to it, which then comes down to that. You know, you'll ask them to review the SOP. Hopefully they might have already done that. But then you've got a hands-on kind of like right, are you doing this? Are you doing that? Which is already good and engaging.

But, I guess that that that the point there is it would be good to have better training in place and I think our limitation is the resource to put that together.
Yeah, yes, I was quite. That's my point now on training.

**Interviewer** 51:21
Thanks, S021. And just to just clarify a point, is this the training of staff within your CTU? Or sites or both.

**S021** 51:32
Primarily, yes, that that's the way I've perceived it, yeah.
Site training, I mean, depending on how you would to lay this out, you could certainly do it as some parts of it, the initial basic training I think which is just introducing I think would be useful for anybody. So, that would include sites but then sort of how you're managing it may be more locally focused and therefore more targeted at your trial teams and then the kind of like establishing your protocol and your safety reporting in there. I mean that's kind of targeting CIs isn't it, and TMs?
So, yeah, some of it would be. And I guess each, I mean in our project, in our trials, our studies, they should mostly have working instructions in place for how to do safety reporting at site, so that would probably constitute training for site staff for what we do.

**Interviewer** 52:34
Thank you, S021.

**S023** 52:42
So we would certainly cover safety reporting to sites during our site initiation visits. So, we do have slides about safety reporting and that's very study specific. It's about what's detailed in the protocol and we will have working practise documents with flow charts about what to do, when to do it type thing. But even though I know the trials I work on, I know the protocols inside out.
You think every time something comes in and a question comes in about safety report and I always have to go back to the protocol because they're all quite different from each other. What we're reporting, what we're not reporting. So, I always go back to the protocol, but we do train the sites during the site initiation visits.

In terms of the CTU, we have a lot of, again, working practise documents about how to actually do the process. So, we will have one about how to submit a SUSAR. You know screenshots of the screen, what to include where, we've got ones about doing the DSURs. So, we do have sign a kind of internal documents, with screenshots from all the websites, this is how you do it type thing.
And we do training, the trial managers would do the training in the individual safety reporting within their own protocols.

**S020** 53:54
Yeah, I'd just echo that. We try and make it as simple as possible, but again, it's only as good as your team at the site. How much money and how much money, how much time and capacity, how much money they give me and then, depends on how I report things. No, how much time and capacity they have to absorb the information you're giving them, especially if you have a particularly complex trial that has a difficult hard line of SAE reporting and it might be a little bit more nuanced and a little bit more, you know, then that's more time and to get them to engage properly with it in the context of a site induction visit or a training module can be quite difficult.
And as has been said before in this, sometimes you have to keep going over it as they as they do it, it's quite difficult to keep it top of mind depending on how complex your trial is and how busy the site the staff are, how experienced they are.

**S022** 54:50
I would agree. It's, we cover it in our SIV slides.
And I think there's a tendency, we've got a unit wide template for the SIV slides. I found pharmacovigilance tends to come quite late in that template. And as we're talking now I’m thinking, you know, it probably needs to come sooner just because I think by the end of an SIV, you know, particularly if it's, I don't know if it's better or worse, it being done remotely. But everyone in it's quite drained by the end of the SIV. So then when you come to pharmacovigilance it's, I think it maybe just doesn't get the time investment that it should have.
But I think the whole nature of this conversation really is it's well, how valuable is what we do in pharmacovigilance, anyway. So, I guess you could reprioritize it in your SIV, depending on how crucial you think it is in an early phase trial. Yeah, definitely. It's got to be really it's, point number one, probably for a phase one isn't it?

But, yeah, it's in our SIV. But, I feel like it's we're all quite knackered by the time we get to that point of going through the SIV slides and everyone's just tired and cheesed off and you know, so I want to maybe I'm just not very good at delivering them, which I think is probably, but it can be such a dry subject, can't it?

**S023** 56:15
So just thinking about our SIV slides, safety reporting does come nearer the end, and I think as you say, everybody's fatigued by that point. Including the person delivering the training.

**S022** 56:27
Yeah. Exactly. Fatigued, I like that word. That is it. Yeah. Better than cheesed off.

**Interviewer** 56:32
I like both. I think both because yeah, I was just thinking about trials I've worked on. And yeah, I think in SIVs I’ve done, pharmacovigilance and stuff has often came quite late. And I just wonder, do you think there's a relationship between the level of training and potentially, so I know that it was mentioned right at the beginning, about sort of inconsistencies and errors and things like that. And I just wonder how direct you think the relationship is between the training and the way it's delivered and the way that SAE forms are completed or the decisions that we make around safety reporting.

**S020** 57:14
I'm happy to respond to that initially. Definitely ,100%. If you've got a really snappy training module that's really easy to understand. You have a cute way of like presenting it or I think people like Monkey See monkey do they like to see what form it is. They like to see how examples might be filled in. Things like that are really, really good. But again, how much it's absorbed by the correct people at the site who are going to be doing it at the time, is really site dependent.

**Interviewer** 58:00
S021.

**S021** 58:04
Yeah, yeah, 'cause. I guess I have less hands on with site staff, so I suppose I don't know if I can input as well there. I was kind of when you asked me about when I was talking about the training. Well, what you know what part of the site staff and I guess that's in my perspective is more on getting our own trial staff up to scratch. But I think that's quite, it's a valuable point because what level of training can you do?

I mean, you mentioned pharmacovigilance is often at the end of slides which probably because of the timelines isn't it, like you recruit and then it's not until after you know what you set up a site, and then you do recruitment and all of those aspects. So, I guess that's probably why it often ends up near the end.
I'd be, something that I've come across is understanding certain sort of terminology, all the fields that you've got on forms.
We historically were using date deemed serious, but we found it was often getting miss- Well, certainly our understanding of it was different to a lot of, to many sites. Not all, but some. And I don't know if I go through it here might get a difference of opinion as I understood our understanding was that's the start date of the SAE, but many would see that as being when the PI deemed it, or perhaps in discussion if it was a potential SAE and they put in the date when it was confirmed. Oh no, this is a reportable SAE and I'd be like, no, no, it needs to be the start date. We have now changed that on our template forms and in our SOP.
But just some, yeah, simple things like that can cause a back and forth can cause delays because a site has a has, now whether that's training specifically, or whether it's just maybe, I mean I understood where the sites were coming from when they were mixing it up. So, the language being used on the form the field in the first place probably wasn't the best, but it came from somewhere. I think I struggled to identify where though in the like if it's in the Grey Book, or if it was somewhere where we decided date deem serious was the term to use as that is not very clear.
So yeah, yeah, I guess I'm saying is, I certainly agree that how the training is how it works and when you do it.
I know that we're slowly or we have been sort of once again risk proportionate with some of our trials, maybe not as much with safety reporting but with other areas I think.
Was elig- not sure. Not entirely sure, but some of our areas where we're because of the nature of the trial, so, any emergency care people are being doing a short training online signing off, they've done it, but they're not getting added to a delegation log. Now, that's not quite the same context here, but it's a change in our processes to move towards something which, it’s risk proportionate for the trial as well as supporting the nature of the trial.
But whether, I mean, I can't say if it's the best method going forward, but it reduces burden on sites which is valuable.
How that would work in a with safety reporting might depend on the context of the trial itself as well. You know if you could do a, if there's an easy access cut back form of training and that's all they needed to do to be able to say, fill out an SAE form. But I suppose at the end of the day still going to need your PI or delegate, who's appropriately trained, you know.
Perhaps full GCP rather than just some form of GCP training? Which seems to be coming more acceptable. Yeah, I’m kind of meandering now, sorry.

**S023** 1:01:58
But I don't think we make it easy for sites because I think as you say, terminology people, I mean it, different things by using different terms. But if everybody across all the CTUs were using the same terminology, so it doesn't matter what trial they were working on, they knew exactly what it meant. Then it would make it easier for the sites. So, same with the protocols where if you've got some sponsors that are more risk averse, the safety reporting will be different. What we do report, what we don't report. So, we don't make it easy for the sites because the protocols are all different. What we report is all different, the forms we ask then to complete are different. So, I think across a lot of the sites that I work on the teams will send an e-mail and ask do I need to report this because it's easier for them to do that quickly than to go and look OK, for this specific trial, what is it I'm doing? But, so, I think we could make things easier for ourselves and the site if we're consistent across the board.

**S022** 1:02:59
And I think another element of training that we haven't mentioned and I don't and this is, we don't have this sort of training and I don't know whether other units do and need or whether we need it, but whether we need to train like CIs almost as well in what pharmacovigilance in, according to our units definitions are and things like that as well, and train them in how to select which events that they feel need to be covered and things like that.

I feel like maybe some training because we were talking at the beginning about just the whole tasks involved in in pharmacovigilance and the first one being you decide what you are even going to report or what you're even going to look for, exactly. I just wonder whether that's the start of the training therefore as well, goes alongside that?

**S021** 1:03:56
I'll just jump in and say yeah, because that's one of the things I think is one of the areas. I agree with that one, because that helps establish your safety profile, then doesn't it? And what you need to report.
They will have a clinical, generally speaking, many of them will have a clinic, well, they'll have a lot of varied experience, but I think a lot of a clinical perspective. But it's also then more you need to have the clinical trial perspective of what you're doing. It's not just, you know.
I mean slight, slight, not quite tangent, but I mean, one of our let me just quickly check it. So, for the serious criteria, so assessing it, we've stated in our SOP that life threatening means at that time life threatening, not could have been life threatening if you didn't take action, that's something and because clinically you have a clinician will probably assume or many will assume it's likely what it could have been life threatening. And so, well, that's not what we're looking at, that's not what we're the classification is.
It's was it life threatening there and then, not if you didn't do anything, it would have been life threatening, because that could be true of anything. You know, any scenario could be eventually life threatening. So yeah, defining training CIs to understand safety pharmacovigilance is, I think, a key point.

**Interviewer** 1:05:28
Brilliant. Thank you very much everyone.
Yeah, the ambiguity around understanding and the way that things are interpreted being slightly different and the way that, you know, what we expect and how we interpret things within our CTUs and sponsors and different sites and things like that I think is an interesting kind of you know, how do you begin to combat that?
So, I am aware of the time, so I am just going to finish off with a over to you. What do you think is the point half? Is there anything that you're you know you have a burning desire to express about safety reporting that you've not had the opportunity to yet?

**S020** 1:06:19
I wish there were guidelines for sponsors to make it so that their interpretations were similar across the board. So that you don't have this these sort of challenges. I mean and it's not just when you work in that you know I know in [redacted] that they work with several different sponsors. You know the trials there, it doesn't automatically mean if you're in one university establishment, you're going to be working with that one and it's a good one.

So, I think, making it a clearer thing for everyone. Everyone kind of has to when they think about safety reporting has to like stop, take a breath, go through the definition in their head, decide which thing it goes into. It just, it feels difficult and there is always that element of anxiety that you've got it wrong, or you're not doing it right, or you're not reporting it enough, or then conversely, you're over reporting, wasting everyone's time, you've misunderstood you've, do you know what I mean?

And I think if I get that as a trial manager that's been in it for 20 years and I'm certain messages from sponsor tell me that they are also confused. And inconsistent reports from sites, despite our very best efforts at training with the best will in the world, suggest that they're also confused.

So, it's taking that, you know, we all know we have to do it, and we all want to get it right. So, it's just making it much simpler would be really good. I don't know how you could do that. That's not a moan. It's just an observation.

**Interviewer** 1:07:58
It’s also fine if it is a moan. I'm OK with moaning.

**S022** 1:08:05
So, I think I'd just like to raise because I think we've been talking a lot about the sort of day-to-day well, not necessarily the day-to-day, but you know because we've talked about the trial design thing as well. But I just want to pick up about our DSUR experience as well really and just see whether other people, how are the people have experienced this or whether they have or not.

So, we have a template for our DSUR and in it it's got various narrative sections about this is what should be included, and the wording that's included in what's in the section is to be completed by the CI of overall safety assessment.
Where it describes it as an overall safety assessment. So, this is taken from
E2F notes for guidance on DSUR reporting “the overall safety assessment should be a concise integrated evaluation of all new relevant clinical non-clinical and epidemiological information, obtained during the reporting period relative to previous knowledge of the investigational drug. The assessment should consider cumulative experience, new information collected in the period collected by the covered by the DSUR and for investigational drugs for the marketing approval, clinically significant post-marketing data” and so on. It goes on. And so, then our guideline says that this CI should be completing this section. And now I took our guideline very, very literally because I have the section of our trials unit I work in don't normally do CTIMPs anymore, for historical reasons we did end up doing this particular one.
And so, I think other divisions in the trials unit don't necessarily, I don't know whether you all did the same thing, but sometimes if you're used to doing a process such as writing your DSUR, you'll go back to following the process that you did for every other DSUR you've done previously. I've not done them. So, I go and look very, very specifically at the template where it says that the chief investigator should be completing these sections. So, I forward it to the to the chief investigator and she starts panicking and doing Medline searches for all new information that has come up about these particular medications, which I've so been in use since the 50s. And
even slimming it down, I can't remember how she managed to do that, but it was still, she knew was going to have to potentially be over 2000 articles to be able to provide what she interpreted of that section.
And I just wondered for other trials units, have you come up against a similar thing? Do you have chief investigators do it or you - I think a lot of the time our trial managers or data managers are writing that section themselves. And because if nothing in significant has occurred in the trial, they're just saying there's nothing new to report, whereas our chief investigator, because I shipped it off to her.
She's not trained, really, not done anything like this before. Then if she's interpreted to the letter.
Have anyone else experienced that when they come to the DSURs or is everyone just everyone's role mainly just doing the day-to-day? SAE mechanics, you know what I mean. I'm just wondering how people go about, when they come to the DSUR, completing that section.

**S023** 1:11:43
Which section was it?

**S022** 1:11:45
It's called overall safety assessment.

**S023** 1:11:47
Overall safety, so for the CTIMP I work on, I do the DSUR and send it to investigator and ask them to check. Are you happy with everything I've written.
And I certainly I do, a literature search certainly to see what's come out in the past year. But I've never come across like 2000 articles, but normally you know maximum, maybe twenty articles. I need to go look at. I don't know if it's just specifically the drug that you're that you're using.
But I do do a literature review just to see what's come out in the year. Yeah, I tend to do the DSUR and then send it on and ask, are you happy with this? And if there's any specific comments, you know, that might be a question I would highlight to say, can you just, you know, don't just skip through this one, make sure that you're absolutely happy with what I put in this section.

**S022** 1:12:17
Yeah, it's really useful to you.

**S023** 1:12:36
I've never sent it. I've never sent it to the chief investigator to complete.

**S022** 1:12:41
No, I mean as I say, no one else in I does, but that's what the guideline says to do. So, I just did it.

**S023** 1:12:44
Yeah. Yeah, yeah.

**S020** 1:12:47
It is ultimately CI responsibility. I think it's a delegated responsibility to the CI. They have the ultimate they hold the ultimate string with responsibility for it, just as they hold the ultimate purse string for the grant, but they never do it.

**S023** 1:12:49
Yeah.

**S023** 1:13:03
Yeah, in our case she did.

And she I don't think she ended up reviewing 2000. She might obviously, she managed to slow it down in her own head in a little bit, but yeah, it just struck me that actually going back to the risk proportionate, there's nothing anywhere. None of the GCP guidance does slim that down, that section say, well, you don't really need to report all these things for a really well-established drug.
So just wondering if anyone has come up against it?

**S020** 1:13:38
Not at all. Never in the like of that many articles at all, like there might be like one extra thing a year. But that someone's, usually I don't even search. Usually, I just ask. I ask the PMG or the TSC, because they often know what's out.

**S021** 1:13:39
No. Yeah.

**S022** 1:13:39
Yes. Yeah, exactly. That's what I'm my gut feeling. So, I think I did do them years ago on an old trial and that was the approach I took.

**S020** 1:14:01
Yeah.

**S022** 1:14:02
But being on out of the loop of it, and looked into our guidelines and what we do in our guidelines is what our guidelines say is not what anyone actually does. So, it's good to know that actually.

**S020** 1:14:14
She sounds like a good CI pass her on when you're finished with her. She sounds really good.

**S022** 1:14:20
Anxiety provoking. But no. No, she's very thorough. And she's lovely. She's, you know, we're so we're so lucky to have her because, you know.

**S020** 1:14:20
Sounds really nice to work with.

**S022** 1:14:29
That's a different story, isn't it? A nightmare CI.

**S020** 1:14:31
It's a rare find.

**S022** 1:14:34
Yeah.

**Interviewer** 1:14:34
So that's another focus group itself, isn't it?

**S020** 1:14:36
Yeah.

**S022** 1:14:38
Yeah. No, no, it's been really useful to just understand other trials units approaches as well.

**S021** 1:14:45
Yeah, I was just looking and oh, sorry, you want to finish

**S022** 1:14:46
I was just going to say so, S020, you say that you acknowledge it's CI responsibility, but you don't actually have the CIs do it. Yeah, right.

**S020** 1:14:56
Not in my, not in my personal experience of when I've done them, which has only been a few, but no.

**S022** 1:15:03
Thank you.

**S021** 1:15:05
I was going to say I think could welcome down to CIs, doesn't it? I mean, that's a general experience from our trials as each CI is different and how they manage and oversee a trial will vary per, I think I was just looking because we had one recently for a DSUR and which, as an aside, it was a type A and I think last year didn't they say Type A’s only had to do the APR.
And then literally at the start of Feb, turn around and said no, all Type A’s now need DSUR again. And they were, yeah. Just came out and. Yeah. So, they had, like, less than a week to put together the DSUR which, which wasn't a lot of fun, but that's another point, I guess.

**S022** 1:15:37
Did they say that?

**S021** 1:15:51
But it looks like the CI was supporting the TM and the lead pharmacist and someone else, so I'm not sure who they were, but they all engaged on it to help put it together. Partly that might have been because of the rush, but I'm not sure though what our standard is. I think it could somewhat come down to the SOP I'm sure will say something, but also, I suspect it's a collaborative effort. It will generally be we don't have a lot of them though. As I say, we don't do too many CTIMPs these days.

**Interviewer** 1:16:33
Thank you everyone. Did anyone have anything else that they wanted to add before I close the group.

**S022** 1:16:45
I think it's been really, really interesting.

**Interviewer** 1:16:48
Thank you. I'm glad that it's been useful and interesting for all of you. I'm going to stop the recording.


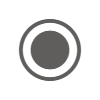
 **Interviewer** stopped transcription
