# Supplementary material for: Walking the tightrope of justifiable decision‑making: An exploratory qualitative study identifying barriers and solutions to efficient safety reporting
Source: PLoS One. 2026 Jul 30;21(7):e0354806. doi: 10.1371/journal.pone.0354806 (PMC13422843; doi:10.1371/journal.pone.0354806)
Supplement: S6 Appendix — (DOCX) [file pone.0354806.s006.docx]

**Resources and training for new clinical trials regulations**

1. **The MHRA regulations webinar series** (41),

MHRA. https://www.gov.uk/guidance/clinical-trials-regulations-webinar-recordings [Internet]. 2026 [cited 2026 Jun 3]. Guidance: Clinical Trials Regulations webinar recordings. Available from: <https://www.gov.uk/guidance/clinical-trials-regulations-webinar-recordings>

1. **HRA Regulations Reform Hub (42),**

HRA. https://www.hra.nhs.uk/planning-and-improving-research/policies-standards-legislation/clinical-trials-investigational-medicinal-products-ctimps/clinical-trial-regulations-reform/ [Internet]. 2026 [cited 2026 Jun 3]. Clinical Trials regulations reform. Available from: <https://www.hra.nhs.uk/planning-and-improving-research/policies-standards-legislation/clinical-trials-investigational-medicinal-products-ctimps/clinical-trial-regulations-reform/>

1. **HRA’s updated guidance on Safety reporting (43)**

HRA. https://www.hra.nhs.uk/approvals-amendments/managing-your-approval/safety-reporting/ [Internet]. 2026 [cited 2026 Jun 3]. Safety Reporting. Available from: <https://www.hra.nhs.uk/approvals-amendments/managing-your-approval/safety-reporting/>

1. **ICH GCP E6 R(3) safety guidelines training modules(44).**

ICH. https://www.ich.org/page/training-library. 2026. ICH Training Library.
