# Supplementary material for: Walking the tightrope of justifiable decision‑making: An exploratory qualitative study identifying barriers and solutions to efficient safety reporting
Source: PLoS One. 2026 Jul 30;21(7):e0354806. doi: 10.1371/journal.pone.0354806 (PMC13422843; doi:10.1371/journal.pone.0354806)
Supplement: Attachment — Submitted filename: THOMPSON et al - response to reviewers 01-Mar-26.docx [file pone.0354806.s008.docx]

**Response to reviewers: Walking the tightrope of justifiable decision making: An exploratory qualitative study identifying barriers and solutions to efficient safety reporting**

| **Comment** | **Response** |
| --- | --- |
| **Reviewer 1** |  |
| Conceptual focus and originality: The manuscript’s central theme—balancing efficiency with regulatory compliance—has been explored in prior qualitative and policy analyses. However, the contribution of this study relative to existing literature (e.g., Perez et al., 2017; Seltzer & Bhattacharyya, 2018) remains insufficiently articulated. The framing does not clearly demonstrate how this work advances current understanding of risk-adapted approaches or decision-making within CTUs. | We thank the review for these comments.  The introduction has been re-structured and substantially expanded to better frame and highlight the contribution and position of this work within the wider context. |
| Methodological depth: Although Reflexive Thematic Analysis is appropriate, the description of analytic rigor is limited. The coding process, theme development, and strategies for ensuring analytic credibility (e.g., triangulation, member checking) are not sufficiently evidenced. The thematic structure appears largely descriptive, with limited interpretive depth or theoretical integration. | Further details have been added to the method section (specifically the analysis section), enhancing the description of the analysis and how analytic rigour was employed.  Information about the coding and theme development and reflexive practice has been added.  Member checking and triangulation were not performed as these processes are incongruent with the approach to Reflexive Thematic Analysis taken in this study. Instead, analytic rigour was ensured through functional transparency and reflexive practices, including documentation and speaking with the research team and other researchers with qualitative experience. These decisions and their justification are also outlined in the Analysis section and supported with relevant references,  The aim of this study was primarily descriptive and was not seeking to generate new theory. The results and thematic structure have been revised to outline the interpretation of the data more clearly and highlight the depth of interpretation in line with regulatory guidelines and policy. |
| Researcher positionality and reflexivity: The lead author’s close professional association with some participants introduces potential bias that is acknowledged but not adequately mitigated. Greater transparency about how reflexivity was incorporated into the analytic process—beyond acknowledgment of awareness—would be essential to ensure trustworthiness | We particularly appreciate the highlighting of this point. Description of positionality and reflexivity have been revised in the methods section. The types of questions asked to enable reflection and the discussions between members of the research team are highlighted in the analysis section.  Additional reflexive commentary is also added to the participants, recruitment and informed consent section in relation to sample size and how these decisions were made.  Previous versions of thematic maps have been added to the supplementary materials (S4 appendix) to demonstrate the process of theme generation more transparently.  There has also been reflection on potential biases added to the limitations section. |
| Sample representativeness and saturation: The sample is heavily weighted toward oncology and academic CTUs, which restricts generalizability. Although “information power” is cited to justify sample size, this rationale is not convincingly demonstrated with respect to data richness or thematic sufficiency. | We agree with the reviewer’s reflections on generalisability and sample weighting. Given the contexualist position of this study, we have chosen to consider transferability of findings, rather than generalisability. This has been highlighted in the participants, recruitment and informed consent section.  The issue of the weighting of the representation of staff with experience in oncology trials is now included in the limitations section.  The details about information power and how this was reached has been expanded and clarified in the participants, recruitment and informed consent and analysis sections in the method.  Limitations of the sampling methods have also been added to the limitations section.  The process undertaken to reach thematic sufficiency has been addressed through the explanation of the analytic process in the Analysis section of the methods. |
| Analytical and structural concerns: The over-arching theme of “Walking on a tightrope” is conceptually strong but lacks sufficient analytical integration among subthemes. Several findings are paraphrased rather than synthesized | The results section has been revised to better integrate the subthemes within the main theme and highlight the central organising concept of balancing risk. |
| The discussion section reiterates results rather than advancing a deeper conceptual model of decision-making or systemic reform. | The discussion has been reconfigured to align more closely with the re-structured results section. The discussion also now focuses more on interpretation of the findings, positioning them in the wider context of existing literature and advancement of practice. |
| Presentation and formatting: The manuscript contains formatting inconsistencies, lengthy quotations, and a very detailed methods section that could be condensed. | We have addressed these points, capturing formatting inconsistencies and reducing length of quotes where possible, while noting that reviewer 3 indicated the quotes were effectively integrated into the narrative. Reductions have tried to avoid loss of information that would affect their meaning or demonstration of the richness of the data being represented. Additionally, the methods have been condensed where possible. However, there are some areas where detail remains to ensure that the analytic process is being explained with sufficient transparency. |
| Figures 1 and 2 are underdeveloped and do not clearly convey analytical structure. | Both figures have been updated, as per suggestions. Figure 1 now includes more detail about the analytic process. Figure 2 has been revised to demonstrate the thematic structure. |
| The reference list also requires standardization (missing DOIs and inconsistent citation formatting). | The references have been updated for consistency and DOIs have been included where possible/available. |
| **Reviewer 2** |  |
| This is a written and timely qualitative study on barriers and potential solutions to efficient safety reporting in CTIMPs, from the perspective of UK academic CTU staff. The focus on the “tightrope” of justifiable decision-making is compelling, and the five subthemes map well onto current debates about risk-proportionate approaches and bureaucratic burden. The paper is generally well structured, with appropriate use of reflexive thematic analysis and effective integration of participant quotes into the narrative. It has clear relevance for regulators, academic sponsors, and CTUs. | We thank reviewer 2 for their positive review. |
| **Reviewer 3** |  |
| Introduction and Objectives:  The introduction should clearly define the existing gap in knowledge regarding safety reporting in clinical trials. The manuscript does not provide a thorough review of past research to contextualize its study objectives. It is recommended that the authors expand the literature review, highlighting specific unresolved challenges and justifying why this study is necessary. | Thank you for all of these comments We have expanded the literature review to better illustrate where issues with safety reporting have arisen and the knowledge gaps we sought to address through this work. |
| Methodology – Sampling:  The rationale for using convenience sampling is not adequately explained. The authors should clarify why this approach was chosen and discuss its limitations, particularly in terms of the potential for sampling bias and limited generalizability. Explicit acknowledgment of these limitations and any efforts to mitigate them would strengthen this section. | The justification the use of convenience sampling and the mitigations put into place to manage limitations of this approach have been clarified in the participants, recruitment and informed consent section of the method. Given the contexualist position of this study, we have chosen to consider transferability of findings, rather than generalisability. This has also been highlighted in the participants, recruitment and informed consent section in the methods.  Limitations of this approach to sampling are now outlined in the limitations section towards the end of the discussion. |
| Discussion – Comparison with Previous Work:  The discussion section should systematically compare the study’s findings with those of previous research. Clearly identifying both similarities and differences will reinforce the credibility of the findings, show how they support or challenge past studies, and better highlight the novel contributions of this work. Where new insights are presented, explicitly detail how these differ from or build upon previous literature. | The discussion has been re-written to align with changes to the results section and to more clearly focus on the central concept of balancing decision-making.  Comparisons with previous literature and their similarities and challenges have been made more clearly and systematically.  The contributions to the field made by this study have been made more explicit. |
| **Editor Notes** |  |
| Please ensure that your manuscript meets PLOS ONE's style requirements, including those for file naming. | We confirm that the manuscript meets PLOS ONE’s style requirements. |
| We note that the grant information you provided in the ‘Funding Information’ and ‘Financial Disclosure’ sections do not match.  When you resubmit, please ensure that you provide the correct grant numbers for the awards you received for your study in the ‘Funding Information’ section. | The funding disclosure information has been updated to ensure that the sections match. |
| We note that one or more of the authors are employed by commercial companies: Astellas, Janssen, Sanofi-Aventis, Eisai, and Eli-Lily. Please provide an amended Funding Statement declaring this commercial affiliation, as well as a statement regarding the Role of Funders in your study. If the funding organization did not play a role in the study design, data collection and analysis, decision to publish, or preparation of the manuscript and only provided financial support in the form of authors' salaries and/or research materials, please review your statements relating to the author contributions, and ensure you have specifically and accurately indicated the role(s) that these authors had in your study. You can update author roles in the Author Contributions section of the online submission form. | These organisations did not play any role in this work or publication. The funding statement has been amended and the author contributions section revised. |
| Please also include the following statement within your amended Funding Statement. “The funder provided support in the form of salaries for authors [insert relevant initials], but did not have any additional role in the study design, data collection and analysis, decision to publish, or preparation of the manuscript. The specific roles of these authors are articulated in the ‘author contributions’ section.” If your commercial affiliation did play a role in your study, please state and explain this role within your updated Funding Statement. | The funding statement has been amended to indicate that commercial affiliations played no role in the study. This has been added to the updated cover letter. |
| Please also provide an updated Competing Interests Statement declaring this commercial affiliation along with any other relevant declarations relating to employment, consultancy, patents, products in development, or marketed products, etc.  Within your Competing Interests Statement, please confirm that this commercial affiliation does not alter your adherence to all PLOS ONE policies on sharing data and materials by including the following statement: "This does not alter our adherence to PLOS ONE policies on sharing data and materials.” (as detailed online in our guide for authors http://journals.plos.org/plosone/s/competing-interests) . If this adherence statement is not accurate and there are restrictions on sharing of data and/or materials, please state these. Please note that we cannot proceed with consideration of your article until this information has been declared. | An updated competing interests statement has been included in the updated cover letter and the statement regarding commercial affiliations has also been included. |
| Please include both an updated Funding Statement and Competing Interests Statement in your cover letter. We will change the online submission form on your behalf. | The updated funding statement and competing interests statement have been included in the updated cover letter. |
| We note that this data set consists of interview transcripts. Can you please confirm that all participants gave consent for interview transcript to be published? | Participants did not provide consent for transcripts to be published. However, the transcripts are provided for the purpose of transparency, as required by the journal. The provided transcripts have been redacted (all identifying features such as trial names, places and names have been removed) and participants pseudonymised to ensure that participants cannot be recognised, and their anonymity is maintained. |
| If the reviewer comments include a recommendation to cite specific previously published works, please review and evaluate these publications to determine whether they are relevant and should be cited. There is no requirement to cite these works unless the editor has indicated otherwise. | N/A – no recommendations made |
