# Supplementary material for: Walking the tightrope of justifiable decision‑making: An exploratory qualitative study identifying barriers and solutions to efficient safety reporting
Source: PLoS One. 2026 Jul 30;21(7):e0354806. doi: 10.1371/journal.pone.0354806 (PMC13422843; doi:10.1371/journal.pone.0354806)
Supplement: Attachment — Submitted filename: THOMPSON et al Response to reviewers June 26.docx [file pone.0354806.s009.docx]

**Response to reviewers: Walking the tightrope of justifiable decision making: An exploratory qualitative study identifying barriers and solutions to efficient safety reporting**

| **Comment** | **Response** |
| --- | --- |
| **Reviewer 2** |  |
| The manuscript is suitable for publication after minor revision. It makes a meaningful contribution to understanding why risk-proportionate safety reporting remains difficult to implement in practice. With clearer articulation of its contribution, stronger connection to UK regulatory reforms, and more actionable recommendations, the manuscript will be highly valuable for trial methodologists, regulators, sponsors, and academic clinical trials units. | We thank the reviewer for this comment. Please see responses outlined below:  **Clearer articulation of contribution**:  The contribution of this work has been clarified in the final paragraph of the introduction and re-iterated in the conclusion.  **Connection to regulatory reforms:**  This has been addressed with additional text in the introduction and discussion which re-frames the position of the work in relation to the regulatory updates.  **Actionable recommendations:**  The recommendations made in Box 1 have been updated to be more actionable, in line with the newly implemented regulations.  Additional supporting references have been included where required. These are highlighted in the reference list.  **Additional amendment:**  An additional point has been added to the limitations section regarding the timing of the interviews in relation to the implementation of new regulations. |
